# Sequential analysis of myocardial gene expression with phenotypic change: Use of cross-platform concordance to strengthen biologic relevance

**DOI:** 10.1371/journal.pone.0221519

**Published:** 2019-08-30

**Authors:** Lee S. Toni, Ian A. Carroll, Kenneth L. Jones, Jessica A. Schwisow, Wayne A. Minobe, Erin M. Rodriguez, Natasha L. Altman, Brian D. Lowes, Edward M. Gilbert, Peter M. Buttrick, David P. Kao, Michael R. Bristow

**Affiliations:** 1 Division of Cardiology, University of Colorado, Denver/Anschutz Medical Campus, Aurora, Colorado, United States of America; 2 ARCA biopharma, Westminster, Colorado, United States of America; 3 Department of Pediatrics, University of Colorado, Denver/Anschutz Medical Campus, Aurora, Colorado, United States of America; 4 University of Colorado Cardiovascular Institute Pharmacogenomics, Boulder and Aurora, Colorado, United States of America; 5 Division of Cardiology, University of Nebraska Medical Center, Omaha, Nebraska, United States of America; 6 Division of Cardiology, University of Utah Medical Center, Salt Lake City, Utah, United States of America; Maastricht University, NETHERLANDS

## Abstract

**Objectives:**

To investigate the biologic relevance of cross-platform concordant changes in gene expression in intact human failing/hypertrophied ventricular myocardium undergoing reverse remodeling.

**Background:**

Information is lacking on genes and networks involved in remodeled human LVs, and in the associated investigative best practices.

**Methods:**

We measured mRNA expression in ventricular septal endomyocardial biopsies from 47 idiopathic dilated cardiomyopathy patients, at baseline and after 3–12 months of β-blocker treatment to effect left ventricular (LV) reverse remodeling as measured by ejection fraction (LVEF). Cross-platform gene expression change concordance was investigated in reverse remodeling Responders (R) and Nonresponders (NR) using 3 platforms (RT-qPCR, microarray, and RNA-Seq) and two cohorts (All 47 subjects (*A-S*) and a 12 patient “Super-Responder” (*S-R*) subset of *A-S*).

**Results:**

For 50 prespecified candidate genes, in *A-S* mRNA expression 2 platform concordance (C_cpT_), but not single platform change, was directly related to reverse remodeling, indicating C_cpT_ has biologic significance. Candidate genes yielded a C_cpT_ (PCR/microarray) of 62% for Responder vs. Nonresponder (R/NR) change from baseline analysis in *A-S*, and ranged from 38% to 100% in *S-R* for PCR/microarray/RNA-Seq 2 platform comparisons. Global gene C_cpT_ measured by microarray/RNA-Seq was less than for candidate genes, in *S-R* R/NR 17.5% vs. 38% (P = 0.036). For *S-R* global gene expression changes, both cross-cohort concordance (C_ccT_) and C_cpT_ yielded markedly greater values for an R/NR vs. an R-only analysis (by 22 fold for C_ccT_ and 7 fold for C_cpT_). Pathway analysis of concordant global changes for R/NR in *S-R* revealed signals for downregulation of multiple phosphoinositide canonical pathways, plus expected evidence of a β_1_-adrenergic receptor gene network including enhanced Ca^2+^ signaling.

**Conclusions:**

Two-platform concordant change in candidate gene expression is associated with LV biologic effects, and global expression concordant changes are best identified in an R/NR design that can yield novel information.

## Introduction

Heart failure is a consequence of structural and functional impairments of ventricular myocardium that reduce the ability of the heart to adequately supply blood to the body [1]. The current method of classifying heart failure is by whether left ventricular ejection fraction (LVEF) is reduced (“HFrEF”) or relatively preserved (“HFpEF), which generally subdivides heart failure into respective categories that respond to multiple types of drug or device therapies vs. those that do not [2]. Despite the established effectiveness of 8 drug and 2 device classes [3], HFrEF continues to increase in prevalence [4] and major adverse outcomes including mortality have not appreciably declined [5].

Current drug therapy of HFrEF is based on targeting final common pathway maladaptive mechanisms that provide short term compensation to impaired ventricular chamber contractile function, but have adverse effects long term [3]. Despite the relative success of this therapeutic approach, the molecular mechanisms underlying the progression of HFrEF as well as the molecular phenotypic signatures of effective therapies have not been established. Reduced to first principles, disease in any tissue is the result of altered gene expression that produces organ dysfunction, and therefore the causative mechanisms are discoverable by identifying abnormal patterns of gene expression directly related to the dysfunction. In HFrEF we have attempted to identify abnormal gene expression profiles at the mRNA [6–8] or miRNA [9] level by producing therapeutic interventional reversal of the structural and functional abnormalities in HFrEF ventricles, and identifying ventricular septal endomyocardial biopsy gene expression changes associated with dynamic left ventricular reverse remodeling. This approach, which we term “sequential analysis of myocardial gene expression with phenotypic change” (SAMGE-PC), has several potential advantages, including: investigating serial changes in subjects whose genetic and non-interventional therapeutic backgrounds remain constant before and after ventricular reverse remodeling; being able to link changes in gene expression to specific functional and structural changes in ventricular myocardium; having an internal control group consisting of reverse remodeling nonresponders; being able to investigate HFrEF at earlier stages than can be done by using starting material from explanted hearts removed from cardiac transplant recipients; and the inherent statistical advantage of within group repeated measures vs. cross-sectional comparisons [7].

In these studies, for mRNA expression we have used two types of quantitative PCR [6,8,9], two generations of microarrays [7–9] and most recently, RNA-Sequencing (RNA-Seq) [10]. Each of these three methodologies has been widely used for quantifying mRNA expression, and all have undergone extensive validation, typically in “cross-platform” analyses (11–13). However, the inherent methodological differences in these platforms as well as limitations in starting material means that in sampling human tissue *in situ* platform agreement may be lower than in model systems [14,15]. Using a repeated measures longitudinal design helps counter these limitations [16], as does the use of a restricted candidate module with fewer genes and higher probability of change, but these may not be adequate to address inherent methodological challenges. Another design feature with the potential to improve reproducibility and reduce false discovery is the deployment of multiple platforms in contemporaneous measurements, allowing for cross-platform validation [17–19]. The primary aim of the current report was to investigate the utility of cross-platform validation of gene expression changes in the setting of a SAMGE-PC study, with a secondary aim of evaluating the effects of downsizing sample size into a greater phenotypic change cohort.

## Materials and methods

### Study material and datasets

#### Clinical study

The data are derived from the Beta-blocker Effect On Remodeling and Gene Expression study (“BORG”, NCT01798992), the full design of which is described elsewhere [8]. Briefly, eligible idiopathic dilated cardiomyopathy (IDC) patients with HFrEF (LVEF ≤40%, symptomatic heart failure) were treated with one of three combinations of β-adrenergic receptor (AR) antagonists that had β_1_-AR blockade in common [8]. RV distal septal endomyocardial biopsies were performed prior to and after 3 and 12 months treatment with these regimens as previously described [8]. In the current analysis the 12 month results are used unless data were unavailable (n = 8), in which case 3 month data were used by last observation carried forward (LOCF) [8]. The primary method of measuring LV remodeling was by ejection fraction (EF, a measure of chamber structure and function [3]) by radionuclide SPECT imaging [3,8] for determination of LVEF or RVEF. All patients signed written consent for this multi-center study, which was conducted at the University of Colorado Anschutz Medical Campus and the University of Utah Medical Center and approved by the Institutional Review Boards at both sites. The study was conducted according to the Declaration of Helsinki and included a Data and Safety Monitoring Board.

#### RNA extraction and mRNA abundance measurements

RNA extraction was performed on RV distal septum endomyocardial biopsy samples as previously described [8]. All mRNA measurements were from the same RNA extraction, typically involving 2–5 separate biopsy samples from an individual patient. Extracted RNA was stored at -80°C until use. Gene expression measurements were conducted by 3 platforms: TaqMan RT-qPCR (for 50 candidate genes) [8]; microarray (for global as well as candidate gene expression); and RNA-Seq (global and candidate gene expression). Candidate genes were selected for the likelihood they may mediate myocardial remodeling in response to neurohormonal activation or inhibition, within paradigms of either fetal/adult expression patterns, β_1_-adrenergic receptor signaling or thyroid hormone mediated effects [8]. RT-qPCR and microarray analyses were performed as previously described [8]. The microarray method was the Affymetrix HG-U133 Plus 2.0 Microarray that contains sequences for 22,963 separate gene sequences, with which 19,672 separate mRNAs were identified, including for all 50 of the candidate genes. RNA-Seq was conducted as previously described [10], using an Illumina HiSeq2500 platform and ribosome depleted RNA. RNA-Seq identified 15,366 separate gene transcripts, including the 50 candidate genes. RT-qPCR Ct values were normalized to GAPDH gene expression. Normalized changes in RT-qPCR gene expression from baseline to LOCF were calculated with the ddCt_._ method as previously described [8]. Microarray gene expression data were normalized by log-scale robust multi-microarray analysis. RNA-Seq data transcript levels were quantified as fragments per kilobase of exon per million mapped reads (FPKM) as previously described [10].

#### Patient cohorts and reverse remodeling phenotypic groups

Two patient cohorts were analyzed: the BORG IDC cohort of 47 subjects, (“*All-Subjects”* (*A-S*)); and an *A-S* derived subcohort of the largest degree of reverse remodeling responders and control nonresponders (“*Super-Responder”* restricted *(S-R)* cohort). The 3 LV phenotypes as defined by their LVEF change measured at LOCF are therefore Responders (R) and Nonresponders (NR) in the *A-S* cohort, and Super-Responders (SR) in the *S-R* cohort. SRs have their own NR analysis group, which is the same phenotype as *A-S* NRs.

*A-S* Cohort. This IDC cohort consisted of 31 reverse remodeling Responders (R, n = 31) defined by an LOCF increase in LVEF of ≥5 absolute % at 3 months or 8% at 12 months, and 16 Nonresponders (NR) [8]. In R the baseline LVEF/RVEFs in %±SD were 25.6± 8.2/27.0± 8.7, and in NR 27.8± 9.9/28.2/9.5 (R vs. NR P = 0.42 in LVEF, 0.71 in RVEF). The LOCF—baseline absolute % increase in LVEF (mean±SEM) was 21.2±1.8 in R, vs. 0.9±1.1% in NR (P <0.0001). The absolute % change in RVEF was 9.7±2.2 in R, vs. 5.2±3.8 in NR (P = 0.30).

*S-R* Cohort. In this cohort “Super Responders” (SR, n = 6) were defined by an increase in LVEF of ≥10 absolute % (mean±SEM increase by 31±4%, P = 0.035 vs. *A-S* cohort R), and NRs (n = 6) by age and gender-matched patients with no LVEF increase ≥5% (mean change 0±2%, P <0.0001 vs. R). The mean±SD baseline and LOCF LVEFs in the *S-R* cohort were respectively 32± 9% and 32±8% in NR, and 24±7% (P = 0.63 vs. *A-S* R baseline) and 53±6% (P <0.001 vs. *A-S* R LOCF) in SR. RVEF mean change±SEM in SR was 16.4±8.1%, vs. 7.5±6.7% in NR (P = 0.42).

#### Summary of gene analysis groups, platforms and patient cohorts

**Fig 1:**
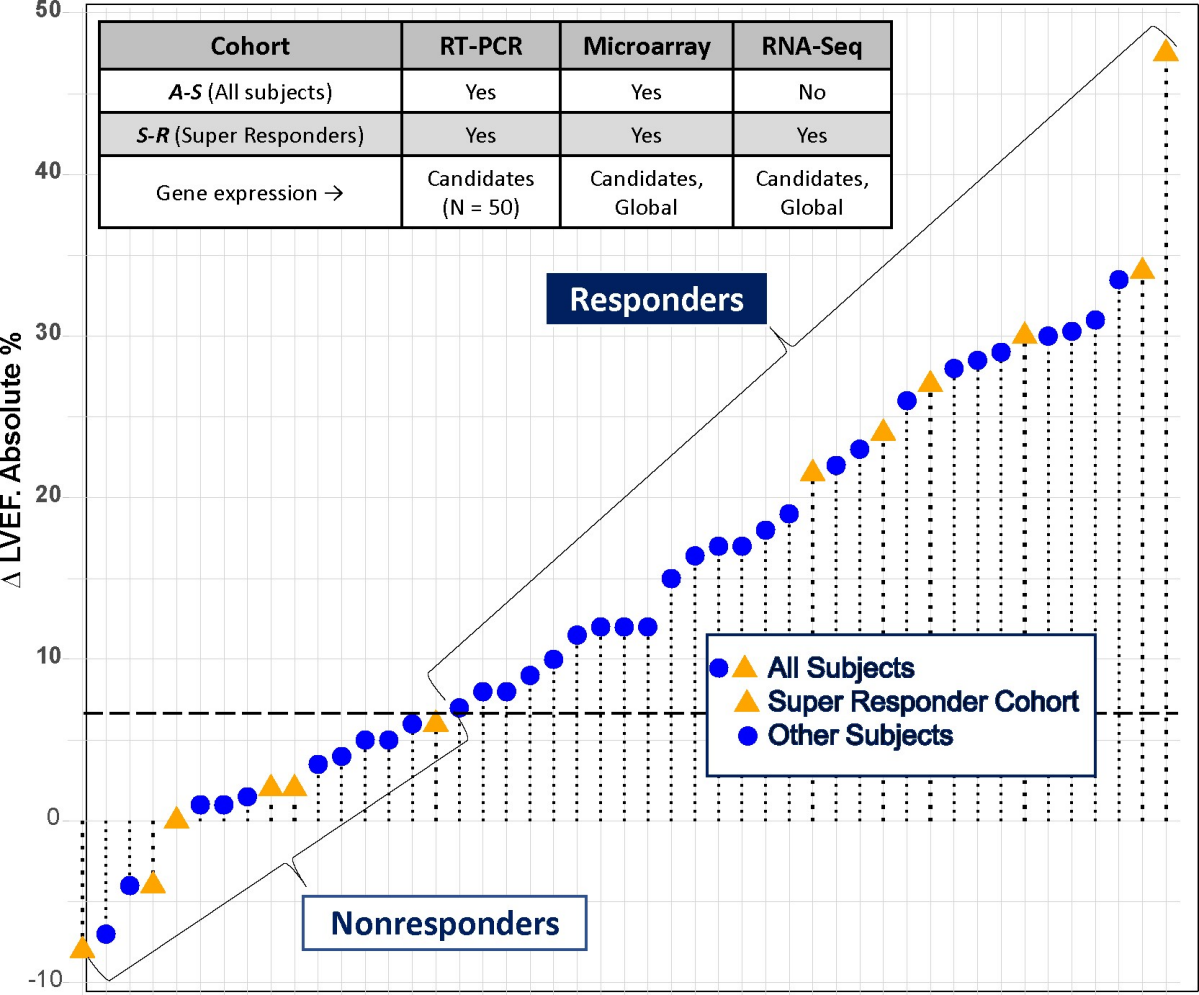
Summary of LVEF changes from baseline. LVEF change from baseline in the 47 patients comprising the All-Subjects (*A-S*) Cohort, and the 12 Subjects in the Super-Responder (*S-R*) subcohort. Messenger RNA measurement platforms, Responder/Nonresponder reverse remodeling phenotype and types of gene expression changes measured in each platform are also given.

Fig 1 summarizes the platforms, patient cohorts, reverse remodeling phenotypes and types of gene expression analyses (candidate or global) investigated. A data table roadmap is given in the Supporting Information (S1 Table).

### Data analyses

#### Data analysis within reverse remodeling groups

Changes in gene expression measured as mRNA abundance were assessed within R, SR or NR reverse remodeling groups as change from baseline, using paired analyses of baseline vs. LOCF values of dCt for RT-qPCR, fluorescence intensity units for microarray and FPKM for RNA-Seq. Thus, the primary analysis in each phenotypic group was conducted with the untransformed readouts from each platform. The R/NR analysis was conducted by comparing the subtracted (LOCF–baseline) values in Rs to NRs. Fold changes for RT-qPCR and microarray data were determined by averaging the 2^subtracted values (2^-ddCt^ for RT-qPCR) from each patient. For RNA-Seq FPKM, which is not an exponentiated value as for RT-qPCR and microarray data, the within R (or SR) and NR groups average fold change was determined by subtracting log2 transformed baseline values from the LOCF values for each patient, and then converting the difference to the base 2 antilog, i.e. 2^((Log2 LOCF FPKM)—(Log2 baseline FPKM)) and averaging for all members of the group. Fold change for R/NR was calculated by dividing the R by the NR fold change.

#### Statistical tests

Baseline to LOCF changes in mRNA abundance within Responder (R) and Nonresponder (NR) groups were assessed by the Wilcoxon signed-rank test. Between group changes (R/NR analysis) were determined by Wilcoxon rank sum test. LVEF and RVEF were analyzed by t-tests, as the data were not significantly non-normally distributed by the Anderson-Darling test. Potential differences in categorical variables were assessed by Chi-Square or Fisher’s exact test for small cell sizes, with a Benjamini-Hochberg [20] adjustment for multiplicity. Alpha was set at 0.05 (2-sided P <0.05) for selection of differentially expressed genes (DEGs) on changes from baseline within R or NR groups, and in R/NR analyses. Rank correlation analysis was performed using Spearman’s Rho. Statistical analyses were performed on the R statistical package [21].

#### Cross-platform or cross-cohort concordance

Concordant change was defined as the same directional change at a P <0.05 in each compared platform. *Total Cross-Platform Concordance* (C_cpT_) in % for mRNA abundance changes (up plus down-regulated genes, “DEGs”) between platforms within the same cohort (*A-S* or *S-R*) and response (NR, R or R/NR) was calculated from the formula [22]

$$C_{\mathrm{cpT}}=100\cdot \frac{2\cdot \mathrm{intersect}({\mathrm{DEGs}}_{\mathrm{Platform}\;1},{\mathrm{DEGs}}_{\mathrm{Platform}\;2})}{{\mathrm{DEGs}}_{\mathrm{Platform}\;1}+{\mathrm{DEGs}}_{\mathrm{Platform}\;2}}$$

where *intersect* is the number of genes with concordant changes.

When applied to 2 platforms measuring an identical set of genes’ mRNA expression this formula is identical to that of Shi et al [23], used to derive “POG”, or percentage of overlapping genes, from 2 lists of platform specific gene expression changes. The same formula can be used to derive cross-cohort performance (C_ccT_) in the same platform.

#### Gene categorization and network analyses

Genes exhibiting concordant changes were categorized by a subgroup of the authors (JAS, EMR, MRB) based on manual curation of literature and NCBI website information relevant to myocardium. Canonical pathways, biological functions and gene networks were generated through Ingenuity Pathway Analysis (IPA) [24] (https://www.qiagenbioinformatics.com/products/ingenuity-pathway-analysis). “Superheat” maps were generated by the Barter-Yu graphical tool using R [25]. Column and row ordering of heatmaps was determined by hierarchical cluster solution for LVEF change tertile 3 subjects.

#### Source data and protocol warehousing

Source data files from the 3 measurement platforms plus clinical data are warehoused in the GitHub repository (https://bristowlab.github.io/borg.html). Endomyocardial biopsy, RNA extraction and mRNA measurement protocols are available at https://www.protocols.io/view/borg-protocols-2uggetw/abstract.

## Results

### Cross-platform concordance

#### Candidate genes in the *A-S* cohort

Responder (R) group analysis. For the 50 candidate genes, in the *A-S* cohort we compared mRNA measurements determined by microarray and RT-qPCR (Table 1). In the change from baseline Responder analysis, 23 (46%) of the 50 candidate gene module exhibited changes by RT-qPCR, and 24 (48%) by microarray. For downregulated genes, in RT-qPCR there were 18 changes (P = 0.0009 vs. upregulated) and 14 in microarray. The C_cpT_ for all concordant changes in both platforms was 60% (Table 1). In the R analysis 33 (66%) of the 50 candidate genes exhibited changes by either RT-qPCR or microarray.

**Table 1:** Comparison of significantly different (P <0.05) candidate genes (of 50 total mRNAs measured) detected by RT-qPCR or microarray in the *A-S* cohort.

Directional Change	RT-qPCR Genes				Microarray Genes				Concordant Genes	
	R	Fold-change	R/NR	Fold-change	R	Fold-change	R/NR	Fold-change	R	R/NR
Upregulated	ADRB1	2.13	ADRB1	1.64	ADRB1	1.33	ADRB2	1.17	ADRB1	ADRB2
	PDK4	1.02	ADRB2	1.35	ADRB2	1.24	PFKM	1.08	PLN	PFKM
	PLN	1.41	ADRA1A	1.55	HNRNPD	1.06	PLN	1.11	THRA1	PLN
	TNNT2	1.07	PFKM	1.37	ADRA1A	1.21	RYR2	1.10		RYR2
	THRA1	1.50	PDHX	1.19	AGTR1	1.48	MYH6	1.35		MYH6
			CPT1B	1.56	PFKM	1.05	MYL3	1.05		MYL3
			ATP2A2	1.70	PLN	1.12				
			PLN	1.53	RYR2	1.10				
			RYR2	1.33	MYL3	1.03				
			MYH6	3.31	THRA1	1.10				
			MYL3	2.01						
N	5		11		10		6		3	6
Downregulated	GNAS	0.94	NPPA	0.61	GNAI2	0.94	SLC9A1	0.90	GNAI2	NPPA
	GNAI2	0.77	NPPB	0.65	SLC9A1	0.88	EDN1	0.79	SLC9A1	NPPB
	SLC9A1	0.63			EDN1	0.87	ACTA1	0.87	HK2	
	HK2	0.58			HK2	0.86	TNNI3	0.96	CASQ2	
	PDHX	0.74			CASQ2	0.93	DMD	0.93	SLC8A1	
	CASQ2	0.87			SLC8A1	0.91	NPPA	0.71	ACTA1	
	SLC8A1	0.67			CANX	0.96	NPPB	0.40	TNNI3	
	ACTA1	0.81			ACTA1	0.87			NPPA	
	ACTC1	0.83			TNNI3	0.96			NPPB	
	MYL2	0.83			DMD	0.91			CSRP3	
	TNNC1	0.98			NPPA	0.74			IL6	
	TNNI3	0.96			NPPB	0.52				
	NPPA	0.71			CSRP3	0.98				
	NPPB	0.66			IL6	0.90				
	CSRP3	0.76								
	CTF1	0.85								
	IL6	0.91								
	THRA2	0.82								
N	18	-	2	-	14	-	7	-	11	2
**Overall Total number and % Concordance (C**_**cpT**_**), both platforms, up and downregulated genes**									28/47	16/26
									(60%)	(62%)

R/NR analysis. For the R/NR analysis (Table 1) 18 (36%) of the 50 candidate genes exhibited changes by either RT-qPCR or microarray (vs. R, P = 0.059 for RT-qPCR, 0.023 for microarray, and 0.003 for both platforms). For upregulated genes RT-qPCR identified 11 and microarray 6 changes, all of which were concordant with RT-qPCR. For downregulated genes, RT-qPCR identified 2 (P = 0.007 vs. upregulated) changes that were both concordant with microarray measurements, which had 7 changes. The R/NR C_cpT_ was 62%.

#### Candidate gene expression measured by three platforms in the *S-R* cohort

In the *S-R* cohort all three platforms generated measurements, shown in Table 2. Only one change was concordant in all 3 platforms, marked downregulation (by >90%) in the natriuretic peptide NPPB, a biomarker of ventricular myocardial hypertrophy, in the R/NR analysis. The Na^+^/H^+^ antiporter SLC9A1 (NHE1), which is involved in mediation of β_1_-adrenergic signaling of hypertrophy [26], exhibited two platform concordance between RNA-Seq and RT-qPCR for R, and RNA-Seq and microarray for R/NR.

**Table 2:** Candidate genes in *S-R* cohort, mRNA expression fold changes from baseline within NR, R groups or comparison of R, NR fold changes (R/NR), changes with P <0.05.

Directional Change	Gene	RT-qPCR		Microarray		RNA-Seq	
		R Fold-change*	R/NR Fold-change^†^	R Fold-change*	R/NR Fold-change^†^	R Fold-change*	R/NR Fold-change^†^
Upregulated	ADRB1	**1.86**				**1.60**	1.47
	AGTR1	**1.66**				**1.81**	
	PLN	1.32					
	ATP2A2		**1.41**			1.39	**1.38**
	MYH6						2.74
	MYL3				**1.07**	1.51	**1.50**
	GNAQ				1.11		
	PFKM				1.10		
	RYR2				1.18		
	ADCY5					1.20	
	CPT1B					1.18	
	IL6ST						
	THRA						
	ADRB2						
	NPPB						
Totals	15^‡^	3	1	0	5	6	4
Upregulated	AGT	0.83					
	CASQ2	0.62	**0.57**				**0.73**
	DMD	0.81					
	GNAI2	0.73					
	HK2	0.31					
	IL6	0.44					
	NPPB	**0.05**	***0*.*07***		***0*.*07***	**0.078**	***0*.*06***
	PDHX	0.75					
	SLC8A1	0.63					
	SLC9A1	**0.46**			**0.77**	**0.64**	**0.56**
	MYL2						0.62
	TNNI3				0.94		
	PDHX					0.84	
	ADRBK1			0.93			
	GNAQ						
	PLN						
	RYR2						
Totals	17	10	2	1	3	3	4
Total up- or downregulated genes		13	3	1	8	9	8
		16		9		17	
P value for up, down-regulated genes in R + R/NR		0.15					
C_cpT_, ≥2 platforms^‡^		4/13)	3/3	0/1	3/8	4/9	5/8
		(31%)	(100%)	(0%)	(38%)	(44%)	(62%)
Cross-platform C_cpT_ P values		R, 0.02; R/NR, 0.18					

Cross-platform concordant (C_cpT_) fold change in 2 platforms is in **bold type**, in 3 platforms in ***bold type*, *italics)*.**

*Wilcoxon sign rank test for change from baseline

^†^Wilcoxon rank sum test comparing changes in NR to R^‡^≥platforms = concordance in comparison of any 2 platforms (doesn’t require 3 platform concordance).

The relatively small, N = 12 *S-R* cohort provided an opportunity to assess sensitivity of change detection among the 3 platforms. Of the 50 candidate genes, RT-qPCR, microarray and RNA-Seq yielded respectively 16, 9 and 17 statistically significant changes from baseline (R + R/NR analyses), P = 0.15 for change detection among platforms. The C_cpT_ value within specific platforms calculated as agreement with at least 1 other platform trended lower in microarray vs. respective values in RT-qPCR or RNA-Seq (in R 0% vs. 31% and 44%, P = 0.02; in R/NR 38% vs. 100% and 62%, P = 0.18).

#### Global gene expression in the *S-R* cohort, measured by microarray and RNA-Seq

Microarray and RNA-Seq platform measurements of global gene expression were used to investigate change concordance in the *S-R* cohort. Microarray detected 19,672 individual gene transcripts and 15,364 (78%) of these were also detected by RNA-Seq, forming the index number of measurements for analysis. S2 Table gives the alpha 0.05 list of up- or down-regulated genes within R and R/NR analyses, which are summarized in the denominators for change and concordance calculations at the bottom of Table 3.

**Table 3:** Cross-platform concordance of global gene expression changes, mRNA measurements by array and RNA-Seq in the *S-R* cohort, responder and R/NR datasets, changes at alpha 0.05.

Upregulated			Downregulated					
R	R/NR		R	R/NR				
BPHL	ADAM11	MYLK4	CXCL5	AATF	CYP19A1	MAP7	RRAS	ZMYND17
DHFRL1	ADCYAP1R1	NCR00201	EGLN3	ABAT	DCBLD2	MEG3	RSPH3	ZNRF2
FEZ1	ALDH2	NDUFB9	EIF2C1	ABCD1	DDA1	MELK	SDSL	42989
KANK1	AMD1	PDE7B	FUCA2	ABHD12	DHCR24	MEST	SERPINE2	42797
LDB2	AQP7	PEBP4	KCTD9	ACAD11	DLK2	MFI2	SETD3	42982
NUDT4	ART3	PHACTR3	KP1	ACE2	DOK4	MLLT11	SGK1	
PHACTR3	ASB10	PIM3	LUZP1	ACTN1	DSYN1	MOXD1	SGTA	
PKD1L1	ATP5I	PLA2G4F	PRKCA	ADC	E2F1	MPV17	SH2D4A	
PRELID2	BCL11A	PLAG1	RHBDD1	ALDH3A2	EIF4G3	MPV17L2	SH3GL2	
RG9MTD3	BCL6	PLCL2	TSPAN17	APLP1	ENC1	MTMR3	SH3GLB1	
SEC16B	C1orf105	POLR2I	USP9Y	APOA1	ENO3	MXRA5	SHB	
SLC26A9	C3orf43	PPP1R1A	VTI1B	APOL4	ENOX2	MYOZ3	SHC2	
SYCP3	C7orf70	PRELID2		ARHGAP11A	EXOC6B	NCAPH	SHROOM3	
TPSAB1	CACYBP	RAF1		ARSD	EXT1	NEB	SIRPA	
ZNF135	CD5L	RET		ARSE	EXTL3	NOTCH2NL	SLC1A4	
	CEL	RNF165		ASPM	FARP1	NPPB	SLC1A7	
	CHDH	RORC		ATP6V1E2	FN1	NPR3	SLC9A1	
	CKM	RPL24		ATRNL1	FOXM1	ODC1	SMAD6	
	COL28A1	SCN1A		BEX1	GDF11	OGDHL	SMOC2	
	COLEC12	SCN7A		BGN	GRN	ORMDL3	SNCA	
	CORIN	SDK1		C11orf24	HCCS	P1L4	SOCS2	
	CPA3	SEPP1		C1orf21	HELB	PACSIN1	SPINT2	
	DLK1	SHISA3		C5orf46	HERC5	PAPPA2	SPTAN1	
	DH11	SLC19A2		C7orf53	HEXB	PCSK5	SRPX2	
	FAM124A	SLC26A9		C9orf30	HLX	PDE1A	ST3GAL4	
	FAM179A	SLC27A6		CALU	HMGB3	PDE8B	STK17A	
	FGFBP2	SYCP3		CASQ1	HTRA1	PDLIM4	SVEP1	
	FNDC5	SYT3		CC2	INPP4B	PDPN	SVIL	
	GALNTL1	THAP1		CCNJL	IQGAP3	PENK	SYTL4	
	GRIN2A	TJP2		CDC25A	JAZF1	PGBD5	SYTL5	
	GZMK	TKTL1		CDH17	KCB1	PHYHIP	TBC1D22A	
	HADHB	TMEM132C		CDH2	KCNC4	PI16	TFDP2	
	HEY2	TPRKB		CDK8	KCNJ4	PIK3R2	TGOLN2	
	HIST3H2A	TPSAB1		CENPA	KCNJ5	PLCD4	THBS4	
	HMGCS2	UCKL1		CENPN	KIAA0556	PLCE1	TMED3	
	HSPB3	VRK2		CHD3	KIAA1244	PLIN3	TMEM51	
	IL6R	VWA3A		CHMP4B	KIAA1539	POLR1E	TNNT1	
	KAT2B	WDSUB1		CHMP4C	KIF4A	PORCN	TOP2A	
	KCNJ2	WNT5A		CHPF2	KLHL6	PROS1	TPD52L1	
	KIAA1328	ZBED5		CKAP4	LGALS9	PTPRH	TPM3	
	LARP4B	ZNF33B		CLTCL1	LGI2	QPCT	TPX2	
	LPAR3	ZNF585A		COL18A1	LNP1	QSOX1	TRIM41	
	LRRC39			COL1A1	LOXL1	RAB15	TSPAN5	
	MCF2			COL23A1	LOXL2	RAB31	TSTA3	
	MLPH			COL4A1	LTBP2	RAB6B	UBR1	
	MRO			CRELD1	LUZP1	RASL11B	UNC13C	
	MRPL43			CRLF1	LYPD1	RCAN1	VDR	
	MRPS24			CTGF	MAGED2	RELL1	WDR77	
	MTSS1			CUX1	MAP1A	RGS4	WHSC1	
	MYL3			CYP11A1	MAP2	RNF214	XPO4	
**(N)/total changes, Array or RNA-Seq**								
15/279 (Array)	92/869 (Array)		12/294 (Array)	205/936 (Array)				
15/631 (RNA-Seq	92/378 (RNA-Seq)		12/1093 (RNA-Seq)	205/1078 (RNA-Seq)				
			**Total Cross-Platform Concordance (C**_**cpT**_**), both platforms, upregulated and downregulated genes**					
−	−		**R: 54/2297 (C**_**cpT**_ **2.4%)**			**R/NR: 594/3401 (C**_**cpT**_ **17.5%)**		
			**(27 genes with changed expression in both microarray and RNA-Seq)**			**(297 genes with changed expression in both microarray and RNA-Seq)**		

R analysis. In the R analysis microarray detected 573 genes with mRNA changes, or 2.9% of the transcripts measured (279 upregulated and 294 downregulated, P = 0.53). RNA-Seq identified 1724 changes (11.2% of the transcripts measured, P <0.0001 vs. microarray), consisting of 631 upregulated and 1093 downregulated genes (P <0.0001). Concordance analysis between microarray and RNA-Seq (Table 3) yielded a total of 27 genes in the R analysis (15 upregulated and 12 downregulated), for a C_cpT_ for any gene expression change of 2.4%.

R/NR analysis. In the R/NR analysis microarray identified 1805 genes with changed expression (9.2% of total mRNAs measured, 869 upregulated and 936 downregulated, P = 0.11), while RNA-Seq detected 1456 changes (9.5% of total measured transcripts), of which 378 were upregulated and 1078 downregulated (P <0.0001). The R/NR dataset exhibited 297 changes that were concordant between microarray and RNA-Seq (92 upregulated and 205 downregulated (P <0.0001)) (Table 3), with a combined platform C_cpT_ of 17.5% (P <0.0001 vs. *A-S* R/NR (PCR/microarray) C_cpT_ of 62%). The within *S-R* C_cpT_ comparison between candidate (6/16, 38%) and global changes measured by microarray/RNA-Seq yielded a P value of 0.036. Table 4 contains a biologic categorization of genes with concordantly changed expression for the microarray/RNA-Seq R/NR analysis in the *S-R* cohort, with the category assignments for each gene given in S3 Table. For all 297 genes with cross-platform concordantly changed expression, the disproportionate downregulation by a factor of 2.2 fold compared to upregulation is due to changes in the Growth/Hypertrophy (P = 0.026, Hochberg adjusted P = 0.078), Cell Homeostasis (respective Ps 0.005, 0.027), Fibroblast Growth/extracellular matrix (Ps 0.001, 0.008), Extracellular to Intracellular Signaling (Ps 0.022, 0.077), Cytoskeleton (Ps 0.041, 0.10), and Unclassified/unknown Function (Ps = 0.002, 0.016) categories. S3 Table is a more detailed biologic categorization of genes with concordant expression changes in *S-R*, to which 2 additional candidate genes that were concordantly changed by RT-qPCR and RNA-Seq (upregulation in ATP2A2 and downregulation in CASQ2, Table 2) have been added.

**Table 4:** Biologic categories of concordant global gene expression changes measured by microarray and RNA-Seq in the *S-R* cohort, R/NR analysis (from Table 3).

Biologic Category	Up-regulated	Down-regulated	P value*	P-value, Adjusted^†^
Contractile and associated proteins	3	2	1.00	1.00
Metabolism	8	9	1.00	1.00
Growth/hypertrophy Regulation	8	21	0.026	0.078
Channels, Solute Exchangers, Transporters	8	7	1.00	1.00
Ca2+ Handling or signaling	0	1	1.00	1.00
Cell Homeostasis (Golgi, ER, membrane and cytosol trafficking)	8	25	0.005	0.027
Fibroblast growth, Extracellular Matrix or TGF signaling	3	21	0.001	0.008
Extracellular to intracellular signaling^‡^	16	33	0.022	0.077
Apoptosis	2	8	0.11	0.24
Microtubules	0	3	0.25	0.41
Immune function other than cytokines	5	2	0.45	0.68
Vascular/thrombosis	1	3	0.62	0.84
Cytoskeleton	0	6	0.041	0.10
Gene regulation: Transcription factors, RNA binding, nucleotide processing, translation, nucleosome components, noncoding RNAs	14	24	0.14	0.27
Unclassified/Unknown Function In the heart	16	40	0.002	0.016
**Totals**	92	205	P <0.0001	

*Chi-square Test for differences in proportions.

†P-value adjustment via Benjamini-Hochberg.

‡Includes neurohormonal signaling, PI3K, PLC, Small GTPases and regulators, cytokines, and other signaling pathways.

RNA-Seq detected more genes with changed expression in R than in R/NR (1724 vs. 1456, p <0.0001), while microarray identified more changes in R/NR than R (1805 vs. 573, p <0.0001). C_cpT_ was 6.2 fold higher in R/NR compared to R (Table 3). The C_cpT_ of 2.4% in R for global gene expression was matched by a 0% (no concordance in 10 changes) for microarray/RNA-Seq in *S-R* (Table 2). For R/NR C_cpT_ was higher, 17.5% for global (Table 3, P <0.0001 vs. R) and 38% (6/16) for candidate gene microarray/RNA-Seq (Table 2, P = 0.053 vs. R, 0.036 vs. global R/NR).

### Cross-cohort comparisons

In order to derive direct information on whether smaller cohorts of hyper-responders can substitute for larger cohorts comprised of smaller degrees of phenotypic response in longitudinal reverse remodeling gene expression studies, we compared measurements between the *A-S* and *S-R* cohorts using data from RT-qPCR for candidate genes and microarray for global gene expression.

#### Cross-cohort concordance for candidate gene expression changes in *A-S* vs. *S-R* assessed by RT-qPCR

R analysis. Genes with p <0.05 changes in expression for RT-qPCR measurements, the protocol primary method for candidate gene mRNA change detection [8], are given for the 2 cohorts in Table 5. In the *A-S* cohort R analysis, 23 genes exhibited expression changes (5 upregulated and 18 downregulated, P = 0.002), while 13 changes were identified in the *S-R* cohort (3 upregulated, 10 downregulated, P = 0.037) (P = 0.059 vs. total changes in *A-S*). Thus, in the R analysis RT-qPCR identified more down-regulated genes in both the *A-S* and S-R cohorts. The two cohorts were concordant for 2 upregulated (ADRB1 and PLN) and 8 downregulated (GNAI2, SLC9A1, AGT, HK2, PDHX, CASQ2, SLC8A1, DMD, NPPB and IL6*)* genes (Table 5, P = 0.046). Within the R group the 10 concordantly changed genes in the *A-S* and *S-R* cohorts were from a total of 35 upregulated and downregulated genes, for a C_ccT_ of 56% (Table 5).

**Table 5:** Cross-cohort candidate gene concordance of RT-qPCR in the *A-S* vs. *S-R* cohorts (changes of P < 0.05).

Directional Change	RT-qPCR *S-R* Genes				RT-qPCR *A-S* Genes				Concordant Genes	
	R	Fold-change	R/NR	Fold-change	R	Fold-change	R/NR	Fold-change	R	R/NR
Upregulated	ADRB1	1.86	ATP2A2	1.72	ADRB1	2.13	ADRB1	1.64	ADRB1	ATP2A2
	AGTR1	1.66			PDK4	1.02	ADRB2	1.35	PLN	
	PLN	1.32			PLN	1.41	ADRA1A	1.55		
					TNNT2	1.07	PFKM	1.37		
					THRA1		PDHX	1.19		
							CPT1B	1.56		
							ATP2A2	1.70		
							PLN	1.53		
							RYR2	1.33		
							MYH6	3.31		
							MYL3	2.01		
N	3		1		5		11		2	1
Downregulated	GNAI2	0.73	CASQ2	0.57	GNAS	0.94	NPPA	0.61	GNAI2	NPPB
	SLC9A1	0.46	NPPB	0.07	GNAI2	0.77	NPPB	0.65	SLC9A1	
	AGT	0.83			SLC9A1	0.63			HK2	
	HK2	0.31			HK2	0.58			PDHX	
	PDHX	0.75			PDHX	0.74			CASQ2	
	CASQ2	0.62			CASQ2	0.87			SLC8A1	
	SLC8A1	0.63			SLC8A1	0.67			NPPB	
	DMD	0.81			ACTA1	0.81			IL6	
	NPPB	0.05			ACTC1	0.83				
	IL6	0.44			MYL2	0.83				
					TNNC1	0.98				
					TNNI3	0.96				
					NPPA	0.71				
					NPPB	0.66				
					CSRP3	0.76				
					CTF1	0.85				
					IL6	0.91				
					THRA	0.82				
N	10	-	2	-	18	-	2	-	8	1
	**Total Cross-Cohort Concordance (C**_**ccT**_**), up, downregulated genes**								**20/36**	**4/16**
									**56%**	**25%**

R/NR analysis. Within the R/NR dataset of the *A-S* cohort 11 genes were upregulated while 2 genes were downregulated (Table 5, P = 0.007) (Table 5). In the *S-R* cohort 1 gene was upregulated while 2 were downregulated (total changes of 3 vs. 13 in *A-S*, P = 0.006). The 2 cohorts were concordant for 1 upregulated (ATP2A2) and 1 downregulated gene (NPPB), for a C_ccT_ of 25% (Table 5; P = 0.033 vs. R Group analysis).

Cross-platform comparisons between *S-R* and *A-S* cohorts. For candidate genes cross-platform concordance was compared between the *S-R* and *A-S* cohorts, using the 2 platform PCR/microarray comparison. Compared to the *A-S* cohort (Table 1), for the R analysis PCR/microarray C_cpT_ in *S-R* (Table 2) was 0/14 = 0% (P = 0.01 vs. C_cpT_ of 60% in *A-S*), and 2/11 = 18% for R/NR (P = 0.016 vs. C_cpT_ of 62% in *A-S*). Compared to PCR/microarray the *S-R* PCR/RNA-Seq C_cpT_ values were higher, 8/22 (36%) (P = 0.03 vs. *S-R* PCR/microarray) in R and 6/11 (55%, P = 0.08) in R/NR (Table 2). The *S-R* R and R/NR C_cpT_ values for microarray/RNA-Seq were respectively 0/10 (P = 0.07 vs. PCR/RNA-Seq) and 6/16 = 38% (P = 0.38 vs. PCR/RNA-Seq) (Table 2). Thus, candidate gene cross-platform concordance for PCR/microarray was not as good in *S-R* as compared to the *A-S* cohort, but the higher 2 platform concordances of PCR/RNA-Seq vs. microarray containing C_cpT_ combinations suggests part of the issue was microarray platform related.

#### Cross-cohort concordance for global gene expression changes as assessed by microarray

R Analysis. The number of significantly different global genes based on microarray measurements in the *S-R* cohort is given in S2 Table, and for the *A-S* cohort in S4 Table. The R group analysis yielded a total of 2931 upregulated genes in the *A-S* cohort (represented in the denominator of total changes in S5 Table, while 2294 upregulated genes were detected in the *S-R* cohort (p <0.0001). A total of 3274 downregulated genes were identified in the *A-S* cohort while 2095 genes were detected in the *S-R* cohort (p <0.0001). There were only 60 upregulated and 42 downregulated concordant changes, for a C_ccT_ of 1.9% (S5 Table).

R/NR analysis. In the R/NR patient group microarray detected 850 upregulated genes in the *A-S* cohort (S4 Table), while 869 were detected in the *S-R* cohort (S2 Table). Downregulated genes were n = 1085 in the *A-S* cohort (S4 Table), and 936 in the *S-R* cohort (S2 Table, P = 0.0006 vs. *A-S*). In marked contrast to the R analysis, the R/NR analysis had much higher concordance in both up- (N = 391) and down- (N = 393) regulated genes, with a C_ccT_ of 42% (P <0.0001 vs. R analysis) (S5 Table).

### Platform concordance of gene expression changes has biologic significance

**Fig 2:**
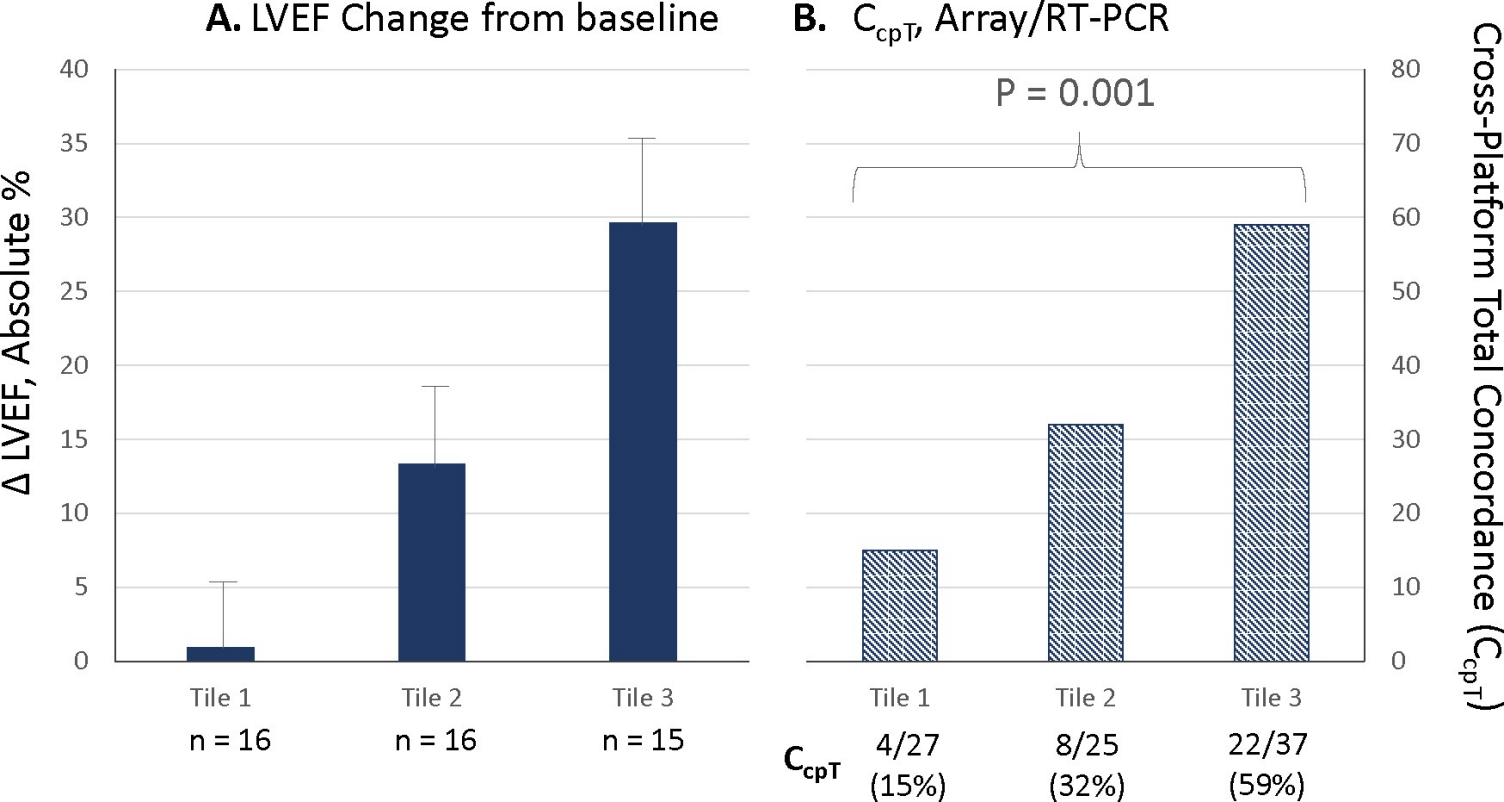
LVEF change and C_cpT_ by tertile. **(A)** LVEF change by tertile, mean ± SD in absolute %; (**B**) Gene expression change from baseline at P <0.05, Overall Cross-platform Total % Concordance (C_cpT_) by tile. LVEF baseline → LOCF* values (mean in % ± SD): Tile 1, 27.8±10.3 → 28.7±10.3; Tile 2, 27.6±8.4 → 40.9±10.5; Tile 3, 23.6±7.7 → 53.1±6.7. ANOVA P values: baseline = 0.32, LOCF <0.0001.

The *A-S* cohort, which had LVEF changes ranging from -8 to 47.5 absolute %, created the opportunity to examine the relationship between reverse remodeling as a biologic effect and cross-platform concordance of gene expression changes. For changes from baseline, candidate gene microarray and RT-qPCR C_cpT_ values were related to LVEF change tertiles (Fig 2, S6 Table). The first tile of LVEF change (Fig 2) corresponds to the NR group (LVEF change in absolute %±SD = 0.9±4.4%), while the 2^nd^ and 3^rd^ tiles consist of R group subjects that have respective LVEF increases of 13.3±4.0 and 29.6±6.1 absolute %. There were no differences in baseline LVEF values, which ranged from 27.8% in tile 1 to 23.6% in tile 3 (ANOVA P = 0.32). The tiles 2 and 3 LOCF LVEFs were respectively 40.9% and 53.1% (Fig 2), indicating phenotypic improvements into the HFmrEF (HF with “mid-range” LVEF) and normal ranges. The tile 1 LVEF remained in the lower range of HFrEF (P <0.0001 across the 3 LOCF tiles).

By RT-qPCR there were respectively 25, 12 and 20 up or downregulation changes progressing up the tertile (P = 0.026) (Fig 2). Microarray measurements yielded 2, 13 and 17 respective changes (P = 0.008) by tertile. For both platforms the number of changed genes by tertile was 27, 25 and 37 (P = 0.033) progressing from Tiles 1 to 3, but tile 1 was not significantly different from tile 3 (P = 0.083) and there is no obvious reverse remodeling vs. gene expression change relationship, with tile 2 having numerically fewer changes than tiles 1 or 3. However, RT-qPCR and microarray cross-platform concordance exhibited a direct relationship to reverse remodeling across the LVEF tiles, with respective C_cpT_ values of 15%, 32% and 59% (overall P < 0.001) (Fig 2, S6 Table). Thus, in a set of candidate genes enriched for having a possible relationship to LV remodeling, concordance of gene expression changes increases directly and substantially with the degree of reverse remodeling.

### Gene network/signaling pathway analyses

#### Pathway analyses of concordant R/NR changes

**Canonical pathways.** To investigate the possible canonical signaling pathways involved in the concordance related biological effect on LV remodeling, for microarray/RNA-seq concordant changes in global gene expression in *S-R* we conducted an Ingenuity Pathway Analysis (IPA) [24] on the 299 R/NR concordant genes listed in S3 Table with the results shown in S7 Table. The methodological background for the IPA analyses is given in the Supporting Information. S3 Table indicates there were 92 upregulated and 207 downregulated genes based on 297 concordantly changed genes in Table 3 and an additional 2 in Table 2. Of the most negative z scores in S7 Table (values < -1.5), evidence for a net pathway inhibition, 3 of them were phosphoinositide related pathways containing many of the same molecules (Superpathway of Inositol Phosphate compounds, 3-phosphonositide Degradation and D-myo-inositol-5-phosphate Metabolism), belonging to the biologic categories (Table 4, S3 Table) of Growth/hypertrophy regulation (PTPRH, CDC25A), extracellular to intracellular signaling (PLCE1, PLCD4 (subcategory phospholipase C signaling), PPP1R1A (subcategory neurohormonal signaling); PIK3R2, INPP4B, MTMR3 (subcategory PI3 kinase signaling); and SIRPA (subcategory other signaling)). The other 2 pathways identified with z scores < -1.5, GP6 Signaling and Dendritic Cell Maturation contained collagen and phospholipase C or PI3 kinase signaling molecules. GP6 is a collagen receptor expressed in platelets, but the IPA is likely detecting extracellular matrix collagen transcript downregulation.

Only 6 pathways had positive z scores (indicating net activation), with the highest, calcium signaling, considered statistically significant with a z-score of 2.00 and an overlapping P value of < 0.01. Cardiac β-adrenergic signaling and protein kinase A signaling also had positive z-scores, in a clinical setting of high dose β_1_ adrenergic receptor blockade. Downstream of β_1_-adrenergic signal transduction is calcium signaling and the initiation and regulation of contractile function [27]. The 8 mRNA gene products grouped together within the calcium signaling Ingenuity canonical pathway in S7 Table are involved in calcium binding/uptake or release (GRIN2A, ATP2A2, CASQ1, CASQ2), or are directly or indirectly regulated or activated by calcium (RCAN1, MYL3, TPM3, TNNT1). In S3 Table they are members of the channels/solute exchanger/transporters (GRIN2A), Ca^2+^ handling or signaling (ATP2A2, CASQ1, CASQ2), contractile and associated proteins (MYL3, TPM3, TNNT1) or growth/hypertrophy regulation (RCAN1).

The second most positive z-score in the IPA was for Gα12/13 signaling (S7 Table), which has not been previously identified as having a molecular signature in the reverse remodeled failing human heart. GNA12 and GNA13 are closely related members of a class of pertussis-toxin insensitive G-proteins [28] that transduce signaling in lysophosphatidic acid (LPA), angiotensin II, endothelin-1, MAP kinase, PI3 kinase and other pathways involved in cardiac myocyte hypertrophy [28–31]. The Gα12/13 signaling module contained an upregulated LPA receptor (LPAR3), an upregulated MAP kinase kinase (RAF1), 2 downregulated cadherin genes (CDH2 and CDH17), a downregulated PI3 kinase regulatory subunit (PIK3R2) and an upregulated contractile protein gene, MYL3, the “essential” ventricular myosin light chain that is considered to be an “adult” expressed gene within the fetal-adult gene expression paradigm.

#### Noncanonical disease associations

**Fig 3:**
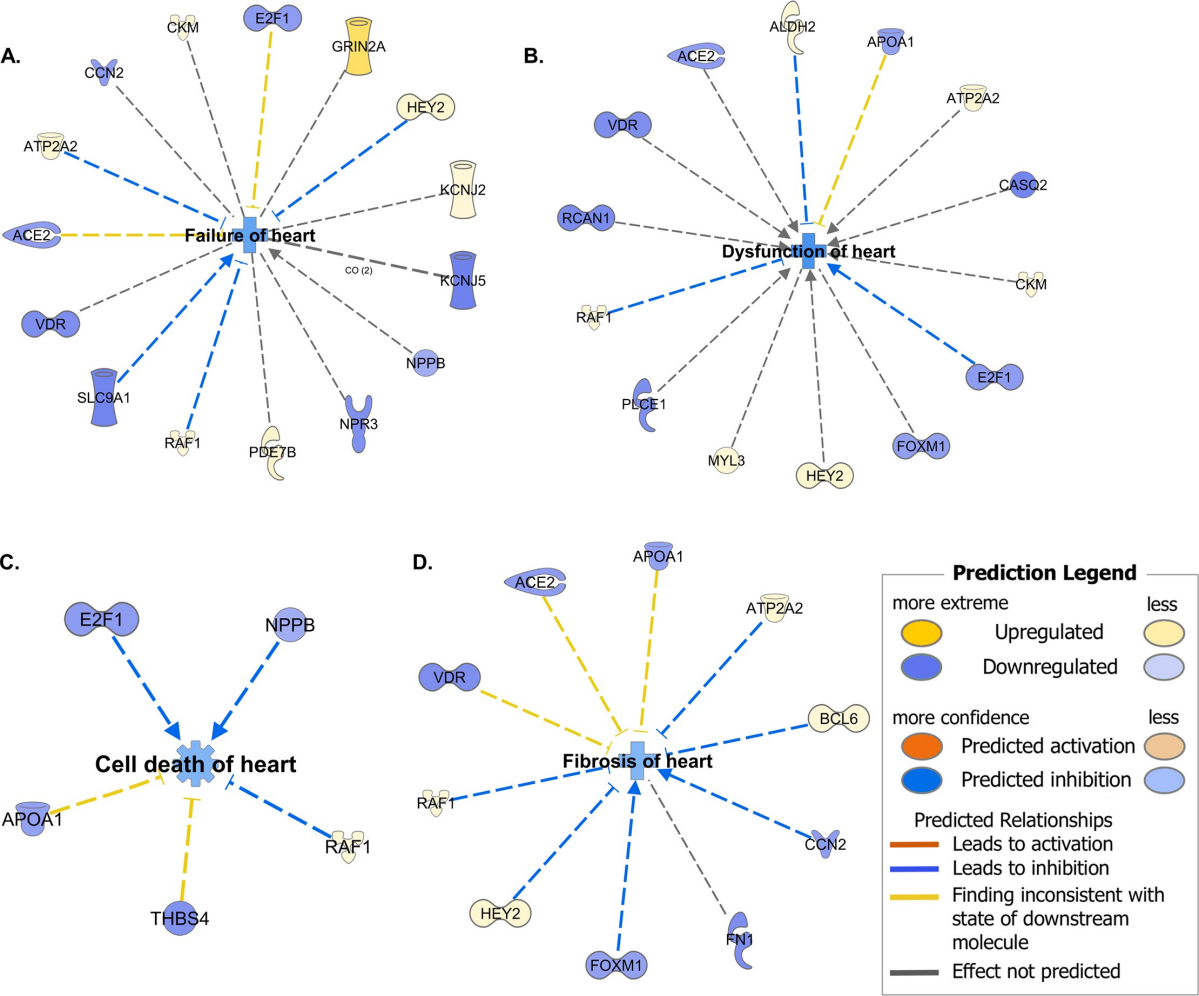
Ingenuity pathway analysis of concordantly changed mRNAs from Table 3. Colors indicate predicted effect of transcript on biological function or disease in center of circular diagram. (**A**) Dysfunction of heart. (**B**) Failure of heart. (**C**) Cell death of heart. (**D**) Dilated cardiomyopathy.

Fig 3 is the “tox function” IPA analysis for 4 domains relevant to ventricular chamber remodeling. Failure (A), Dysfunction (B), Cell Death (C), and Fibrosis (D) of the heart are all predicted to be inhibited by the noncanonical gene expression changes in the *S-R* cohort (Tables 2 and 3).

### Correlation of candidate with global gene expression changes in *A-S* cohort

**Fig 4:**
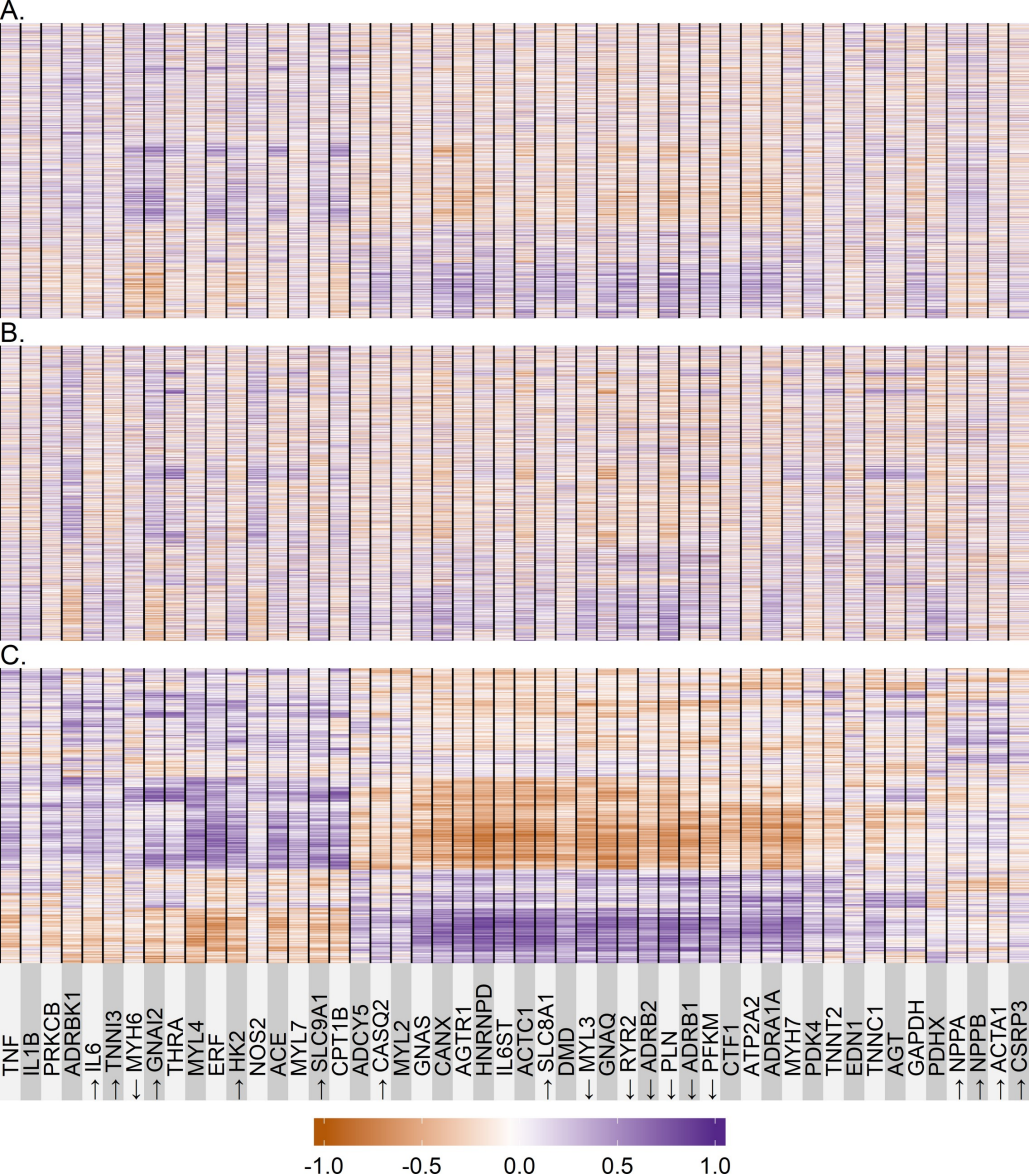
Superheat maps. **(A)** Spearman’s Rho of baseline vs. LOCF mRNA abundance, superheat map of 19,672 separate mRNA gene products (rows along Y-axis) vs. the 50 candidate gene mRNAs, patients in tile 1 of LVEF change (Fig 2). Arrows indicate candidate genes with concordant changes measured by RT-qPCR and microarray in either the R (Responder) or R/NR (Responder/Nonresponder) analysis; **(B)** Same as Fig 4A., except data are from tile 2 in Fig 2; **(C)** Same as Fig 4A., except data are from tile 3 in Fig 2.

In the *A-S* cohort we used the “Superheat” [25] graphical package to assess the extent of microarray-measured candidate gene expression changes compared to those in the 19,672 global genes (Fig 4), within the LVEF change tertiles shown in Fig 2. In Fig 4 the 19 genes that had concordant R or R/NR changes (Table 1) are designated by arrows, and the 19,672 gene transcripts are displayed in individual horizontal rows that cannot be distinguished in the resolution. The heat map is arranged by degrees of correlated fold change ranging from a Rho of +1.0 (two genes, a candidate and a global) with log2 fold changes perfectly positively correlated to -1.0 (2 genes perfectly negatively correlated) in the 47 individual patients. The candidate genes along the x-axis are arranged by degree of correlation with the 19,672 global genes identified on microarray in tile 3 (Fig 4C); closer spatial proximity of candidate gene columns suggests similar patterns of correlation across non-candidate genes. In the 3^rd^ tile of LVEF change (Fig 4C, mean LVEF increase 29±6 absolute %) clear patterns of moderately positive or negative correlations are evident, which are not present in tiles 1 and 2 (Fig 4A and 4B; respective mean LVEF changes of 0.9±4 and 13±4 absolute %). In tile 3 (Fig 4C) there are 2 sets of candidate genes associated with reciprocal regulation of large numbers of global genes, which also contain a high percentage of concordant genes. Along the horizontal axis are candidate genes, ranging from GNAS to MYH7, which are positively (bottom part of the heat map) or negatively (middle-upper part) correlated of with large numbers of global genes’ expression. Of the 19 candidate genes in this region, 7 are concordantly changed, with 6 being upregulated including both ADRB1 and ADRB2. The positive and negative portions of the map encompass approximately 28% and 30% of the 19,672 genes displayed. The 19 candidate genes in this “hot” region (“Group 1”) have in common either being regulated by β_1_-AR signaling (n = 12; AGTR1, HNRNPD, MYL3, RYR2, ADRB2, PLN, ADRB2, PFKM, CTF1, ATP2A2, ADRA1A, MYH7), being involved in adrenergic receptor signal transduction (GNAS, GNAQ), being involved in Ca^2+^ handling or regulation (ATP2A2, SLC8A1, PLN, CANX), or being important in sarcomere structure (DMD, CTF1).

Beginning with TNF and continuing through SLC9A1 on the Fig 4C heat map are 17 candidate genes (“Group 2”) which, compared to the previous 19 candidate genes, reciprocally correlate with global genes (negatively correlated in the bottom part of the map and positively in the mid-upper region). Eight of these 17 were concordant, with 6 downregulated. This gene cluster contains 3 cytokines (TNF, IL1B and IL6), the thyroid hormone receptor THRA1, and several β_1_-adrenergically regulated genes (ADRBK1, MYH6, GNAI2, MYL4, HK2, CPT-1 and SLC9A1). Finally, the third region on the map beginning with PDK4 and proceeding through a total of 11 genes does not contain any obvious clustering of correlated mRNAs, similar to a 3 gene (ADCY5, CASQ2, MYL2) transition area between the two other regions. Of this total of 14 genes without correlative changes in expression, 4 (and CSRP3) were concordant, all downregulated. NPPA, NPPB, and ACTA1 are regulated by β_1_ adrenergic signaling. The other 7 members of this group consist of 3 metabolic genes (the β_1_-adrenergically regulated PDK4, PDHX and the reference gene GAPDH, 2 contractile proteins (TNNT2, TNNC1), and 2 neurohormonal genes (EDN1, AGT)).

## Discussion

We used cross-platform concordance of gene expression changes as a method of increasing the reliability of detecting biologically meaningful molecular changes associated with reverse remodeling in an idiopathic dilated cardiomyopathy study population. Cross-platform concordance has been widely used as a method to validate measurement methodologies [17–19,22,23], but in model systems it is also correlated with biologic and/or therapeutic signals [22,32–34]. The use of cross-platform concordance was validated through a highly statistically significant relationship of concordant candidate gene changes with progressive reverse remodeling. We then extended the use of concordant gene expression changes to pathway analyses, which revealed both expected and unexpected results.

### Cross-platform concordance of changes in gene expression

C_cpT_ values, a standard measurement of expression change concordance, were in the 60% range for RT-qPCR and microarray measurements of candidate genes in the 47 patient *A-S* cohort, for both R and R/NR analyses. For the smaller (n = 12) *S-R* cohort that exhibited a greater degree of reverse remodeling, C_cpT_ values were somewhat lower, particularly in the R analysis on 2 platform comparisons that included microarray data. The lower C_cpT_ values in the *S-R* cohort likely reflect the difference in statistical power between the larger and smaller cohorts, plus possibly somewhat lesser accuracy of transcript detection by microarray compared to RT-qPCR or RNA-Seq.

In contrast to candidate genes in the *A-S* cohort, concordance of changes for global genes as assessed by microarray and RNA-Seq in the *S-R* cohort were extremely low in the change from baseline R analysis, with a C_cpT_ of 2.4%. However, the microarray/RNA-Seq C_cpT_ for candidate genes in the S-R cohort was 0%, suggesting that the smaller sample size *S-R* cohort was ill-suited for the R analysis. The R/NR analysis C_cpT_ for global gene expression was much higher, 17.5%, but lower than the counterpart C_cpT_ microarray/RNA-Seq value for candidate genes (38%, P <0.036). These data emphasize the value of comparing R vs. NR changes (R/NR analysis), with the implication that an *in situ* myocardial gene expression study needs to be large enough to generate both reverse remodeling responders and nonresponders. In addition, the data support an expectedly higher concordance for candidate genes, which are enriched for genes likely to change expression in reverse remodeling. The larger global gene C_cpT_ for R/NR vs. R also supports the use of the R/NR analysis in pathway generation.

### Cross-cohort concordance of changes in gene expression

Cross-cohort concordance, C_ccT_, was measured in data generated by a single platform in a manner analogous to inter-platform concordance. For candidate gene mRNAs measured by RT-qPCR the *A-S* cohort tended to have (p = 0.059 in the R analysis) or had (p <0.01 in the R/NR analysis) more concordant changes, again indicating that the larger, *A-S* cohort had a statistical advantage that was not compensated by the greater degree of reverse remodeling in *S-R*. However, the *A-S*/*S-R* C_ccT_ of 56% in R and 25% in R/NR (Table 5) supports comparability of the *S-R* and *A-S* cohorts, not surprising since *S-R* is a subset of *A-S*.

In *A-S* and *S-R* changes in expression of global genes was assessed by microarray. In the R analysis the *A-S* cohort yielded proportionally more statistically significant changes than in *S-R* (by 41%), whereas in the R/NR analysis the number of changes was nearly equal in the 2 cohorts, with fewer than half the number of changes in both *A-S* and *S-R*. The C_ccT_ for the R analysis was extremely low, 1.9%. In contrast, the C_ccT_ for R/NR was markedly higher, 42%, and within the range for R or R/NR C_ccT_ for candidate genes. The basis for the marked difference in C_ccT_ values was an R/NR vs. R increase in concordant genes by 7.7 fold and a decrease in the number of either-cohort changed genes by 2.8 fold, with corresponding implied reductions in both Type 2 and Type 1 error in the R/NR analysis. These data provide support for the validity of the *S-R* cohort as representative of global gene expression changes in *A-S*, but only for an R/NR analysis.

### Biologic significance of platform concordance for gene expression changes

In the *A-S* cohort an analysis of platform concordance of changes in gene expression by degree of LVEF change indicated that most of the changes from baseline in the 1^st^ LVEF tertile, comprised entirely of Nonresponders, were non-concordant, and therefore likely to be false positives. In contrast, in patients undergoing reverse remodeling (tertiles 2 and 3) concordance was directly related to the degree of LVEF improvement and reached 59% in tile 3, where the increase in LVEF was by 29.6 absolute %. Candidate gene change analysis in the *S-R* cohort also provided support for the biologic significance of concordance. Two platform C_cpT_ exhibited a strong trend for an increase from NR to R to R/NR, reaching a value in R/NR (58%) that was quite similar to the *A-S* R/NR value of 62% despite the lower power of the *S-R* cohort. A straightforward interpretation of these data is that when comparing organ phenotypes, platform concordance for gene expression measurements is directly related to the degree of phenotypic change; that is, concordant changes have biologic meaning.

### Gene network/signaling pathway analyses for concordant *S-R* gene expression changes in microarray and RNA-Seq

The demonstration of an acceptable degree of platform and cohort concordance for the R/NR analysis in *S-R* created the opportunity to perform a network analysis, for purposes of hypothesis generation. In this dataset downregulation exceeded upregulation, and the biologic categories of gene changes were unsurprisingly led by the growth/hypertrophy, cell homeostasis, fibroblast/extracellular matrix and extracellular to intracellular signaling categories. These changes are all consistent with reverse remodeling with inactivation of genes involved in myocyte and interstitial cell growth.

Despite all study subjects receiving high doses of β-blocking agents, cardiac β-adrenergic signaling and protein kinase A signaling had positive z-scores. All study subjects were receiving target/guideline recommended doses of metoprolol succinate or carvedilol that produce high levels of blockade of β_1_-adrenergic receptors, and both agents have been shown to reverse adrenergically-mediated desensitization changes in failing hearts [35,36]. Although the low mRNA abundance ADRB1 gene was not concordantly upregulated in the R/NR global gene expression analysis in *S-R* (Table 3), it was by RT-qPCR in the R/NR analysis in *A-S* (Table 1), by PCR or RNA-Seq R analysis in *S-R* (Table 2), and by RNA-Seq in the R/NR analysis in *S-R* (Table 2). ADRB2 was concordantly upregulated in *A-S* R/NR (Table 1). Restoration of β-receptor signaling could have produced a net activation of downstream cAMP/PKA and calcium signaling via receptor constitutive activity or by adrenergic signaling intermittently overriding β-blockade. The cAMP/PKA canonical pathway contained 3 upregulated and 5 downregulated genes whose protein products have been shown to regulate cAMP levels or be modifiable by PKA phosphorylation. The canonical calcium signaling pathway featured upregulation of ATP2A2 (SR Ca^2+^ ATPase) downregulation of CASQ1 and CASQ2, the respective minor and major abundance calsequestrins expressed in the heart, and the calcium regulated contractile proteins MYL3, TNNT1 and TPM3. However, expression changes of other modulators of Ca^2+^ signaling in the heart were not included in the Ingenuity cluster, such as upregulation of the phosphatase 1 inhibitor PPP1R1A that would be expected to promote SR Ca^2+^ uptake and release by inhibiting phospholamban dephosphorylation. The activation or improvement in calcium signaling as the LV reverse remodels is supported by work in model systems [37].

There were also some interesting findings within the IPA canonical pathway analysis involving noncandidate genes that have not been previously reported in ventricular reverse remodeling in the human heart. Phosphoinositide signaling occupied 3 of the top 4 negative z score or inhibited pathways, 2 pathways containing numerous collagen transcripts were in the first 5 inhibited pathways, and Gα12/13 signaling was the second highest ranked of only 6 activated pathways. None of these pathways had z-scores with absolute values of >2.0, so the findings are only hypothesis generating. Neither phosphoinositide or Gα12/13 signaling have been prominently identified in our [38] or other large scale [39] human myocardial remodeling gene expression studies, but the role of phosphoinositide signaling in pathologic hypertrophy and heart failure have been reported in numerous animal model studies [40–45]. Importantly, the PI3 kinase signaling pathway interacts with β_1_-adrenergic receptor expression and signaling [46], providing biologic plausibility for phosphoinositide pathway gene expression changes in that in adult rat cardiac myocytes β_1_-adrenergic receptor signaling leads to increased PI3K activity which then attenuates adrenergically mediated positive inotropic effects [46]. Similarly, β_1_-adrenergic receptor signaling has been shown to be a powerful mediator of fibrosis and other forms of extracellular matrix deposition in model systems [40,47–50], but generally has not been considered a major therapeutic target of β-blockers in reverse remodeling. In the human heart [51] Gα12/13 transduces hypertrophic signals [52] for a number of non-β-adrenergic receptors such as endothelin-ET_A_ and angiotensin II AT_1_, and Gα12 mRNA expression is upregulated in the transition of pathologic hypertrophy to failure in the Dahl salt-sensitive rat model [52]. Despite this evidence that upregulation of Gα12/13 signal transduction can produce pathologic hypertrophy, several lines of evidence indicate that this signaling module may be cardioprotective [53,54], and the contractile protein member (MYL3) is an "adult" gene within the fetal/adult expression paradigm [55] that regulates contractility [56].

Of note is that the IPA canonical pathway Cardiac Hypertrophy Signaling had a neutral activation z-score, with an overlapping P value of–log 1.82 (0.015) (S7 Table), despite the biologic category of Growth/hypertrophy regulation having 21 downregulated vs. 8 upregulated genes (S3 Table). This discrepancy is likely due to differences in how the two classification instruments are constructed; the biologic category (S3 Table) placed genes with demonstrated changes into groupings based on curation of the current myocardium relevant literature and NCBI websites, while the IPA inserted the same changes into a proprietary algorithm where genes are assigned to signaling modules.

### Correlation of candidate with global gene changes in the *A-S* cohort

In a correlation analysis between changes in candidate and global gene expression in the *A-S* cohort two potential patterns were identified that may be candidates for extensive gene regulation networks. One group (Group 1) had in common β_1_-AR gene signaling, for which there is considerable previous evidence [8,57]. Group 2 included GNAI2, which regulates multiple signaling pathways involved in hypertrophy including LPA signaling [29,30]. Both these potential networks, which exhibited reciprocal regulation, appeared to contain an extensive number of genes likely numbering in at least the hundreds based on the size of the area on the heat map and assuming that only a small percentage (≤10%) of genes is imparting the color to the map. A third group of genes with no strong clustered correlations was also identified, with the natriuretic peptides NPPA/NPPB as the centerpiece.

### Basis for the advantage of cross-platform concordance in human *in situ* studies of gene expression

We evaluated gene expression change concordance as a means to improve the likelihood of biologic relevance of differential gene expression associated with reverse remodeling of failing, eccentrically hypertrophied human LVs. In terms of failure to achieve change concordance, both technical/methodological and biological causes are possible. For cross-platform concordance in the *A-S* cohort the majority of the non-concordance was presumably technical/methodological related, most likely related platform sensitivity and specificity of transcript identification and quantitation. However, endomyocardial biopsy sampling and RNA extraction variability would also fall within this category. For biologic sources of non-concordance, within the *A-S* R and NR groups (i.e. the components of the R/NR analysis) variance in degrees of remodeling and the relationship between remodeling and change in individual patient gene expression are also potential sources. For the much smaller sample size *S-R* cohort, both types of causes would be accentuated, and for the cross-cohort comparisons biologic causes would be a relatively greater contributor to nonconcordance since the responder phenotypes were quantitatively different.

All 3 methodologies used for cross-platform measurements have been extensively validated against each other [11–13,22,23], and with the possible exception of microarray in the R analysis in the *S-R* cohort, they performed similarly in this study. Compared to RT-qPCR and RNA-Seq microarray may have been at some disadvantage for low abundance mRNAs, but nevertheless was able to detect changes in some of them such as β-adrenergic receptors. TaqMan or Real Time qPCR has long been considered the “gold standard” for RNA quantitation but is impractical for measurement of global gene expression. Microarrays revolutionized the measurement of genome-wide transcriptomics, and held sway as the dominant methodology for over a decade [58]. Due to its flexibility for measuring multiple RNA gene products and now being cost-effective, RNA-Seq has replaced microarrays as the most widely used methodology for identifying and quantifying multiple RNA species [59].

As the methods for quantifying mRNA abundance are different for the 3 technologies, the statistical probability of achieving a P <0.05 with the same directional change across 2 platforms is 0.05^2^/2 or 0.00125. Therefore, some of the value of cross-platform concordance can be attributed to statistical stringency. However, the utility of a 2^nd^, confirming measurement in real time by different methodology likely confers value for concordance beyond setting a lower P value to minimize false discovery rate (FDR). In gene expression experiments that include large numbers of measurements problems with controlling FDR without having an adverse effect on sensitivity are well known [60,61], and contemporaneous measures of cross-platform concordance can be viewed as an alternative to using stringent P values or FDR adjusted q values to lower Type I error.

### Limitations

The data presented and the conclusions reached apply only to a SAMGE-PC design and may not be applicable to cross-sectional studies, as the general advantages of longitudinal, repeated measures designs [62] certainly apply to gene expression studies [16]. The study used endomyocardial biopsies taken from the distal RV septum as starting material for RNA extraction. Although the septum is an interventricular shared wall whose gene expression tracks with LV [6,8,63] and RV [63] remodeling, tissue from the right side of the septum may not be as representative of LV molecular remodeling as LV biopsies. However, in this study only the LV demonstrated statistically significant reverse remodeling, and therefore the observed changes in gene expression are likely to be representative of LV biologic effects. For cost reasons we confined RNA-Seq to a 12 patient Super-Responder subcohort, and ideally all 47 patients would have had these measurements. The *S-R* subcohort was a subset of the larger *A-S* cohort, and therefore was not independent of the parent population. Finally, since RNA-Seq has become the favored method of measuring transcript abundance investigators may be reluctant to add another platform to their study design, and it is not clear if performing two separate RNA preparation and sequencing runs could substitute for mRNA abundance determination in a completely separate platform. Nevertheless, a cross-platform concordance design performed contemporaneously as opposed to for post hoc confirmation should be considered for *in situ* human gene expression studies, where starting material is limited and repeating experiments is untenable.

## Conclusions

For measurement of myocardial gene expression in sequential studies that include a baseline control value, among RT-qPCR, microarray and RNA-Seq methods there was no convincing advantage of one platform over another. However, for measurements of both candidate and global gene expression the use of 2 platforms had an advantage over a single platform, allowing for the use of cross-platform concordance as a more robust measure of biologically-related changes in gene expression. The basis for the 2-platform concordance advantage is likely related to the derived alpha calculation of 0.00125, but also to the requirement that independent measurement technologies yield conventional significance levels. Measurement concordance is more valuable for global gene expression, where the false discovery rate is higher than for candidate genes preselected for potential involvement in the remodeling process, but concordance also adds value in a candidate/hypothesis driven setting. Multiple lines of evidence favored the use of the larger but less reverse remodeled *A-S* cohort over the smaller, more improved phenotype *S-R* subcohort, and global gene expression measurements were only available in *S-R*. Nevertheless, a 42% C_ccT_ between *A-S* and *S-R* for global R/NR changes indicated that for this analysis the *S-R* subcohort was representative of the *A-S* cohort. Finally, both IPA and biologic category analyses support an improvement in calcium signaling as the major gene expression correlate of reverse remodeling, consistent with the improvement in systolic function that is associated with improved LVEF as well as previous work in model systems.

IPA and Superheat map investigation of concordant gene expression changes in the R/NR global gene expression analysis in *S-R* identified the likely involvement of unexpected canonical pathways in the reverse remodeling process, as well as potentially large networks involving β-AR and possibly GNAI2 regulation.

The data support the advantage of sequential measurements of gene expression before and after organ phenotype modification, particularly if phenotype responders can be compared to nonresponders in an R/NR design.

Measuring human ventricular myocardial gene expression in situ by serial endomyocardial biopsy remains a research tool only, but has the potential, particularly by employing multi-platform measurements in larger cohorts undergoing reverse remodeling, to identify novel mechanisms important in myocardial failure and hypertrophy.

## Supporting information

S1 Text List of abbreviations and acronyms. (DOCX)

S2 Text Ingenuity Pathway Analysis (IPA) Background. (DOCX)

S1 Table List of tables comparing platforms in *A-S* and *S-R* cohorts, or cohort comparisons. (DOCX)

S2 Table Up or downregulated genes within the R and R/NR analyses, microarray or RNA-Seq measurements in the *S-R* cohort. (DOCX)

S3 Table Biologic categories of 299 concordant gene expression changes identified in the R/NR analysis by microarray and RNA-Seq in the *S-R* cohort (Table 3), and RT-qPCR and RNA-Seq in the *S-R* cohort (S1 Table). (DOCX)

S4 Table Up or downregulated genes within the R and R/NR analyses, microarray measurements in the *A-S* cohort. (DOCX)

S5 Table Cross-cohort concordant global gene expression changes, mRNA measurements by microarray in the *A-S* and *S-R* cohorts, Responder and R/NR datasets. (P <0.05 in each cohort).
(DOCX)

S6 Table Gene expression changes from baseline within LVEF change tertiles. P <0.05 by Wilcoxon signed rank for change from baseline.
(DOCX)

S7 Table IPA canonical pathways with overlap P values < 0.05, microarray, RNA-Seq and RT-qPCR mRNA measurements in the R/NR analysis. N = 299 concordant gene mRNA changes in the *S-R* cohort biologically classified in S3 Table.
(DOCX)
