# Correction: Sequential analysis of myocardial gene expression with phenotypic change: Use of cross-platform concordance to strengthen biologic relevance

**DOI:** 10.1371/journal.pone.0224389

**Published:** 2019-10-22

**Authors:** Lee S. Toni, Ian A. Carroll, Kenneth L. Jones, Jessica A. Schwisow, Wayne A. Minobe, Erin M. Rodriguez, Natasha L. Altman, Brian D. Lowes, Edward M. Gilbert, Peter M. Buttrick, David P. Kao, Michael R. Bristow

There are a number of errors in the caption for Fig 3, “Ingenuity pathway analysis of concordantly changed mRNAs from Table 3,” panels A-D. Please see the complete, correct Fig 3 caption here.

**Fig 3:**
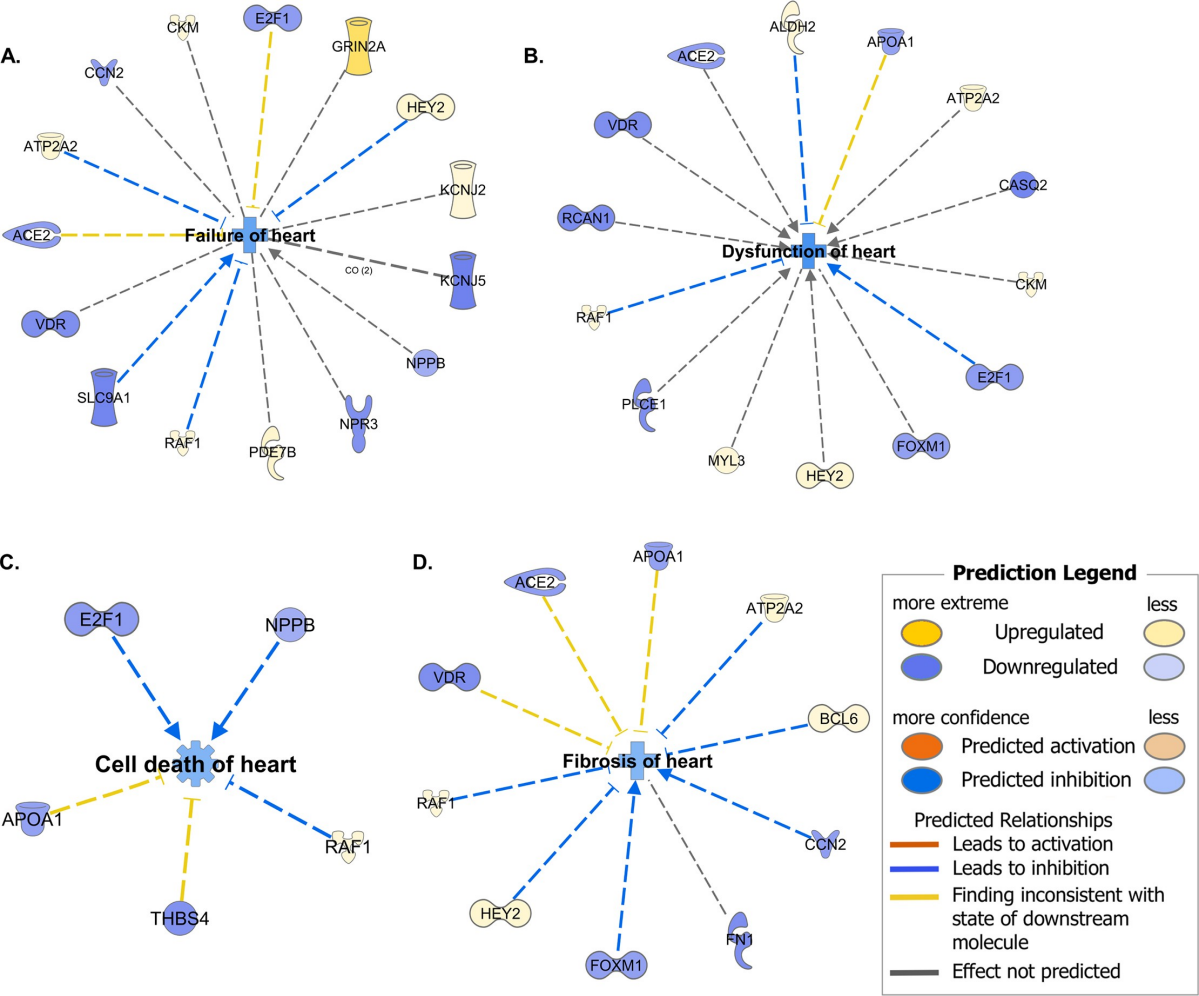
Ingenuity pathway analysis of concordantly changed mRNAs from Table 3. Colors indicate predicted effect of transcript on biological function or disease in center of circular diagram. (**A**) Failure of heart. (**B**) Dysfunction of heart. (**C**) Cell death of heart. (**D**) Fibrosis of heart.

The caption listed within Supporting Information file S1 Text is incorrect. Additionally, there are a number of errors in the gene names in S2 Table, S3 Table and S4 Table. Please view the correct S1 Text, S2 Table, S3 Table and S4 Table below.

Note, there is an error in the caption for S3 Table. Please see the complete, correct S3 Table caption below.

## Supporting information

S1 Text List of abbreviations and acronyms. (DOCX)

S2 Table Up or downregulated genes within the R and R/NR analyses, microarray or RNA-Seq measurements in the *S-R* cohort. (DOCX)

S3 Table Biologic categories of 299 concordant gene expression changes identified in the R/NR analysis by microarray and RNA-Seq in the *S-R* cohort (Table 3), and RT-qPCR and RNA-Seq in the *S-R* cohort (Table 2). (DOCX)

S4 Table Up or downregulated genes within the R and R/NR analyses, microarray measurements in the *A-S* cohort. (DOCX)
