# Supplementary material for: Sequential analysis of myocardial gene expression with phenotypic change: Use of cross-platform concordance to strengthen biologic relevance
Source: PLoS One. 2019 Aug 30;14(8):e0221519. doi: 10.1371/journal.pone.0221519 (PMC6716635; doi:10.1371/journal.pone.0221519)
Supplement: S1 Text — (DOCX) [file pone.0221519.s001.docx]

**S1 Table. List of tables comparing platforms in *A-S* and *S-R* cohorts, or cohort comparisons*.***

***A-S*** - All-subjects cohort, N = 47 (31 Responders and 11 Nonresponders)

**C_cpT_** _-_ Total Cross-Platform Concordance

**C_ccT_** - Total Cross-cohort concordance

**DEG** - Differentially expressed genes

**FPKM** - Fragments per kilobase of transcript per million mapped reads

**HFrEF** - Heart failure with reduced left ventricular ejection fraction

**HFpEF** - Heart failure with preserved left ventricular ejection fraction

**IPA** - Ingenuity Pathway Analysis

**LVEF** - Left ventricular ejection fraction

**LOCF** - last observation carried forward

**NR** – Nonresponder for reverse remodeling, not meeting the definition of Responder in the A-S cohort

**POG** - Percentage of overlapping genes

**R** - Responder for reverse remodeling, predefined as an LVEF increase from baseline at 3 months of ≥5 absolute %, or an increase at 12 months of ≥8 absolute %.

**R/NR** - Responder mRNA abundance change from baseline/Nonresponder change

**RVEF** - Right ventricular ejection fraction

**SAMGE-PC** - sequential analysis of myocardial gene expression with phenotypic change

***S-R*** *-* Super-Responder subcohort, N = 12 (6 Super-Responders and 6 Nonresponders)

**SR** - Super-Responder for reverse remodeling, predefined as an LVEF increase from baseline at 3 or 12 months of ≥10 absolute %
