# Supplementary material for: Sequential analysis of myocardial gene expression with phenotypic change: Use of cross-platform concordance to strengthen biologic relevance
Source: PLoS One. 2019 Aug 30;14(8):e0221519. doi: 10.1371/journal.pone.0221519 (PMC6716635; doi:10.1371/journal.pone.0221519)
Supplement: S2 Text — (DOCX) [file pone.0221519.s002.docx]

**S2 Text. Ingenuity Pathway Analysis (IPA) Background.**

IPA utilizes a suite of statistical algorithms and tools [24] that enable investigators to predict downstream biological effects, such as activation or inhibition of canonical signaling pathways or diseases, based on the input gene expression dataset(s). Biological effects receive an activity status based on z-score. An activity status of NA (Not Applicable) indicates insufficient published data exists for a given set of molecules within a dataset and as such no conclusion about status can be made. Similarly, when the proportion of activating or inhibiting molecules is approximately equal, a z-score of 0 is given indicating that the activity status is neutral, being neither activated nor inhibited. In all other cases, the totality of molecules within a given dataset trend towards either activation or inhibition when z-scores are positive or negative, respectively. A z-score of > 2 or < - 2 is considered statistically significant; values between < 2 and 0 or > - 2 and 0 are considered trends toward activation or inhibition.

The other statistical parameter calculated in the IPA analysis is the “Overlapping P-value”, determined by Fisher’s exact test of the likelihood that the number of genes with changed expression in the experimental dataset is related to the number of genes in the IPA database for a particular category. A P of -Log > 1.30 (<0.05) indicates that the number of genes with changes expression has a < 5% chance of being random with respect to a particular canonical pathway or network, and -Log >2.0 designates a P <0.01.

**References**

24. Krämer A, Green J, Pollard J, Tugendreich S. Causal analysis approaches in ingenuity pathway analysis. *Bioinformatics.* 2014;30:523-530.
