# Supplementary material for: Sequential analysis of myocardial gene expression with phenotypic change: Use of cross-platform concordance to strengthen biologic relevance
Source: PLoS One. 2019 Aug 30;14(8):e0221519. doi: 10.1371/journal.pone.0221519 (PMC6716635; doi:10.1371/journal.pone.0221519)
Supplement: S1 Table — (DOCX) [file pone.0221519.s003.docx]

**S1 Table**. **List of tables comparing platforms in *A-S* and *S-R* cohorts, or cohort comparisons.**

| **Gene**  **Category** | **RT-qPCR**  **n = 50 candidate genes** | | **Microarray**  **n = 19,672 Global Genes^§^** | | **RNA-Seq**  **N = 15,366 Global**  **Genes^§^** | |
| --- | --- | --- | --- | --- | --- | --- |
| **Candidate Genes** | ***A-S***^†^ | ***S-R***^‡^ | ***A-S***^†^ | ***S-R***^‡^ | ***A-S***^†^ | ***S-R***^‡^ |
|  | Table 1 | Table 2 | Table 1 | Table 2 | − | Table 2 |
|  | Table 5* | Table 5* | S4 Table | S2 Table | − | S2 Table |
|  | − | − | − | Table 3 | − | Table 3 |
| **Global Genes** | − | − | S4 Table | S2 Table | − | S2 Table |
|  | − | − | − | Table 3 | − | Table 3 |
|  | − | − | S5 Table* | S5 Table* | − | − |

*Comparison of *A-S* and *S-R* cohorts; ^†^*A-S* cohort, n = 47; ^‡^*S-R* cohort, n = 12); ^§^includes

candidate genes.*
