# Supplementary material for: Sequential analysis of myocardial gene expression with phenotypic change: Use of cross-platform concordance to strengthen biologic relevance
Source: PLoS One. 2019 Aug 30;14(8):e0221519. doi: 10.1371/journal.pone.0221519 (PMC6716635; doi:10.1371/journal.pone.0221519)
Supplement: S3 Table — (DOCX) [file pone.0221519.s005.docx]

**S3 Table. Biologic categories of 299 concordant gene expression changes identified in the R/NR analysis by microarray and RNA-Seq in the *S-R* cohort** **(Table 3), and RT-qPCR and RNA-Seq in the *S-R* cohort (S1 Table).**

| **Category** | **Upregulated** | **Downregulated** | | **Category** | **Upregulated** | | **Downregulated** |
| --- | --- | --- | --- | --- | --- | --- | --- |
| Contractile and associated proteins | LRRC39, MYL3, MYLK4;  **N = 3** | TNNT1, TPM3;  **N = 2** | | Cytoskeleton | **N = 0** | | ACTN1, CKAP4, FARP1, SHROOM3, SPTAN1, SVIL;  **N = 6** |
| Metabolism | ATP5I, CHDH, CKM, GALNTL1, HADHB, HMGCS2, NDUFB9, TKTL1;  **N = 8** | ACAD11, ALDH3A2, APOA1, APOL4, CYP19A1, CYP11A1, MPV17, OGDHL, SDSL;  **N = 9** | | Gene regulation: TFs, RNA binding, nt processing, nucleosome, translation, noncoding RNAs | BCL6, HEY2, HIST3H2A, KAT2B, LARP4B, MRPL43, MRPS24, POLR2I, RORC, RPL24, UCKL1, ZBED5, ZNF33B, ZNF585A;  **N = 14** | | BEX1, E2F1, EIF4G3, ENC1; CDK8, CENPA, CENPN, CHD3, CUX1, FOXM1, HELB, HLX, HMGB3, JAZF1, KIF4A, MAGED2, MPV17L2, NCAPH, PGBD5, POLR1E, SETD3, TFDP2, TOP2A, MEG3 (LncRNA);  **N = 24** |
| Growth/hypertrophy Regulation | ADCYAP1R1, ALDH2, AMD1, CORIN, DLK1, MTSS1, PEBP4, PLAG1;  **N = 8** | ENO3, CDC25A, CDH17, CDH2, CRELD1, EXOC6B, GRN, HTRA1, LYPD1, MEST, MYOZ3, NEB, NPPB, NPR3, ODC1, PAPPA2, PTPRH, QPCT; RCAN1, SPINT2, WDR77;  **N = 21** | | cAMP regulation | ART3, PDE7B,  **N = 2** | | PDE1A, PDE8B, DDA1  **N = 3** |
| Channels, Solute Exchangers, Transporters | AQP7, GRIN2A, KCNJ2, SCN1A, SLC19A2, SLC26A9, SLC27A6, SCN7A;  **N = 8** | ABCD1, KCNC4, KCNJ4, KCNJ5, SLC1A4, SLC1A7, SLC9A1;  **N = 7** | | NH Signaling | PPP1R1A;  **N = 1** | | ACE2;  **N = 1** |
| Ca^2+^ Handling or signaling | ATP2A2;  **N = 1** | CASQ1, CASQ2;  **N = 2** | | PI3K, PLC, LPA signaling | PLA2G4F, PLCL2, LPAR3;  **N = 3** | | INPP4B, MTMR3, PIK3R2, PLCD4, PLCE1, SNCA, SHC2;  **N = 7** |
| Cell Homeostasis  (Golgi, ER, mem-brane and cytosol trafficking | ADAM11, CACYBP, COLEC12, HSPB3, SEPP1 (SELENOP), SYT3, THAP1, TJP2  **N = 8** | CALU, DHCR24, CHMP4B, CHMP4C, CLTCL1, ENOX2, EXT1, EXTL3, HCCS, HEXB, PACSIN1, PCSK5, PLIN3, PORCN, SGTA, SH3GL2, ST3GAL4, SYTL4, SYTL5, TGOLN2, TMED3, TMEM51, TSPAN5, UBR1, XPO4;  **N = 25** | | Small GTPases, regulators (RAS, RGS, GEF) | MCF2, MLPH, RAF1;  **N = 3** | | RASL11B, RGS4, RRAS, TBC1D22A. IQGAP3, RAB15, RAB31, RAB6B;  **N = 8** |
| Fibroblast growth, Extracellular Matrix  or TGFβ-signaling | COL28A1, FGFBP2, FNDC5;  **N = 3** | ARSD, ARSE, BGN, CHPF2, COL18A1, COL1A1, COL23A1, COL4A1, CTGF, FN1; GDF11, LOXL1, LOXL2, LTBP2, MXRA5, PI16, QSOX1, SERPINE2, SMAD6, SMOC2, THBS4;  **N = 21** | | Cytokines and downstream signaling | ASB10, IL6R;  **N = 2** | | CRLF1, HERC5, ORMDL3, SOCS2;  **N = 4** |
| Apoptosis | BCL11A, VRK2;  **N = 2** | AATF, ARHGAP11A, LGALS9, RELL1, SH3GLB1, SHB, STK17A, TPD52L1;  **N = 8** | | Other signaling | PHACTR3, PIM3, RET, SHISA3, WNT5A;  **N = 5** | | ABAT, ABHD12, APLP1, ATP6V1E2, SGK1, PDLIM4, PENK, SIRPA, TRIM41;  **N = 9** |
| Microtubules | **N = 0** | MAP1A, MAP2, MAP7;  **N = 3** | | AKAP related | **N = 0** | | RSPH3;  **N = 1** |
| Immune function  other than cytokines | CD5L, CPA3, GZMK, SDK1, TPSAB1;  **N = 5** | SH2D4A, TSTA3;  **N = 2** | | Unclassified/  Unknown Function  in the heart | C1orf105, C3orf43, C7orf70, CEL, DH11, FAM124A, FAM179A, KIAA1328, MRO, NCR00201, PRELID2, SYCP3, TMEM132C, TPRKB, WDSUB1, RNF165,  **N = 16** | | ADC, ASPM, ATRNL1, C11orf24, C1orf21, C5orf46, C7orf53, C9orf30, CC2, DCBLD2, DLK2, DOK4, CCNJL, KCB1, KIAA0556, KIAA1244, KIAA1539, KLHL6, LGI2, LNP1, LUZP1, MELK, MFI2, MLLT11, MOXD1, PHYHIP, DSYN1, P1L4, NOTCH2NL, PDPN, RNF214, SRPX2, TPX2, UNC13C, WHSC1, ZMYND17, ZNRF2, 42989, 42797, 42982,  **N = 40** |
| Vascular/thrombosis | VWA3A;  **N = 1** | PROS1, SVEP1, VDR;  **N = 3** | |  |  | |  |
| - | | | | | | | |
| **Aggregated Extracellular to Intracellular Signaling** | | | **Upregulated** | | | **Downregulated** | |
| cAMP regulation | | | 2 | | | 3 | |
| NH Signaling | | | 2 | | | 1 | |
| PI3K, PLC, LPA signaling | | | 3 | | | 7 | |
| Small GTPases, regulators (RAS, RGS, GEF) | | | 3 | | | 8 | |
| Cytokines and their downstream signaling | | | 2 | | | 4 | |
| AKAP related | | | 0 | | | 1 | |
| Other signaling | | | 5 | | | 9 | |
| Total | | | 17 | | | 33 | |
