# Supplementary material for: Sequential analysis of myocardial gene expression with phenotypic change: Use of cross-platform concordance to strengthen biologic relevance
Source: PLoS One. 2019 Aug 30;14(8):e0221519. doi: 10.1371/journal.pone.0221519 (PMC6716635; doi:10.1371/journal.pone.0221519)
Supplement: S5 Table — (P <0.05 in each cohort). (DOCX) [file pone.0221519.s007.docx]

**S5 Table. Cross-cohort concordant global gene expression changes**, **mRNA measurements by microarray in the *A-S* and *S-R* cohorts, Responder and R/NR datasets.** (P <0.05 in each cohort).

| **R** | | **R/NR** | | | | | | | | | | | | | |
| --- | --- | --- | --- | --- | --- | --- | --- | --- | --- | --- | --- | --- | --- | --- | --- |
| **Up-regulated** | **Down-regulated** | **Upregulated** | | | | | | | **Downregulated** | | | | | | |
| ALS2CR11 | AKAP5 | A2BP1 | CHORDC1 | FTO | LOC401-  397 | PIGY | SHISA3 | YEATS4 | AAA1 | CCNT1 | ESCO2 | KRT15 | MOBKL2C | RC3H2 | TMEM51 |
| ARHGAP21 | ARPC4 | ABHD3 | CHPT1 | FUNDC2 | LOC4-  40552 | PION | SHPRH | YWHAG | AATF | CCT6A | ESPL1 | LAD1 | MRTO4 | RCAN1 | TMEM90B |
| BCL2A1 | C19orf33 | ACADM | CKM | GALNTL1 | LOC64-  5676 | PKIA | SIRT1 | ZBED5 | ACAD11 | CDC14B | EXTL3 | LAMB2 | MSTO1 | RDBP | TNMD |
| BMP2 | C21orf93 | ACN9 | CLDN12 | GAS2 | LOC64-  6903 | PLA2G4F | SIRT4 | ZBTB11 | ACE2 | CDC25A | FAH | LENG1 | MTMR3 | RGS11 | TNNI3 |
| C10orf10 | CCDC1-  44A | ACSL1 | CLDND1 | GHR | LSM5 | PLAG1 | SLC16A7 | ZBTB26 | ACER1 | CDH2 | FAM22F | LEPREL1 | MUSTN1 | RGS4 | TNNT1 |
| C1orf110 | CENPH | ADAM11 | CLGN | GIMAP8 | LYPLA1 | PLCL1 | SLC27A6 | ZBTB44 | ACPL2 | CDK3 | FARP1 | LGI2 | MXRA5 | RP11-529I10.4 | TOP2A |
| C5orf28 | COX6A1 | ALDH2 | CLPX | GLRX5 | LYPLAL1 | PLN | SLC35F1 | ZC3H6 | ACTN1 | CDK8 | FBXO15 | LHB | P1L4 | RRAS | TPM3 |
| C8orf12 | CTXN3 | AMN1 | CMC1 | GNG5 | LYRM1 | PMP22 | SLC38A2 | ZFP112 | ACTR8 | CDKN2BAS | FCRLB | LIMK1 | NDE1 | RSPH3 | TPX2 |
| CCDC111 | DAB2IP | ANKRD13C | COG5 | GPBP1 | LYRM5 | PMPCB | SLC7A-  6OS | ZMPST-  E24 | AFFX-LysX-5_at | CENPA | FDXR | LOC10012-  8098 | NEK2 | RUNDC3A | TSXIP1 |
| CCDC142 | DIAPH1 | ANKRD29 | COL28A1 | GPN3 | MAF | PNMA6A | SMURF2 | ZNF138 | AKAP5 | CENPI | FERMT2 | LOC10013-  0502 | NF2 | RWDD2A | TSPAN5 |
| CD300A | EGLN3 | ANO5 | COLEC12 | GPR146 | MAMDC2 | POLR2I | SNRNP27 | ZNF140 | ALDH3A2 | CEP55 | FLJ39051 | LOC10013-  0964 | NFX1 | S100A2 | TSTA3 |
| CPSF4 | FLJ39080 | APPL1 | COMMD3 | GPR22 | MBLAC2 | PPP1R15B | SNX3 | ZNF17 | ALPK3 | CETP | FLJ39080 | LOC10013-  1031 | NOTCH2NL | SAPS3 | UBE2T |
| CSAD | FUCA2 | AQP7 | COPB1 | GPRC5B | MCF2 | PPP1R1A | SP140 | ZNF2 | AMAC1 | CHAF1A | FLJ90757 | LOC10021-  6479 | NPC1L1 | SCN3B | UBR1 |
| CXorf65 | GAS7 | ARL5A | COQ3 | GSDMC | MCM3A-  PAS | PPP6C | SP3 | ZNF236 | ANLN | CHD3 | FN1 | LOC144481 | NPPB | SERGEF | UCK2 |
| CYP39A1 | IPO9 | ART3 | CORIN | HADHB | MCM9 | PRELID2 | SPHKAP | ZNF252 | APEX2 | CHEK1 | FREQ | LOC152742 | NPR3 | SERPINE2 | UCN |
| DDHD2 | IRAK4 | ASB10 | COX11 | HCG4 | MED4 | PRKRA | SRP9 | ZNF253 | APLP1 | CHMP4B | FUCA2 | LOC159110 | NSUN4 | SETD3 | UGGT1 |
| DDX10 | KIAA0746 | ATAD1 | COX16 | HDGF | MLPH | PRNP | STAG1 | ZNF260 | APOA1 | CKAP2L | FZR1 | LOC256021 | NTS | SGK1 | UNC13C |
| DHFRL1 | KLF16 | ATP5C1 | COX5A | HEATR5A | MOBKL1A | PRRG1 | STEAP2 | ZNF302 | APOL4 | CKAP4 | GABRD | LOC283999 | NUDT5 | SGK269 | VTI1B |
| DMGDH | KP1 | ATP5G3 | COX7B | HELQ | MRO | PS1TP4 | STT3B | ZNF319 | ARHGAP-  11A | CKAP5 | GAFA1 | LOC284112 | OCLN | SGSH | VTN |
| EGF | LMTK2 | ATP5I | COX7C | HEY2 | MRPL43 | PSMA8 | STXBP1 | ZNF33B | ARMC9 | CLTCL1 | GARNL3 | LOC284576 | ODC1 | SGTA | WBSCR17 |
| EZH1 | LOXHD1 | ATP5J | CPA3 | HIBCH | MRPL48 | PTDSS1 | SUCLA2 | ZNF382 | ARPC5L | CNTD2 | GARS | LOC284889 | OGDHL | SH2D4A | WDR66 |
| FAM168A | LUZP1 | ATPBD4 | CPNE4 | HIF1A | MRPL51 | PTGER4 | SYT3 | ZNF383 | ASPM | COL18A1 | GINS2 | LOC285423 | ORAI2 | SH3GL2 | WDR69 |
| FEZ1 | MCM2 | BBS10 | CREBBP | HINT1 | MRPS24 | PTGES3 | TAF5 | ZNF441 | ATP6V1E2 | COL1A1 | GNG13 | LOC339803 | ORMDL3 | SH3GLB1 | WFDC3 |
| GALNTL2 | MEX3D | BCAR3 | CS | HISPPD1 | MTERFD1 | PTGR2 | TARDBP | ZNF493 | ATRNL1 | COL22A1 | GNL3L | LOC339894 | OSTalpha | SHB | WHSC1 |
| GAS2 | MSR1 | BCL2L11 | CTAGE5 | HIST3H2A | MTF2 | RAB12 | TCEAL1 | ZNF578 | BFSP1 | COLEC10 | GPR32 | LOC339929 | PCBP3 | SIRPA | XPO4 |
| GSTM4 | NR4A1 | BCL6 | CTR9 | HMGB1 | MTSS1 | RAB33B | THAP1 | ZNF585A | BGN | COQ10B | GPR37 | LOC340107 | PDCL2 | SLC35F4 | YIF1A |
| GTF2IRD2 | PAFAH-  1B2 | BMI1 | CXorf24 | HPGDS | MYL3 | RAP2C | THNSL1 | ZNF623 | BIRC5 | CPNE2 | GPR85 | LOC400456 | PDE8B | SLC43A3 | ZMYND12 |
| HMGCLL1 | PRKCA | BMPER | DEK | HSPB3 | MYLK4 | RB1CC1 | THUMPD1 | ZNF706 | BMP6 | CRIP3 | GPX2 | LOC401312 | PDE9A | SLC4A8 | ZMYND17 |
| HSD17B4 | RQCD1 | BRMS1L | DJB9 | IER3IP1 | MYST4 | RBM12B | TJP2 | ZNF781 | BUB1 | CSMD3 | GTSE1 | LOC439990 | PDK3 | SLC9A1 | ZNF259P |
| IGSF10 | S1PR2 | BTBD1 | DPY19L2 | IL6R | P1L2 | RBMX | TKTL1 | ZNF828 | C10orf114 | CSPG4 | HAPLN3 | LOC51145 | PDLIM7 | SMAD6 | ZNF483 |
| KDM6A | SAMD14 | BTBD12 | DPYSL2 | ITGB6 | NCOA4 | RET | TMEM182 | ZNF92 | C10orf68 | CTA-216E10.6 | hCG_204-  5206 | LOC541471 | PENK | SMOC2 | ZNF625 |
| KLHL26 | SELI | C11orf46 | DSP | ITK | NDUFA4 | RFXAP | TPRKB |  | C11orf52 | CTGF | HERC5 | LOC643733 | PFKL | SPC2 | ZNF142 |
| LOC10012-  8288 | SIPA1L3 | C11orf87 | EBAG9 | KAT2B | NDUFAB1 | RG9MTD2 | TRDN |  | C11orf63 | CUX1 | HEXB | LOC643923 | PHF21B | SNCA | ZNF404 |
| LOC145-  820 | SMAD2 | C12orf39 | EFHA1 | KCNJ2 | NDUFB1 | RHBDD1 | TRMT61B |  | C14orf128 | CWH43 | HIST1H-  2BA | LOC646588 | PHTF1 | SNCAIP |  |
| LOC73-  1884 | STAG3L4 | C13orf30 | EFHA2 | KGFLP2 | NDUFB3 | RHOT1 | TSHZ1 |  | C14orf182 | CYLC1 | HMGA2 | LOC727916 | PLCD4 | SNRPN |  |
| MTERFD2 | TFDP2 | C15orf61 | EID2B | KIAA0528 | NDUFB4 | RICS | TSPYL5 |  | C14orf37 | CYP19A1 | HMGB3 | LOC731223 | PLCE1 | SOCS2 |  |
| MTHFD2 | TGFB2 | C16orf52 | EIF1AX | KIAA0895 | NDUFB5 | RIOK2 | TTC31 |  | C14orf50 | CYP2C18 | HOMER3 | LOC81691 | PLEKHA8 | SOHLH2 |  |
| ALAD2 | TMEM165 | C16orf54 | ENOSF1 | KIAA1012 | NDUFB6 | RNF165 | TTC7B |  | C16orf75 | CYP51A1 | HPD | LOXL1 | PLEKHG2 | SPC25 |  |
| NHEJ1 | TRPM1 | C18orf54 | ENPP4 | KIAA1143 | NDUFB8 | RNLS | TUBA3D |  | C16orf89 | DAK | HSPA4 | LRP8 | PLEKHH2 | SPINT2 |  |
| NUDT4 | TSXIP1 | C1orf186 | EPS15 | KIAA1267 | NDUFB9 | RPL15 | TUSC1 |  | C19orf51 | DAP | HTRA1 | LRRC1 | PLX1 | SPTAN1 |  |
| PHACTR3 | TSPAN17 | C20orf177 | ESRRG | KIAA1466 | NDUFS4 | RPL21 | TXNL1 |  | C1orf135 | DCBLD2 | IFI27L2 | LRRC27 | PMEPA1 | SRPK2 |  |
| PKD1L1 | VTI1B | C20orf24 | EXPH5 | KL | NHLRC2 | RPL27 | UBA5 |  | C1orf21 | DDA1 | IF8 | LRRC56 | PMS2L5 | SRPX2 |  |
| PPP6C |  | C21orf87 | FAM104B | KLHL15 | NKAP | RPL31 | UBE2B |  | C1orf230 | DDAH2 | INPP5F | LRRC70 | POLQ | SSH2 |  |
| PRELID2 |  | C2orf40 | FAM124A | KLRK1 | NKIRAS1 | RPL37A | UBE2D1 |  | C1orf91 | DDR1 | IPO9 | LTBP2 | POLR1E | ST7L |  |
| RBM4B |  | C3orf43 | FAM127B | KRIT1 | NNT | RPL9 | UBE2E3 |  | C20orf54 | DDX4 | IQCD | LUZP1 | PPFIA3 | ST8SIA5 |  |
| SEC16B |  | C4orf32 | FAM162A | LACTB2 | NR2C2 | RPS13 | UBE2K |  | C20orf94 | DECR2 | IQGAP3 | LYAR | PPP1R7 | STAG3 |  |
| SLC26A9 |  | C7orf70 | FAM175A | LCORL | NR3C2 | RPS17P5 | UBR3 |  | C5orf46 | DEPDC1 | IRAK4 | MAGEA10 | PRC1 | STC1 |  |
| SLC38A4 |  | C8orf37 | FAM179A | LDB3 | NRXN1 | RRM2B | UCKL1 |  | C7orf53 | DFFA | JAZF1 | MAGED2 | PRELID1 | STGC3 |  |
| SLC41A1 |  | C8orf45 | FAM63B | LIN7C | NUDT19 | RSBN1L | UHRF2 |  | C8orf58 | DGCR9 | KCNC4 | MAP1LC3C | PREP | STK17A |  |
| SMAR-  CAD1 |  | CA14 | FAM8A1 | LOC10013-  1512 | NUP43 | RWDD4A | UQCRB |  | C9orf100 | DIAPH3 | KCNJ4 | MAP2 | PRMT10 | SUSD5 |  |
| SPATA9 |  | CACNB2 | FBXL4 | LOC10013-  2418 | OTUD6B | RYR2 | UQCRFS1 |  | C9orf30 | DIRAS2 | KCNQ4 | MAS1 | PRSS35 | SYTL4 |  |
| SYCP3 |  | CACYBP | FDX1 | LOC153-  346 | P2RY14 | SCGB1D2 | VBP1 |  | CALU | DLGAP1 | KCTD10 | MCM2 | PTPN4 | TACC3 |  |
| TCEAL1 |  | CAPZA2 | FGFBP2 | LOC157-  381 | PATZ1 | SCML2 | VDAC2 |  | CAMK2N2 | DNER | KCTD11 | MELK | PTTG1 | TAS1R2 |  |
| TLR3 |  | CCDC111 | FKBP9 | LOC255-  512 | PCF11 | SCN1A | VWA3A |  | CARHSP1 | DOK4 | KDELR3 | MFI2 | PXDN | TGOLN2 |  |
| TRIM45 |  | CCPG1 | FKTN | LOC283-  588 | PCTK2 | SCN7A | WDR51B |  | CASC5 | DTL | KIAA0226 | MGAT2 | QPCT | THBS4 |  |
| TUBD1 |  | CD5L | FLJ31713 | LOC28-  6052 | PEBP4 | SDCBP | WDSUB1 |  | CASQ1 | E2F1 | KIAA0556 | MGC2752 | RAB15 | THY1 |  |
| ZDHHC6 |  | CDKN1B | FLJ36644 | LOC28-  6178 | PEG3 | SDR39U1 | WNT5A |  | CBLN4 | ELF5 | KIAA1539 | MGC4294 | RAB31 | TK1 |  |
| ZNF135 |  | CECR6 | FMR1 | LOC34-  0544 | PFAS | SEPP1 | WWOX |  | CCDC11 | ENC1 | KIF14 | MIF | RAD54L | TMED3 |  |
| ZNF142 |  | CEL | FNDC5 | LOC38-  8387 | PFKFB3 | SGMS1 | WWP1 |  | CCDC125 | ENOX2 | KIF4A | MKI67 | RANBP1 | TMEM127 |  |
| ZNF404 |  | CHDH | FPGT | LOC38-  9834 | PIGM | SH3KBP1 | XYLT1 |  | CCDC96 | EPR1 | KIF9 | MND1 | RASL11B | TMEM185A |  |
| **Ns (concordant changes/total changes)** | | | | | | | | | | | | | | | |
| 60/2931  (*A-S*);  60/2294  (*S-R*) | 42/3274  (*A-S*;  42/2095  (*S-R*) | 391/850  (*A-S*)  391/869  (*S-R*) | | | | | | | 393/1085  (*A-S*)  393/936  (*S-R*) | | | | | | |
| **Total Cross-Cohort Concordance (C_ccT_), up and downregulated genes** | | | | | | | | | | | | | | | |
| **204/10,594**  **(1.9%)** | | **1,568/3,740**  **(42%)** | | | | | | | | | | | | | |
