# Supplementary material for: Sequential analysis of myocardial gene expression with phenotypic change: Use of cross-platform concordance to strengthen biologic relevance
Source: PLoS One. 2019 Aug 30;14(8):e0221519. doi: 10.1371/journal.pone.0221519 (PMC6716635; doi:10.1371/journal.pone.0221519)
Supplement: S6 Table — P <0.05 by Wilcoxon signed rank for change from baseline. (DOCX) [file pone.0221519.s008.docx]

**S6 Table. Gene expression changes from baseline within LVEF change tertiles.** P <0.05 by Wilcoxon signed rank for change from baseline.

|  | **Tile 1** | | | **Tile 2** | | | **Tile 3** | | |
| --- | --- | --- | --- | --- | --- | --- | --- | --- | --- |
|  | **RT-qPCR** | **Array** | **Concordant** | **RT-qPCR** | **Array** | **Concordant** | **RT-qPCR** | **Array** | **Concordant** |
| **Upregulated** | NPPB |  |  | NPPB | ADRB1 | ADRB1 | ADRB1* | ADRB1 | ADRB1 |
|  | THRA1 |  |  | THRA1 | PLN |  | PLN* | PLN* | PLN |
|  |  |  |  | NPPA | ADRB2 |  | THRA1 | ADRB2* | THRA1 |
|  |  |  |  | ADRB1 | AGTR1 |  | (MyL3)* | RyR2* |  |
|  |  |  |  |  | RyR2 |  | (MYH6)* | THRA |  |
|  |  |  |  |  | (MYL3)* |  |  | AGTR1 |  |
|  |  |  |  |  | (MYH6)* |  |  | MyL3* |  |
|  |  |  |  |  |  |  |  | HNRNPD |  |
|  |  |  |  |  |  |  |  | (MYH6)* |  |
| N | 2 | 0 | 0 | 4 | 5 | 1 | 3 | 8 | 3 |
| **Downregulated** | **RT-qPCR** | **Array** | **Concordant** | **RT-qPCR** | **Array** | **Concordant** | **RT-qPCR** | **Array** | **Concordant** |
|  | ADRB2 | MYH6 | MYH6 | ACTC1 | DMD | SLC8A1 | ACTC1 | DMD | HK2 |
|  | MYH6 | HK2 | HK2 | SLC8A1 | SLC8A1 | GNAI2 | SLC8A1 | HK2 | CSRP3 |
|  | ATPA2 |  |  | GNAI2 | GNAI2 | IL6 | NOS2 | CSRP3 | SLC9A1 |
|  | RYR2 |  |  | CTF1 | IL6 |  | GNAI2 | SLC9A1 | ACTA1 |
|  | DMD |  |  | HK2 | ACTA1 |  | CTF1 | ACTA1 | TNNI3 |
|  | ACTC1 |  |  | PDHX | TNNI3 |  | HK2 | TNNI3 | NPPB |
|  | SLC8A1 |  |  | SLC9A1 | NPPB |  | PDHX | NPPB | CASQ2 |
|  | ADRBK1 |  |  | IL6 | EDN1 |  | CSRP3 | CASQ2 | NPPA |
|  | NOS2 |  |  |  |  |  | SLC9A1 | NPPA |  |
|  | GLUL |  |  |  |  |  | ACTA1 |  |  |
|  | GNAI2 |  |  |  |  |  | TNNI3 |  |  |
|  | GNAQ |  |  |  |  |  | TNNC1 |  |  |
|  | PRKCB |  |  |  |  |  | MYL2 |  |  |
|  | CTF1 |  |  |  |  |  | NPPB |  |  |
|  | TNNT2 |  |  |  |  |  | CANX |  |  |
|  | MYL3 |  |  |  |  |  | CASQ2 |  |  |
|  | PDK4 |  |  |  |  |  | NPPA |  |  |
|  | CPT1B |  |  |  |  |  |  |  |  |
|  | HK2 |  |  |  |  |  |  |  |  |
|  | PKFM |  |  |  |  |  |  |  |  |
|  | PDHX |  |  |  |  |  |  |  |  |
|  | CSRP3 |  |  |  |  |  |  |  |  |
|  | SLC9A1 |  |  |  |  |  |  |  |  |
| **N** | 23 | 2 | 2 | 8 | 8 | 3 | 17 | 9 | 8 |
| **C_cpT_^§^** | 4/27 (15) | | − | 8/25 (32) | | − | 22/37 (59) | | − |
|  | P <0.001 | | | | | | | |  |

*P <0.05 by Kruskal-Wallis test; (significant by Kruskal-Wallis across tertiles but not by Wilcoxon rank-sum; ^§^Cross platform total concordance, concordantly changed genes/total changed genes (%).
