# Supplementary material for: Sequential analysis of myocardial gene expression with phenotypic change: Use of cross-platform concordance to strengthen biologic relevance
Source: PLoS One. 2019 Aug 30;14(8):e0221519. doi: 10.1371/journal.pone.0221519 (PMC6716635; doi:10.1371/journal.pone.0221519)
Supplement: S7 Table — N = 299 concordant gene mRNA changes in the S-R cohort biologically classified in S3 Table. (DOCX) [file pone.0221519.s009.docx]

**S7 Table. IPA canonical pathways with overlap P values < 0.05, microarray, RNA-Seq and RT-qPCR mRNA measurements in the R/NR analysis.** N = 299 concordant gene mRNA changes in the *S-R* cohort biologically classified in S3 Table.

| **Ingenuity Canonical Pathway** | **z-score*** | **- log_10_ (P value)^†^** | **Ratio^‡^** | **Molecules** |
| --- | --- | --- | --- | --- |
| Superpathway of Inositol Phosphate Compounds | -1.89 | 1.42 | 0.030 | PTPRH, PLCE1, PPP1R1A, PIK3R2,  PLCD4, SIRPA, CDC25A |
| GP6 Signaling Pathway | -1.63 | 2.05 | 0.045 | COL1A1, COL4A1, COL23A1, PIK3R2,  COL18A1, COL28A1 |
| 3-phosphoinositide Degradation | -1.63 | 1.72 | 0.038 | PTPRH, INPP4B, PPP1R1A, SIRPA,  MTMR3, CDC25A |
| D-myo-inositol-5-phosphate Metabolism | -1.63 | 1.68 | 0.037 | PTPRH, PLCE1, PPP1R1A, PLCD4, SIRPA, CDC25A |
| Dendritic Cell Maturation | -1.63 | 1.34 | 0.031 | COL1A1, PLCE1, PLCL2, PIK3R2,  COL18A1, PLCD4 |
| Adrenomedullin signaling pathway | -1.41 | 2.28 | 0.040 | RAF1, PLCE1, NPR3, RRAS, SHC2, PLCL2, PIK3R2, PLCD4 |
| UVA-Induced MAPK Signaling | -1.34 | 1.79 | 0.045 | PLCE1, RRAS, PLCL2, PIK3R2, PLCD4 |
| Aldosterone Signaling in Epithelial Cells | -1.134 | 3.38 | 0.054 | HSPB3, RAF1, PLCE1, SGK1, PLCL2,  PIK3R2, SLC9A1, PLCD4, HSPA2 |
| Endothelin-1 Signaling | -1.00 | 2.9 | 0.046 | RAF1, PLCE1, RRAS, CASQ1, SHC2,  PLA2G4F, PLCL2, PIK3R2, PLCD4 |
| Melanoma Signaling | -1.00 | 2.06 | 0.066 | RAF1, RRAS, E2F1, PIK3R2 |
| Non-Small Cell Lung Cancer Signaling | -1.00 | 1.61 | 0.045 | RAF1, RRAS, E2F1, PIK3R2 |
| Sperm Motility | -0.816 | 2.16 | 0.047 | NPPB, PLCE1, PLA2G4F, PLCL2, PDE1A, PLCD4 |
| PI3K Signaling in B Lymphocytes | -0.816 | 2.02 | 0.044 | RAF1, PLCE1, RRAS, PLCL2, PIK3R2,  PLCD4 |
| p70S6K Signaling | -0.816 | 1.99 | 0.044 | RAF1, PLCE1, RRAS, PLCL2, PIK3R2,  PLCD4 |
| P2Y Purigenic Receptor Signaling Pathway | -0.816 | 1.96 | 0.043 | RAF1, PLCE1, RRAS, PLCL2, PIK3R2  ,PLCD4 |
| Thrombin Signaling | -0.816 | 1.67 | 0.033 | RAF1, PLCE1, RRAS, PLCL2, PIK3R2,  PLCD4, MYL3 |
| Actin Cytoskeleton Signaling | -0.707 | 1.90 | 0.0343 | RAF1, FN1, RRAS, PIK3R2, SLC9A1,  ACTN1, MYL3, IQGAP3 |
| GDNF Family Ligand-Receptor Interactions | -0.447 | 2.33 | 0.061 | RAF1, DOK4, RRAS, PIK3R2, RET |
| Neuropathic Pain Signaling In Dorsal Horn Neurons | -0.447 | 1.73 | 0.0435 | GRIN2A, PLCE1, PLCL2, PIK3R2, PLCD4 |
| Sphingosine-1-phosphate Signaling | -0.447 | 1.59 | 0.040 | PLCE1, CASQ1, PLCL2, PIK3R2, PLCD4 |
| Apelin Cardiomyocyte Signaling Pathway | -0.378 | 3.11 | 0.062 | ATP2A2, MYL3, PLCL2, PIK3R2, SLC9A1, PLCD4, PLCE1 |
| Glioblastoma Multiforme Signaling | -0.378 | 2.74 | 0.048 | RAF1, PLCE1, RRAS, E2F1, PLCL2,  PIK3R2, PLCD4, WNT5A |
| IL-6 Signaling | -0.378 | 2.68 | 0.052 | HSPB3, COL1A1, RAF1, RRAS, CYP19A1, IL6R, PIK3R2 |
| 14-3-3-mediated Signaling | -0.378 | 2.62 | 0.051 | RAF1, PLCE1, RRAS, PLCL2, PIK3R2,  PLCD4, SNCA |
| CREB Signaling in Neurons | -0.378 | 2.06 | 0.037 | POLR2I, RAF1, GRIN2A, PLCE1, RRAS, PLCL2, PIK3R2, PLCD4 |
| Synaptic Long Term Depression | -0.378 | 1.99 | 0.039 | RAF1, PLCE1, NPR3, RRAS, PLA2G4F, PLCL2, PLCD4 |
| Melatonin Signaling | 0 | 2.587 | 0.069 | RAF1, PLCE1, PLCL2, RORC, PLCD4 |
| Dopamine-DARPP32 Feedback in cAMP Signaling | 0 | 2.80 | 0.049 | GRIN2A, ATP2A2, KCNJ2, PLCL2, KCNJ5, PLCD4, KCNJ4, PLCE1 |
| Opioid Signaling Pathway | 0 | 2.25 | 0.036 | RAF1, GRIN2A, RRAS, KCNJ5, CLTCL1, PENK, SCN7A, RGS4, PDE1A |
| Huntington's Disease Signaling | 0 | 2.2 | 0.036 | POLR2I, SGK1, PACSIN1, CLTCL1,  CASQ1, PENK, PIK3R2, SNCA, HSPA2 |
| Dopamine-DARPP32 Feedback in cAMP Signaling | 0 | 2.2 | 0.043 | KCNJ4, GRIN2A, PLCE1, KCNJ2, KCNJ5, PLCL2, PLCD4 |
| Phospholipases | 0 | 2.04 | 0.064 | PLCE1, PLA2G4F, PLCL2, PLCD4 |
| Wnt/Ca+ pathway | 0 | 2.01 | 0.064 | PLCE1, PLCL2, PLCD4, WNT5A |
| Cardiac Hypertrophy Signaling | 0 | 1.82 | 0.033 | RAF1, PLCE1, RRAS,IL6R, PLCL2, PIK3R2, PLCD4, MYL3 |
| IGF-1 Signaling | 0 | 1.77 | 0.045 | RAF1, CTGF, RRAS, SOCS2, PIK3R2 |
| SPINK1 General Signaling Pathway | 0 | 1.68 | 0.051 | IL6R, RAF1, PIK3R2, RRAS |
| Prolactin Signaling | 0 | 1.51 | 0.045 | RAF1, RRAS, SOCS2, PIK3R2 |
| JAK/Stat Signaling | 0 | 1.51 | 0.045 | RAF1, RRAS, SOCS2, PIK3R2 |
| VEGF Family Ligand-Receptor Interactions | 0 | 1.44 | 0.043 | RAF1, RRAS, PLA2G4F, PIK3R2 |
| STAT3 Pathway | 0 | 1.32 | 0.039 | RAF1, RRAS, SOCS2, CDC25A |
| Synaptic Long Term Potentiation | 0.378 | 2.84 | 0.056 | RAF1, GRIN2A, PLCE1, RRAS, PPP1R1A, PLCL2, PLCD4 |
| Cardiac β-adrenergic Signaling | 0.447 | 1.40 | 0.035 | PPP1R1A, PDE7B, ATP2A2, PDE1A, PDE8B |
| Acute Phase Response Signaling | 0.816 | 2.04 | 0.040 | RAF1, APOA1, FN1, RRAS, IL6R, SOCS2, PIK3R2 |
| Protein Kinase A Signaling | 0.905 | 2.12 | 0.030 | RAF1, PTPRH, PLCE1, PDE7B, PDE8B, PLCL2, PDE1A, PLCD4, SIRPA, MYL3, MTMR3, CDC25A |
| Gα12/13 Signaling | 1.134 | 2.56 | 0.050 | RAF1, CDH2, RRAS, CDH17, PIK3R2,  LPAR3, MYL3 |
| Calcium Signaling | 2.00 | 2.20 | 0.039 | GRIN2A, ATP2A2, MYL3, CASQ2, RCAN1, TPM3, TNNT1, CASQ1 |

*z-scores indicate directionality of biologic function, + values = activation, -values inhibition and 0 neutrality; ^†^Overlap P value as -Log, values >1.30 = p <0.05); >2.0 = p <0.01; ^‡^Number of identified genes/total number in pathway.
