# Supplementary material for: Correction: Sequential analysis of myocardial gene expression with phenotypic change: Use of cross-platform concordance to strengthen biologic relevance
Source: PLoS One. 2019 Oct 22;14(10):e0224389. doi: 10.1371/journal.pone.0224389 (PMC6804972; doi:10.1371/journal.pone.0224389)
Supplement: S2 Table — (DOCX) [file pone.0224389.s002.docx]

**S2 Table.** **Up or downregulated genes within the R and R/NR analyses, microarray or RNA-Seq measurements in the *S-R* cohort.**

| **R analyses, P <0.05** | | | | |
| --- | --- | --- | --- | --- |
| **Upregulated** | | **Downregulated** | | |
| **Microarray** | **RNA-Seq** | **Microarray** | **RNA-Seq** | |
| \| ACADL \| \| --- \| \| ADO \| \| AFAP1L2 \| \| ALAD2 \| \| ALS2CR11 \| \| AMZ2 \| \| ANKFY1 \| \| ARHGAP21 \| \| ARNT2 \| \| ATP1A1 \| \| ATP5A1 \| \| ATP6V1G2 \| \| BAG1 \| \| BAI1 \| \| BCL2A1 \| \| BHMT2 \| \| BMP2 \| \| BPHL \| \| C10orf10 \| \| C12orf69 \| \| C16orf67 \| \| C18orf12 \| \| C1orf110 \| \| C1orf77 \| \| C21orf87 \| \| C3orf20 \| \| C3orf48 \| \| C5orf28 \| \| C5orf37 \| \| C6orf123 \| \| C6orf124 \| \| C7orf11 \| \| C8orf12 \| \| C9orf29 \| \| C9orf78 \| \| CBX3 \| \| CCDC102B \| \| CCDC111 \| \| CCDC126 \| \| CCDC132 \| \| CCDC142 \| \| CCDC49 \| \| CCDC8 \| \| CD164 \| \| CD300A \| \| CECR9 \| \| CELA1 \| \| CETN2 \| \| CHRDL1 \| \| COG6 \| \| CPS1 \| \| CPSF4 \| \| CRY1 \| \| CSAD \| \| CSNK1G3 \| \| CSTB \| \| CTDSPL \| \| CXCL10 \| \| CXorf65 \| \| CYP39A1 \| \| DDHD2 \| \| DDX10 \| \| DHFRL1 \| \| DMGDH \| \| DMRT2 \| \| DPP7 \| \| EFCAB6 \| \| EFHC1 \| \| EGF \| \| EIF1AY \| \| EIF4E \| \| ERCC3 \| \| EZH1 \| \| FAM120B \| \| FAM168A \| \| FAM69A \| \| FBXO7 \| \| FCHSD2 \| \| FEZ1 \| \| FFAR2 \| \| FGF9 \| \| FIG4 \| \| FKSG29 \| \| FLJ31713 \| \| FMR1 \| \| FUT8 \| \| GAB1 \| \| GALNT11 \| \| GALNTL2 \| \| GAS2 \| \| GHRLOS \| \| GT2 \| \| GPD1L \| \| GRB10 \| \| GRID2 \| \| GSTM4 \| \| GTF2IRD2 \| \| GUSBL2 \| \| hCG_1647286 \| \| HIST1H1D \| \| HMGCLL1 \| \| HMGCS1 \| \| HPS4 \| \| HSD17B4 \| \| HSH2D \| \| HYMAI \| \| IER2 \| \| IFT88 \| \| IGSF10 \| \| INTS8 \| \| KANK1 \| \| KATL2 \| \| KCNH6 \| \| KCTD18 \| \| KDM6A \| \| KIAA0494 \| \| KIAA1919 \| \| KIR3DS1 \| \| KLHL26 \| \| KLRAQ1 \| \| KRAS \| \| KRT75 \| \| KRTAP4-4 \| \| LDB2 \| \| LMTK3 \| \| LOC100127980 \| \| LOC100128288 \| \| LOC100128439 \| \| LOC100128737 \| \| LOC100129502 \| \| LOC100130950 \| \| LOC145820 \| \| LOC146429 \| \| LOC151146 \| \| LOC153684 \| \| LOC257358 \| \| LOC284009 \| \| LOC284551 \| \| LOC339975 \| \| LOC388387 \| \| LOC390205 \| \| LOC401220 \| \| LOC595101 \| \| LOC643406 \| \| LOC646762 \| \| LOC731779 \| \| LOC731884 \| \| LTA4H \| \| LY6G6C \| \| MAP2K1 \| \| MATN4 \| \| MED23 \| \| MED28 \| \| MERTK \| \| MGC24103 \| \| MNT \| \| MRVI1 \| \| MSRB3 \| \| MTERFD2 \| \| MTHFD2 \| \| MTHFS \| \| MTRF1L \| \| MYCBP \| \| MYH3 \| \| MYO1G \| \| ALAD2 \| \| NCR00081 \| \| NHEJ1 \| \| NLRP1 \| \| NLRP4 \| \| NPR2 \| \| NTRK3 \| \| NUDT4 \| \| NUFIP2 \| \| NUP210 \| \| NUS1 \| \| OCIAD2 \| \| OR1C1 \| \| ORAI3 \| \| OTUD6B \| \| PAQR5 \| \| PARP2 \| \| PCGF6 \| \| PEG10 \| \| PGBD1 \| \| PHACTR3 \| \| PI4K2B \| \| PICALM \| \| PKD1L1 \| \| PLRG1 \| \| POLR2G \| \| PPP3R1 \| \| PPP6C \| \| PRELID2 \| \| PRG3 \| \| PRRT2 \| \| PSIP1 \| \| PSMC5 \| \| PSMF1 \| \| PTPRO \| \| RABL5 \| \| RB1CC1 \| \| RBM4B \| \| RG9MTD3 \| \| RNF160 \| \| RNF2 \| \| RPGR \| \| RPUSD2 \| \| RRP1B \| \| RSRC1 \| \| S100A7 \| \| SCAND3 \| \| SCGB2A2 \| \| SEC16B \| \| SGCE \| \| SLC10A7 \| \| SLC19A2 \| \| SLC25A16 \| \| SLC26A9 \| \| SLC35D2 \| \| SLC38A4 \| \| SLC41A1 \| \| SLC7A11 \| \| SLITRK5 \| \| SLMO2 \| \| SMARCAD1 \| \| SMU1 \| \| SP4 \| \| SPAG8 \| \| SPAG9 \| \| SPATA9 \| \| ST20 \| \| ST6GAL2 \| \| SVOPL \| \| SYCP3 \| \| TBRG1 \| \| TCEAL1 \| \| TIGD6 \| \| TLR3 \| \| TMBIM6 \| \| TMEM183A \| \| TMEM63A \| \| TNS1 \| \| TPP2 \| \| TPSAB1 \| \| TPSB2 \| \| TRIM45 \| \| TRPM2 \| \| TSFM \| \| TSPAN13 \| \| TSPYL2 \| \| TSPYL5 \| \| TSTD2 \| \| TTC6 \| \| TUBD1 \| \| UEVLD \| \| UNC13B \| \| VKORC1L1 \| \| WDR36 \| \| XPA \| \| YES1 \| \| YTHDF1 \| \| ZBTB39 \| \| ZCWPW1 \| \| ZDHHC6 \| \| ZNF135 \| \| ZNF142 \| \| ZNF193 \| \| ZNF264 \| \| ZNF280C \| \| ZNF404 \| \| ZNF468 \| \| ZNF558 \| \| ZNF578 \| \| ZNF649 \| \| ZNF713 \| \| ZNF732 \| \| ZNF767 \| \| ZNF92 \| \| ZSCAN4 \| | \| A1BG \| \| --- \| \| ABCA8 \| \| ACAA2 \| \| ACAD10 \| \| ACADVL \| \| ACAT1 \| \| ACSL1 \| \| ACSM5 \| \| ACSS2 \| \| ADAM11 \| \| ADAM33 \| \| ADAMTS15 \| \| ADAMTS7 \| \| ADCK4 \| \| ADCY5 \| \| ADCYAP1R1 \| \| ADD3 \| \| ADH1B \| \| ADRB1 \| \| AGBL2 \| \| AGTR1 \| \| ALDH2 \| \| ALDOC \| \| ANK1 \| \| ANKRD44 \| \| ANKZF1 \| \| AQP7 \| \| ARHGAP10 \| \| ARSI \| \| ART3 \| \| ART5 \| \| ASB1 \| \| ASB10 \| \| ASB14 \| \| ASB16 \| \| ASB8 \| \| ASPSCR1 \| \| ATP2A2 \| \| ATP5D \| \| ATP5G2 \| \| ATP5I \| \| ATP8B4 \| \| AURKAIP1 \| \| AUTS2 \| \| BAIAP2L2 \| \| BCAR3 \| \| BCKDHA \| \| BCL2L12 \| \| BCL6 \| \| BCL7A \| \| BCL7C \| \| BEND5 \| \| BLOC1S1 \| \| BLVRB \| \| BMP7 \| \| BPHL \| \| BRSK2 \| \| BTN1A1 \| \| BTN3A1 \| \| C10orf11 \| \| C10orf116 \| \| C10orf128 \| \| C10orf58 \| \| C11orf67 \| \| C12orf33 \| \| C12orf57 \| \| C13orf30 \| \| C14orf159 \| \| C15orf33 \| \| C15orf38 \| \| C19orf70 \| \| C1orf105 \| \| C1orf151 \| \| C1orf204 \| \| C1orf95 \| \| C1QL1 \| \| C20orf7 \| \| C21orf49 \| \| C21orf90 \| \| C2orf71 \| \| C3 \| \| C3orf37 \| \| C3orf43 \| \| C3orf45 \| \| C3orf55 \| \| C5orf54 \| \| C6orf130 \| \| C6orf57 \| \| C7orf10 \| \| C7orf63 \| \| C7orf70 \| \| C8orf34 \| \| C8orf40 \| \| CA1 \| \| CA14 \| \| CA4 \| \| CAB39L \| \| CAC2D4 \| \| CAPS2 \| \| CATSPERB \| \| CCDC101 \| \| CCDC17 \| \| CCDC28B \| \| CCDC39 \| \| CCDC88C \| \| CCR9 \| \| CCRN4L \| \| CD52 \| \| CD5L \| \| CD7 \| \| CD8A \| \| CD96 \| \| CDH13 \| \| CDH26 \| \| CDYL \| \| CECR5 \| \| CEL \| \| CENPV \| \| CEP192 \| \| CEP68 \| \| CFD \| \| CFLAR \| \| CHADL \| \| CHCHD10 \| \| CHDH \| \| CHI3L1 \| \| CHPT1 \| \| CHRM2 \| \| CKM \| \| CNBP \| \| CNTFR \| \| COL28A1 \| \| COPS5 \| \| COPS6 \| \| COQ10A \| \| COQ4 \| \| COQ9 \| \| CORIN \| \| COX19 \| \| COX4I1 \| \| COX5A \| \| COX5B \| \| COX6A2 \| \| COX6B1 \| \| COX7A1 \| \| COX8A \| \| CPA3 \| \| CPO \| \| CPT1B \| \| CREB3L4 \| \| CRYGS \| \| CTAGE5 \| \| CTNNBIP1 \| \| CTNNBL1 \| \| CTSG \| \| CTSW \| \| CXCR6 \| \| CXXC1 \| \| CYC1 \| \| CYP4Z1 \| \| DEDD \| \| DENND2D \| \| DHFRL1 \| \| DHRS12 \| \| DHRS7C \| \| DIO3OS \| \| DIXDC1 \| \| DJA3 \| \| DOCK10 \| \| DPF2 \| \| DSCAML1 \| \| DTX2 \| \| DYRK2 \| \| ECD \| \| ECH1 \| \| ECHDC3 \| \| EDA \| \| EEPD1 \| \| EFHC2 \| \| EGFLAM \| \| EID2B \| \| ENTPD3 \| \| ENTPD6 \| \| EPB41L2 \| \| EPC1 \| \| EPDR1 \| \| ERC1 \| \| ESPN \| \| ESRP2 \| \| ETFB \| \| EXOSC3 \| \| EXOSC5 \| \| EXPH5 \| \| FADD \| \| FAM123B \| \| FAM124A \| \| FAM175A \| \| FAM179A \| \| FAM46A \| \| FAM46C \| \| FAM65C \| \| FAM78A \| \| FAM81A \| \| FAM84A \| \| FAU \| \| FBXO46 \| \| FDFT1 \| \| FEZ1 \| \| FGF12 \| \| FGFBP2 \| \| FIGN \| \| FITM1 \| \| FLCN \| \| FLRT1 \| \| FLT3LG \| \| FMO2 \| \| FNBP4 \| \| FNDC5 \| \| FOXN3 \| \| FUNDC2 \| \| FXYD2 \| \| G0S2 \| \| GABBR2 \| \| GALNTL1 \| \| GBX1 \| \| GCOM1 \| \| GIMAP1 \| \| GIMAP5 \| \| GIMAP7 \| \| GLTSCR2 \| \| GLYCTK \| \| GPD1 \| \| GPIHBP1 \| \| GPR37L1 \| \| GPSM1 \| \| GREB1 \| \| GRM2 \| \| GSTM2 \| \| GSTM5 \| \| GSTP1 \| \| GTF3C5 \| \| GZMK \| \| GZMM \| \| HACL1 \| \| HADHA \| \| HADHB \| \| HEATR2 \| \| HERPUD1 \| \| HEY2 \| \| HIBADH \| \| HIPK3 \| \| HIPK4 \| \| HIRIP3 \| \| HIST1H1C \| \| HIST1H2BF \| \| HIST3H2A \| \| HLF \| \| HMGCS2 \| \| HOMER2 \| \| HSD17B3 \| \| HSDL2 \| \| HSPB2 \| \| HSPB3 \| \| HTRA2 \| \| IDH2 \| \| IDH3B \| \| IFNK \| \| IFRD2 \| \| IGDCC4 \| \| IGHG1 \| \| IGKC \| \| IKZF3 \| \| IL18RAP \| \| ILKAP \| \| ILVBL \| \| IMPA2 \| \| INTS9 \| \| ISLR2 \| \| ITGA8 \| \| ITGB8 \| \| ITIH4 \| \| ITPRIPL1 \| \| JARID2 \| \| JHDM1D \| \| KANK1 \| \| KC2 \| \| KC6 \| \| KCND3 \| \| KCNH8 \| \| KCNIP2 \| \| KCNIP4 \| \| KCNJ11 \| \| KCNMA1 \| \| KIAA0408 \| \| KIF22 \| \| KLF9 \| \| KLHDC3 \| \| KLHL21 \| \| KLHL22 \| \| KLHL24 \| \| KLHL7 \| \| KLKB1 \| \| KLRF1 \| \| KLRK1 \| \| LAMC2 \| \| LCNL1 \| \| LDB2 \| \| LGI4 \| \| LGR6 \| \| LNX1 \| \| LPAR3 \| \| LPAR5 \| \| LPCAT4 \| \| LRRC16B \| \| LRRC39 \| \| LRRN3 \| \| LSS \| \| LYRM1 \| \| MACROD2 \| \| MAL \| \| MAML2 \| \| MAP3K5 \| \| MAPKAPK3 \| \| MARVELD3 \| \| MAX \| \| MCOLN1 \| \| MED13L \| \| MESP1 \| \| METTL7B \| \| MFNG \| \| MFSD11 \| \| MLLT10 \| \| MLLT6 \| \| MLPH \| \| MMP11 \| \| MPP1 \| \| MPP3 \| \| MRO \| \| MRPL2 \| \| MRPL21 \| \| MRPL23 \| \| MRPL24 \| \| MRPL38 \| \| MRPL43 \| \| MRPL48 \| \| MRPL51 \| \| MRPS15 \| \| MRPS24 \| \| MRPS25 \| \| MS4A1 \| \| MT1X \| \| MT3 \| \| MTIF3 \| \| MTSS1 \| \| MYCL1 \| \| MYL3 \| \| MYLK4 \| \| MYOF \| \| N6AMT1 \| \| RS2 \| \| NCAM2 \| \| NCKAP5 \| \| NCR00161 \| \| NDUFA13 \| \| NDUFB10 \| \| NDUFB11 \| \| NDUFB3 \| \| NDUFB7 \| \| NDUFB9 \| \| NDUFC2 \| \| NDUFS2 \| \| NDUFS3 \| \| NDUFS6 \| \| NDUFS7 \| \| NDUFS8 \| \| NFXL1 \| \| NPC1 \| \| NPHP3 \| \| NR3C2 \| \| NRXN1 \| \| NUDT13 \| \| NUDT4 \| \| NUP35 \| \| NXPH3 \| \| OSBPL1A \| \| OSBPL5 \| \| P2RY1 \| \| PAN2 \| \| PANK4 \| \| PCDHGA10 \| \| PCDHGA11 \| \| PCDHGB6 \| \| PCDHGB7 \| \| PCDHGB8P \| \| PCMTD2 \| \| PDE2A \| \| PDE3A \| \| PDE7B \| \| PDIK1L \| \| PEBP4 \| \| PEX11A \| \| PFKFB3 \| \| PGAM2 \| \| PHACTR3 \| \| PHF15 \| \| PHKG1 \| \| PHPT1 \| \| PID1 \| \| PIGL \| \| PIM3 \| \| PKD1L1 \| \| PKP2 \| \| PLA2G4F \| \| PLAG1 \| \| PLCL2 \| \| PLD1 \| \| PLD6 \| \| PLEKHF1 \| \| PLEKHG4B \| \| PLXNB1 \| \| POLE \| \| POLM \| \| POLR2I \| \| POLR2L \| \| PPAPDC3 \| \| PPARGC1B \| \| PPM1L \| \| PPP1R1A \| \| PQBP1 \| \| PRCD \| \| PRDX1 \| \| PRDX5 \| \| PRELID2 \| \| PRF1 \| \| PRKCE \| \| PRODH \| \| PRSS36 \| \| PSMB10 \| \| PSMB3 \| \| PSMB6 \| \| PTCH1 \| \| PTGDS \| \| PTPRCAP \| \| PWWP2A \| \| PXMP2 \| \| PYHIN1 \| \| PZP \| \| RAB40B \| \| RAF1 \| \| RAGE \| \| RBM17 \| \| RBM28 \| \| RCOR2 \| \| RDH14 \| \| RET \| \| REV1 \| \| RG9MTD3 \| \| RGMA \| \| RHAG \| \| RIMS1 \| \| RMST \| \| RNF148 \| \| RNF165 \| \| RNF187 \| \| RNF207 \| \| RNLS \| \| ROR2 \| \| RORC \| \| RPL10 \| \| RPL14 \| \| RPL18 \| \| RPL19 \| \| RPL22 \| \| RPL24 \| \| RPL27A \| \| RPL34 \| \| RPL35 \| \| RPL36 \| \| RPL37A \| \| RPL38 \| \| RPL5 \| \| RPLP0 \| \| RPS10 \| \| RPS11 \| \| RPS14 \| \| RPS21 \| \| RPS23 \| \| RPS24 \| \| RPS27A \| \| RPS28 \| \| RPS3 \| \| RPS4X \| \| RPS5 \| \| RPS8 \| \| RPUSD3 \| \| RSAD1 \| \| RUFY1 \| \| RUNX3 \| \| S100A1 \| \| S1PR3 \| \| SCN1A \| \| SCUBE3 \| \| SDHA \| \| SDK1 \| \| SEC14L5 \| \| SEC16B \| \| SELENBP1 \| \| SEMA6D \| \| SEPP1 \| \| SGSM1 \| \| SH2D1B \| \| SH3KBP1 \| \| SH3RF2 \| \| SHISA3 \| \| SIL1 \| \| SIRT4 \| \| SLC10A1 \| \| SLC13A3 \| \| SLC25A26 \| \| SLC26A1 \| \| SLC26A9 \| \| SLC27A1 \| \| SLC29A1 \| \| SLC29A2 \| \| SLC2A9 \| \| SLC5A1 \| \| SLCO3A1 \| \| SLIT2 \| \| SLIT3 \| \| SMARCD3 \| \| SMTNL2 \| \| SI3 \| \| SPC5 \| \| SNHG6 \| \| SNRNP25 \| \| SNRNP35 \| \| SNRNP70 \| \| SOX10 \| \| SOX15 \| \| SPAG7 \| \| SPATA5L1 \| \| SPATC1 \| \| SPHK2 \| \| SPHKAP \| \| STAG1 \| \| STARD10 \| \| STARD3 \| \| STEAP4 \| \| STOML2 \| \| STXBP6 \| \| SUMF1 \| \| SUSD4 \| \| SYCP3 \| \| TAS2R5 \| \| TBX6 \| \| TC2N \| \| TCEA3 \| \| TCF15 \| \| TESC \| \| THAP1 \| \| THAP4 \| \| THNSL1 \| \| TIFA \| \| TIGD1 \| \| TIMP3 \| \| TJP2 \| \| TKT \| \| TKTL1 \| \| TMCO7 \| \| TMEM121 \| \| TMEM132B \| \| TMEM132C \| \| TMEM143 \| \| TMEM208 \| \| TMEM220 \| \| TMEM27 \| \| TMEM85 \| \| TMOD2 \| \| TMTC1 \| \| TOX \| \| TPCN1 \| \| TPSAB1 \| \| TRABD \| \| TRAC \| \| TRAP1 \| \| TRIM16 \| \| TRIM16L \| \| TRIM4 \| \| TRIM7 \| \| TRPM7 \| \| TSPAN7 \| \| TXNDC15 \| \| UBAP2 \| \| UBXN1 \| \| UCKL1 \| \| UHRF2 \| \| UPF3A \| \| UQCRC1 \| \| VEZF1 \| \| VIPR1 \| \| VIT \| \| VPREB3 \| \| VWA3A \| \| WDR62 \| \| WDSUB1 \| \| WNT5A \| \| WNT5B \| \| WSCD2 \| \| ZACN \| \| ZBTB40 \| \| ZCCHC11 \| \| ZFP2 \| \| ZNF135 \| \| ZNF225 \| \| ZNF33A \| \| ZNF33B \| \| ZNF418 \| \| ZNF497 \| \| ZNF502 \| \| ZNF521 \| \| ZNF540 \| \| ZNF567 \| \| ZNF674 \| \| ZNF74 \| \| ZNF784 \| \| ZNF831 \| \| ZNF837 \| \| ZNF843 \| \| ZNHIT3 \| \| ZXDC \| \| 42805 (MARCHF11)* \| | \| ACRBP \| \| --- \| \| ACY3 \| \| ADAMTS4 \| \| ADRBK1 \| \| ADSS \| \| AFFX-DapX-3_at \| \| AFG3L1 \| \| AFMID \| \| AKAP5 \| \| ANGPT4 \| \| APOA2 \| \| APOA4 \| \| ARL3 \| \| ARPC4 \| \| ATP13A4 \| \| AURKC \| \| AZI1 \| \| AZU1 \| \| BCORL1 \| \| BIK \| \| BIRC7 \| \| BMP10 \| \| BPIL1 \| \| BPNT1 \| \| BRDT \| \| BTBD17 \| \| C15orf55 \| \| C16orf74 \| \| C17orf52 \| \| C18orf56 \| \| C19orf33 \| \| C19orf41 \| \| C19orf50 \| \| C19orf75 \| \| C20orf141 \| \| C21orf93 \| \| C22orf45 \| \| C3orf16 \| \| C7orf33 \| \| C8orf71 \| \| CACNG2 \| \| CAPZA1 \| \| CBLC \| \| CCDC129 \| \| CCDC144A \| \| CCDC15 \| \| CCDC37 \| \| CCL17 \| \| CCL8 \| \| CD300LB \| \| CD5 \| \| CDC37L1 \| \| CDH15 \| \| CEACAM3 \| \| CENPH \| \| CFB \| \| CFTR \| \| CHN1 \| \| CHRNB2 \| \| CLP1 \| \| CMTM2 \| \| COL22A1 \| \| COX10 \| \| COX6A1 \| \| CPT1B \| \| CRHR1 \| \| CST9 \| \| CSTF1 \| \| CTXN3 \| \| CXCL5 \| \| CYB5R4 \| \| CYP27C1 \| \| CYP2A7P1 \| \| CYP2C19 \| \| CYP2D6 \| \| CYP51A1 \| \| DAB2IP \| \| DAPP1 \| \| DEFB123 \| \| DHX34 \| \| DIAPH1 \| \| DIO1 \| \| DKFZP586I1420 \| \| DNM1P41 \| \| DUX1 \| \| EF2 \| \| EGLN3 \| \| EIF2C1 \| \| EIF3H \| \| EIF4G3 \| \| ELL \| \| EPHA1 \| \| ESR1 \| \| EVPLL \| \| EXOC4 \| \| FAM188B \| \| FAM71A \| \| FAM71C \| \| FGF20 \| \| FGF6 \| \| FLJ16686 \| \| FLJ39080 \| \| FLJ44054 \| \| FMO9P \| \| FOXD4 \| \| FOXK1 \| \| FPR2 \| \| FUCA2 \| \| GAS7 \| \| GGA2 \| \| GLP2R \| \| GPR108 \| \| GPR114 \| \| GRIN1 \| \| GSTTP1 \| \| HAPLN2 \| \| hCG_1643808 \| \| hCG_1776007 \| \| HDHD1A \| \| HEPACAM2 \| \| HGFAC \| \| HIST1H4D \| \| HIST1H4G \| \| HMGB2 \| \| HOXD12 \| \| HSPB8 \| \| HTR3A \| \| IFNW1 \| \| IGFN1 \| \| IL25 \| \| IL9 \| \| ILDR1 \| \| INSL4 \| \| IPO9 \| \| IRAK4 \| \| IRF4 \| \| KCNN1 \| \| KCTD9 \| \| KIAA0746 \| \| KIAA1543 \| \| KIAA1614 \| \| KLC3 \| \| KLF16 \| \| KP1 \| \| KRT23 \| \| KRT27 \| \| KRTAP5-8 \| \| LBX1 \| \| LILRP2 \| \| LMTK2 \| \| LMX1B \| \| LOC100128098 \| \| LOC100128751 \| \| LOC100128844 \| \| LOC100130456 \| \| LOC144742 \| \| LOC284080 \| \| LOC286184 \| \| LOC286370 \| \| LOC338588 \| \| LOC339822 \| \| LOC388588 \| \| LOC440792 \| \| LOC51145 \| \| LOC554201 \| \| LOC55908 \| \| LOC641518 \| \| LOC728475 \| \| LOXHD1 \| \| LRMP \| \| LRRC38 \| \| LUZP1 \| \| MAGEA10 \| \| MBD2 \| \| MCM2 \| \| MEP1A \| \| MESP1 \| \| MEX3D \| \| MGC34800 \| \| MMP17 \| \| MMP25 \| \| MPZL2 \| \| MSI1 \| \| MSR1 \| \| NP \| \| NCOR2 \| \| NCR00162 \| \| NCR00176 \| \| NCR00185 \| \| NFAM1 \| \| NFKBIL1 \| \| NLRP13 \| \| NMBR \| \| NOL6 \| \| NPBWR1 \| \| NQO1 \| \| NR4A1 \| \| ODF3L1 \| \| OR1A2 \| \| OR3A3 \| \| OR8B8 \| \| OR8G1 \| \| OTOP2 \| \| PAFAH1B2 \| \| PALM3 \| \| PCDHB7 \| \| PDE9A \| \| PGLYRP1 \| \| PI3 \| \| PLAC1L \| \| PLD4 \| \| PLXNB1 \| \| PMAIP1 \| \| PPPDE1 \| \| PRAME \| \| PRKCA \| \| PRKRIP1 \| \| PRM1 \| \| PRM2 \| \| PRODH2 \| \| PSKH2 \| \| PYGO2 \| \| RANBP1 \| \| RGAG4 \| \| RHBDD1 \| \| ROBO3 \| \| RP13-102H20.1 \| \| RQCD1 \| \| RSPH4A \| \| RWDD2A \| \| S1PR2 \| \| SAMD14 \| \| SAMD8 \| \| SAPS3 \| \| SCAND2 \| \| SDS \| \| SELI \| \| SEMA4D \| \| SIGLEC7 \| \| SIPA1L3 \| \| SLC13A2 \| \| SLC17A1 \| \| SLC22A7 \| \| SLC44A4 \| \| SLC6A19 \| \| SLFNL1 \| \| SMAD2 \| \| SNX22 \| \| SOX14 \| \| SPATA21 \| \| SPATS2 \| \| SPDEF \| \| SPERT \| \| SPIB \| \| SRP68 \| \| STAG3L4 \| \| STK32A \| \| SYCE1 \| \| SYT3 \| \| TEKT5 \| \| TEX19 \| \| TEX28 \| \| TFDP2 \| \| TFR2 \| \| TGFB2 \| \| TGM6 \| \| TLR8 \| \| TM7SF4 \| \| TMEM165 \| \| TNF \| \| TNP1 \| \| TRBV10-2 \| \| TRBV25-1 \| \| TRBV7-3 \| \| TREX2 \| \| TRPC7 \| \| TRPM1 \| \| TRPV4 \| \| TSXIP1 \| \| TSPAN17 \| \| TSSK1B \| \| TXNDC2 \| \| USF1 \| \| USP29 \| \| USP9Y \| \| UTS2R \| \| VPS36 \| \| VTI1B \| \| WDR69 \| \| WNT4 \| \| ZBP1 \| \| ZG16B \| \| ZIC5 \| \| ZNF77 \| \|  \| | \| AASS \| \| \| --- \| --- \| \| AATF \| \| \| ABAT \| \| \| ABCA1 \| \| \| ABCG4 \| \| \| ABHD2 \| \| \| ABHD5 \| \| \| ABI2 \| \| \| ACAD11 \| \| \| ACBD3 \| \| \| ACE2 \| \| \| ACOT9 \| \| \| ACOX3 \| \| \| ACP2 \| \| \| ACRC \| \| \| ACSL4 \| \| \| ACTN1 \| \| \| ACTN2 \| \| \| ACTR2 \| \| \| ADAM12 \| \| \| ADAM19 \| \| \| ADAMTS14 \| \| \| ADAMTS2 \| \| \| ADAT1 \| \| \| ADC \| \| \| ADCY6 \| \| \| ADRA1D \| \| \| AFF4 \| \| \| AGFG1 \| \| \| AGTRAP \| \| \| AGXT2L1 \| \| \| AKAP11 \| \| \| AKAP13 \| \| \| AKIRIN1 \| \| \| ALDH3A2 \| \| \| ALPK2 \| \| \| ALPK3 \| \| \| ALS2 \| \| \| ALX3 \| \| \| AMMECR1 \| \| \| AMOTL1 \| \| \| ANKIB1 \| \| \| ANKRD10 \| \| \| ANKRD17 \| \| \| ANKRD18A \| \| \| ANKRD34C \| \| \| ANXA11 \| \| \| ANXA7 \| \| \| AP1G1 \| \| \| AP2B1 \| \| \| AP3M1 \| \| \| AP4E1 \| \| \| AP4S1 \| \| \| APLN \| \| \| APLP1 \| \| \| APLP2 \| \| \| APOA1 \| \| \| APOL4 \| \| \| APP \| \| \| APTX \| \| \| AQP10 \| \| \| ARAP3 \| \| \| ARF4 \| \| \| ARHGAP1 \| \| \| ARHGAP11A \| \| \| ARHGAP5 \| \| \| ARHGEF12 \| \| \| ARHGEF7 \| \| \| ARHGEF9 \| \| \| ARID5B \| \| \| ARIH1 \| \| \| ARL6IP5 \| \| \| ARMCX3 \| \| \| ARPC5 \| \| \| ARSD \| \| \| ARSE \| \| \| ASAP1 \| \| \| ASCC3 \| \| \| ASNS \| \| \| ASPM \| \| \| ASPN \| \| \| ATF5 \| \| \| ATF6 \| \| \| ATF7IP2 \| \| \| ATP13A3 \| \| \| ATP1A1 \| \| \| ATP1B1 \| \| \| ATP2B4 \| \| \| ATP2C1 \| \| \| ATP6V1B2 \| \| \| ATP6V1E2 \| \| \| ATP8B1 \| \| \| ATP8B3 \| \| \| ATPAF1 \| \| \| ATRNL1 \| \| \| ATRX \| \| \| AZIN1 \| \| \| B3GALNT2 \| \| \| B3GNT2 \| \| \| B4GALT4 \| \| \| BAG3 \| \| \| BAZ1A \| \| \| BCL2 \| \| \| BCL2L14 \| \| \| BCL2L2 \| \| \| BECN1 \| \| \| BEND3 \| \| \| BEX1 \| \| \| BICD2 \| \| \| BMPR2 \| \| \| BMS1 \| \| \| BNIP2 \| \| \| BRAF \| \| \| BRCC3 \| \| \| BRE \| \| \| BRIP1 \| \| \| BTBD1 \| \| \| BTBD3 \| \| \| BUB1 \| \| \| BUB1B \| \| \| BUD31 \| \| \| BVES \| \| \| C10orf118 \| \| \| C10orf93 \| \| \| C11orf24 \| \| \| C11orf80 \| \| \| C12orf23 \| \| \| C12orf43 \| \| \| C12orf48 \| \| \| C12orf49 \| \| \| C14orf119 \| \| \| C14orf129 \| \| \| C14orf132 \| \| \| C14orf135 \| \| \| C14orf37 \| \| \| C15orf23 \| \| \| C15orf42 \| \| \| C15orf44 \| \| \| C16orf3 \| \| \| C17orf85 \| \| \| C18orf45 \| \| \| C18orf8 \| \| \| C19orf63 \| \| \| C1GALT1C1 \| \| \| C1orf135 \| \| \| C1orf190 \| \| \| C1orf21 \| \| \| C1orf96 \| \| \| C1QTNF6 \| \| \| C20orf108 \| \| \| C20orf194 \| \| \| C21orf7 \| \| \| C2orf67 \| \| \| C2orf81 \| \| \| C3orf14 \| \| \| C3orf58 \| \| \| C5orf46 \| \| \| C7orf41 \| \| \| C7orf60 \| \| \| C8orf33 \| \| \| C8orf51 \| \| \| C9orf16 \| \| \| C9orf30 \| \| \| CALD1 \| \| \| CALU \| \| \| CAPRIN1 \| \| \| CASP3 \| \| \| CASQ2 \| \| \| CCDC113 \| \| \| CCDC25 \| \| \| CCDC80 \| \| \| CCNB2 \| \| \| CCND2 \| \| \| CCNE1 \| \| \| CCNG2 \| \| \| CD151 \| \| \| CD55 \| \| \| CD59 \| \| \| CDC14B \| \| \| CDC20 \| \| \| CDC25A \| \| \| CDC27 \| \| \| CDCA8 \| \| \| CDH2 \| \| \| CDK2AP1 \| \| \| CDK8 \| \| \| CDKN2B \| \| \| CENPA \| \| \| CENPF \| \| \| CENPN \| \| \| CETP \| \| \| CHMP4B \| \| \| CHPF2 \| \| \| CHRM5 \| \| \| CHST15 \| \| \| CKAP2 \| \| \| CKAP2L \| \| \| CKAP4 \| \| \| CKAP5 \| \| \| CLCN3 \| \| \| CLIP1 \| \| \| CLTCL1 \| \| \| CMAS \| \| \| CMTM4 \| \| \| CMYA5 \| \| \| CNDP2 \| \| \| CNKSR3 \| \| \| CNN1 \| \| \| COBLL1 \| \| \| COL16A1 \| \| \| COL1A1 \| \| \| COL1A2 \| \| \| COL21A1 \| \| \| COL23A1 \| \| \| COL3A1 \| \| \| COL4A1 \| \| \| COL5A1 \| \| \| COL5A2 \| \| \| COL8A1 \| \| \| COPA \| \| \| COPB2 \| \| \| COX15 \| \| \| CPVL \| \| \| CREG1 \| \| \| CRELD1 \| \| \| CREM \| \| \| CRK \| \| \| CRKL \| \| \| CRLF1 \| \| \| CRY1 \| \| \| CRYBG3 \| \| \| CRYZ \| \| \| CSDE1 \| \| \| CSPG4 \| \| \| CSRP2 \| \| \| CTBS \| \| \| CTGF \| \| \| CTSB \| \| \| CTSZ \| \| \| CUX1 \| \| \| CXCL5 \| \| \| CXCR7 \| \| \| CXorf36 \| \| \| CYB5R1 \| \| \| CYLD \| \| \| CYP11A1 \| \| \| CYP19A1 \| \| \| CYP2U1 \| \| \| DACT3 \| \| \| DARS2 \| \| \| DAZAP2 \| \| \| DBN1 \| \| \| DCAF6 \| \| \| DCBLD2 \| \| \| DCTN1 \| \| \| DCTN5 \| \| \| DDAH1 \| \| \| DDB2 \| \| \| DDO \| \| \| DDX24 \| \| \| DDX3X \| \| \| DDX3Y \| \| \| DDX60L \| \| \| DENND5A \| \| \| DENND5B \| \| \| DES \| \| \| DF5 \| \| \| DHX8 \| \| \| DLG1 \| \| \| DLK2 \| \| \| DH1 \| \| \| DJA4 \| \| \| DJB4 \| \| \| DJB5 \| \| \| DJC13 \| \| \| DJC15 \| \| \| DJC21 \| \| \| DNMT1 \| \| \| DOK4 \| \| \| DPP8 \| \| \| DPT \| \| \| DSEL \| \| \| DTL \| \| \| DTX4 \| \| \| DUSP27 \| \| \| DUSP3 \| \| \| DUSP5 \| \| \| DUSP6 \| \| \| DYNC1LI1 \| \| \| DYNC1LI2 \| \| \| DYNLT1 \| \| \| DYX1C1 \| \| \| E2F1 \| \| \| E2F3 \| \| \| ECT2 \| \| \| EDA2R \| \| \| EFR3A \| \| \| EFR3B \| \| \| EGLN3 \| \| \| EIF2C1 \| \| \| EIF2S3 \| \| \| EIF4E \| \| \| EIF4E3 \| \| \| EIF5A2 \| \| \| ELF1 \| \| \| ELOVL5 \| \| \| ELTD1 \| \| \| EML2 \| \| \| EMP1 \| \| \| EH \| \| \| EM \| \| \| ENC1 \| \| \| ENO3 \| \| \| ENOX2 \| \| \| ENTPD4 \| \| \| ERC2 \| \| \| ERMP1 \| \| \| ETF1 \| \| \| EVI5 \| \| \| EXO1 \| \| \| EXOC6B \| \| \| EXT1 \| \| \| EXTL3 \| \| \| EYA3 \| \| \| EYA4 \| \| \| EZR \| \| \| CC2 \| \| F2R \| \| F2RL2 \| \| F3 \| \| FAM104A \| \| FAM109B \| \| FAM110B \| \| FAM114A1 \| \| FAM114A2 \| \| FAM120A \| \| FAM122B \| \| FAM160B1 \| \| FAM169A \| \| FAM177A1 \| \| FAM49B \| \| FAM50A \| \| FAM69A \| \| FAM82A2 \| \| FAM83G \| \| FAP \| \| FARP1 \| \| FATE1 \| \| FBN1 \| \| FBXL20 \| \| FBXO27 \| \| FBXO30 \| \| FBXO9 \| \| FBXW11 \| \| FEM1C \| \| FEZ2 \| \| FGD6 \| \| FGFR1 \| \| FHAD1 \| \| FILIP1 \| \| FILIP1L \| \| FKBP15 \| \| FMN1 \| \| FN1 \| \| FNDC3B \| \| FOXS1 \| \| FRRS1 \| \| FSD1L \| \| FSTL3 \| \| FUCA2 \| \| FUT11 \| \| FZD8 \| \| G3BP2 \| \| GABARAPL2 \| \| GADD45A \| \| GALNT10 \| \| GALNT5 \| \| GAN \| \| GARNL3 \| \| GCA \| \| GDF11 \| \| GEMIN8 \| \| GGPS1 \| \| GINS2 \| \| GINS4 \| \| GLG1 \| \| GLS \| \| GLT1D1 \| \| GLT25D1 \| \| GLT8D2 \| \| GLTP \| \| GLUD1 \| \| GNB5 \| \| GNG12 \| \| GNS \| \| GOLGA2 \| \| GOLGA3 \| \| GOLGA4 \| \| GOLIM4 \| \| GOPC \| \| GPA33 \| \| GPC1 \| \| GPR107 \| \| GPR137B \| \| GPR137C \| \| GPR173 \| \| GPR176 \| \| GPR27 \| \| GPR63 \| \| GPR85 \| \| GSG2 \| \| GTF3C1 \| \| GUF1 \| \| GYG1 \| \| GYS1 \| \| H19 \| \| H2AFV \| \| H2AFX \| \| HAPLN1 \| \| HDAC2 \| \| HECA \| \| HEPH \| \| HERC4 \| \| HERC5 \| \| HEXA \| \| HEXB \| \| HIATL1 \| \| HIF1AN \| \| HIP1R \| \| HIPK1 \| \| HIPK2 \| \| HIST1H1A \| \| HIST1H2AL \| \| HK1 \| \| HMGCR \| \| HMGN4 \| \| HMMR \| \| HN1 \| \| HRAS \| \| HRC \| \| HS2ST1 \| \| HSP90AA1 \| \| HSPA4L \| \| HTATSF1 \| \| HTRA1 \| \| HYAL3 \| \| ICMT \| \| IFR1 \| \| IGF2BP2 \| \| IGFBP2 \| \| IKBKAP \| \| IL10RB \| \| IL17D \| \| IL1R1 \| \| IMPA1 \| \| IMPAD1 \| \| INPP4B \| \| INPP5A \| \| INPP5F \| \| INTS2 \| \| INTS3 \| \| INTS8 \| \| IPO5 \| \| IPPK \| \| IQCD \| \| IQGAP3 \| \| IREB2 \| \| IRGQ \| \| ITGA7 \| \| ITGAE \| \| ITGAV \| \| ITGB1 \| \| ITPR1 \| \| ITPRIPL2 \| \| ITSN1 \| \| JAK2 \| \| JAZF1 \| \| JMY \| \| JUNB \| \| KATL1 \| \| KBTBD10 \| \| KCB1 \| \| KCMF1 \| \| KCNC4 \| \| KCNJ4 \| \| KCNJ5 \| \| KCNK6 \| \| KCNMB1 \| \| KCNQ4 \| \| KCTD2 \| \| KCTD9 \| \| KDELC1 \| \| KDELR3 \| \| KIAA0100 \| \| KIAA0556 \| \| KIAA1161 \| \| KIAA1191 \| \| KIAA1279 \| \| KIAA1539 \| \| KIAA1826 \| \| KIDINS220 \| \| KIF13A \| \| KIF1A \| \| KIF20A \| \| KIF23 \| \| KIF3B \| \| KIRREL \| \| KLHL12 \| \| KLHL2 \| \| KLHL34 \| \| KLHL6 \| \| KP1 \| \| KP4 \| \| KP6 \| \| KPNB1 \| \| KTN1 \| \| LAMA2 \| \| LARP4 \| \| LCLAT1 \| \| LCN12 \| \| LEF1 \| \| LEPREL1 \| \| LHFPL2 \| \| LIG4 \| \| LIMCH1 \| \| LNP1 \| \| LNX2 \| \| LOX \| \| LOXL1 \| \| LOXL2 \| \| LOXL4 \| \| LPCAT3 \| \| LPIN1 \| \| LRCH1 \| \| LRRC41 \| \| LRRC59 \| \| LRRFIP1 \| \| LSM1 \| \| LTBP1 \| \| LTBP2 \| \| LUM \| \| LUZP1 \| \| MACF1 \| \| MAFK \| \| MAGED1 \| \| MAGI3 \| \| MAOA \| \| MAP1A \| \| MAP1B \| \| MAP1LC3B \| \| MAP2K5 \| \| MAP3K3 \| \| MAP4 \| \| MAP4K4 \| \| MAP7 \| \| MAP9 \| \| MAPK1 \| \| MAPK1IP1L \| \| MAPK8IP1 \| \| MAPRE2 \| \| MARS \| \| MARS2 \| \| MASTL \| \| MBTPS1 \| \| MCAM \| \| MCL1 \| \| MCM10 \| \| MCM6 \| \| MDFIC \| \| MED12L \| \| MED13 \| \| MED19 \| \| MED28 \| \| MED7 \| \| MEF2A \| \| MEMO1 \| \| MEOX1 \| \| MEST \| \| MFAP2 \| \| MFGE8 \| \| MFI2 \| \| MGA \| \| MGAT5 \| \| MGP \| \| MI \| \| MIB1 \| \| MICALCL \| \| MKI67 \| \| MKRN2 \| \| MEF2A \| \| MEMO1 \| \| MEOX1 \| \| MEST \| \| MFAP2 \| \| MFGE8 \| \| MFI2 \| \| MGA \| \| MGAT5 \| \| MGP \| \| MI \| \| MIB1 \| \| MICALCL \| \| MKI67 \| \| MKRN2 \| \|  \| \|  \| \|  \| \|  \| \|  \| \|  \| \|  \| \|  \| \|  \| \|  \| \|  \| \|  \| \|  \| \|  \| \|  \| \|  \| \|  \| \|  \| \|  \| \|  \| \|  \| \|  \| \|  \| \|  \| \|  \| \|  \| \|  \| | \| MLA \| \| --- \| \| MLLT11 \| \| MMGT1 \| \| MMP14 \| \| MMP2 \| \| MOXD1 \| \| MPV17 \| \| MPV17L2 \| \| MPZL3 \| \| MSRA \| \| MTMR2 \| \| MTMR9 \| \| MTOR \| \| MUSTN1 \| \| MXRA5 \| \| MXRA7 \| \| MYEF2 \| \| MYL6 \| \| MYL9 \| \| MYLIP \| \| MYO18B \| \| MYO1D \| \| MYO5B \| \| MYOZ3 \| \| MYPN \| \| MEF2A \| \| MEMO1 \| \| MEOX1 \| \| MEST \| \| MFAP2 \| \| MFGE8 \| \| MFI2 \| \| MGA \| \| MGAT5 \| \| MGP \| \| MI \| \| MIB1 \| \| NBEA \| \| NBEAL1 \| \| NBPF10 \| \| NBR1 \| \| NCAM1 \| \| NCAPH \| \| NCDN \| \| NCKAP1 \| \| NCR00152 \| \| NDFIP1 \| \| NEB \| \| NEBL \| \| NEIL2 \| \| NEK7 \| \| NES \| \| NEU3 \| \| NFX1 \| \| NFYB \| \| NIN \| \| NIPAL3 \| \| NIPAL4 \| \| NIPSP1 \| \| NKIRAS2 \| \| NMT1 \| \| NOL10 \| \| NOS1 \| \| NOTCH2NL \| \| NOX4 \| \| NPC2 \| \| NPPB \| \| NPR3 \| \| NPTN \| \| NRIP3 \| \| NRK \| \| NT5C2 \| \| NT5DC1 \| \| NUDT16 \| \| NUS1 \| \| NUSAP1 \| \| NBEA \| \| NBEAL1 \| \| NBPF10 \| \| NBR1 \| \| NCAM1 \| \| NCAPH \| \| NCDN \| \| NCKAP1 \| \| NCR00152 \| \| NDFIP1 \| \| NEB \| \| NEBL \| \| NEIL2 \| \| NEK7 \| \| NES \| \| NEU3 \| \| NFX1 \| \| NFYB \| \| NIN \| \| NIPAL3 \| \| NIPAL4 \| \| NIPSP1 \| \| NKIRAS2 \| \| NMT1 \| \| NOL10 \| \| NOS1 \| \| NOTCH2NL \| \| NOX4 \| \| NPC2 \| \| NPPB \| \| NPR3 \| \| NPTN \| \| NRIP3 \| \| NRK \| \| NT5C2 \| \| NT5DC1 \| \| NUDT16 \| \| NUS1 \| \| NUSAP1 \| \| ODC1 \| \| OGDHL \| \| OPTN \| \| ORMDL3 \| \| OS9 \| \| OSBPL8 \| \| OSBPL9 \| \| OSGIN2 \| \| OSTM1 \| \| OXCT1 \| \| P1L4 \| \| P1L5 \| \| P2RX1 \| \| P2RX5 \| \| P4HA1 \| \| PABPC1 \| \| PACS1 \| \| PAFAH1B1 \| \| PAG1 \| \| PAK1 \| \| PAK3 \| \| PAM \| \| PAPPA2 \| \| PAQR4 \| \| PAQR8 \| \| PARN \| \| PBLD \| \| PCBP4 \| \| PCDH9 \| \| PCSK6 \| \| PCYT1A \| \| PDE1A \| \| PDE3B \| \| PDE4B \| \| PDE6B \| \| PDE8A \| \| PDE8B \| \| PDHX \| \| PDK3 \| \| PDLIM4 \| \| PDPN \| \| PDXDC1 \| \| PDXP \| \| PDZD8 \| \| PENK \| \| PF4 \| \| PFKP \| \| PGAP1 \| \| PGBD4 \| \| PGBD5 \| \| PGM5 \| \| PHF20L1 \| \| PHKB \| \| PI16 \| \| PI4K2A \| \| PICALM \| \| PIGN \| \| PIGT \| \| PIK3CA \| \| PIK3R3 \| \| PIKFYVE \| \| PJA1 \| \| PLAGL1 \| \| PLAUR \| \| PLCE1 \| \| PLEKHA2 \| \| PLEKHB2 \| \| PLK1 \| \| PNO1 \| \| PNRC1 \| \| POLH \| \| POLR1A \| \| POLR1E \| \| POMGNT1 \| \| PPCS \| \| PPM1A \| \| PPM1B \| \| PPM1E \| \| PPP1R14C \| \| PPP1R3C \| \| PPP2CA \| \| PPP2R5A \| \| PPP2R5C \| \| PPP4R1 \| \| PPT1 \| \| PPTC7 \| \| PRDM2 \| \| PREP \| \| PREPL \| \| PRICKLE1 \| \| PRKAR1A \| \| PRKCA \| \| PROS1 \| \| PRPH \| \| PSMD1 \| \| PSMD8 \| \| PSME4 \| \| PTGFRN \| \| PTP4A1 \| \| PTPDC1 \| \| PTPN1 \| \| PTPN11 \| \| PXDN \| \| QPCT \| \| PREP \| \| PREPL \| \| PRICKLE1 \| \| PRKAR1A \| \| PRKCA \| \| PROS1 \| \| PRPH \| \| PSMD1 \| \| PSMD8 \| \| PSME4 \| \| PTGFRN \| \| PTP4A1 \| \| PTPDC1 \| \| PTPN1 \| \| PTPN11 \| \| PXDN \| \| QPCT \| \| RAB23 \| \| RAB31 \| \| RAB3C \| \| RAB3GAP1 \| \| RAB9B \| \| RABEP1 \| \| RAC1 \| \| RAC3 \| \| RAD1 \| \| RAD21 \| \| RALBP1 \| \| RANBP9 \| \| RAP2A \| \| RARS2 \| \| RCAN1 \| \| RCN2 \| \| RCSD1 \| \| RDX \| \| RELL1 \| \| RERE \| \| RFESD \| \| RGAG1 \| \| RGL1 \| \| RGS3 \| \| RGS4 \| \| RHBDD1 \| \| RICTOR \| \| RIOK3 \| \| RLIM \| \| RNF11 \| \| RNF14 \| \| RNMT \| \| RNPEP \| \| ROR1 \| \| RP5-1022P6.6 \| \| RPL26L1 \| \| RPRD1A \| \| RPS6KA2 \| \| RPS6KL1 \| \| RRAGC \| \| RRAS2 \| \| RRM2 \| \| RTKN2 \| \| RTN3 \| \| SACS \| \| SAP30L \| \| SBNO1 \| \| SCAMP5 \| \| SCARB2 \| \| SCLY \| \| SCML2 \| \| SCN3B \| \| SCO1 \| \| SCRN1 \| \| SCUBE2 \| \| SEC24B \| \| SEC31A \| \| SEC61A1 \| \| SECISBP2L \| \| SEPHS1 \| \| SERBP1 \| \| SERINC2 \| \| SERINC3 \| \| SERPINE1 \| \| SERPINE2 \| \| SERPINH1 \| \| SESN2 \| \| SETD3 \| \| SETD7 \| \| SF3B2 \| \| SFT2D1 \| \| SGCB \| \| SGIP1 \| \| SGTA \| \| SH3D19 \| \| SH3GL2 \| \| SH3GLB1 \| \| SHB \| \| SIAH2 \| \| SIK2 \| \| SIRPA \| \| SKA1 \| \| SKIL \| \| SLAIN2 \| \| SLC16A1 \| \| SLC1A4 \| \| SLC22A15 \| \| SLC25A5 \| \| SLC31A1 \| \| SLC44A1 \| \| SLC47A1 \| \| SLC7A1 \| \| SLC9A1 \| \| SLK \| \| SMAD6 \| \| SMAD7 \| \| SMAD9 \| \| SMARCA5 \| \| SMCHD1 \| \| SMG1 \| \| SMOC2 \| \| SMYD1 \| \| SMYD2 \| \| SP23 \| \| SP29 \| \| SP47 \| \| SNCA \| \| SNIP1 \| \| SNX1 \| \| SNX27 \| \| SNX9 \| \| SOBP \| \| SOCS2 \| \| SOCS4 \| \| SOHLH2 \| \| SORBS1 \| \| SORBS2 \| \| SORT1 \| \| SPARC \| \| SPC24 \| \| SPG11 \| \| SPG21 \| \| SPINT2 \| \| SPIRE1 \| \| SPNS3 \| \| SPON1 \| \| SPRY4 \| \| SPTBN1 \| \| SQLE \| \| SRA1 \| \| SRL \| \| SRPK2 \| \| SRPX \| \| SRPX2 \| \| SS18 \| \| SSX2IP \| \| ST3GAL6 \| \| ST6GALC4 \| \| STAU1 \| \| STK17A \| \| STK38L \| \| STK39 \| \| STK40 \| \| STRN \| \| STS \| \| STX12 \| \| SUPT16H \| \| SUV39H2 \| \| SVIL \| \| SWAP70 \| \| SYAP1 \| \| SYNPO \| \| SYNPO2 \| \| SYT11 \| \| SYTL4 \| \| SYTL5 \| \| STK38L \| \| STK39 \| \| STK40 \| \| STRN \| \| STS \| \| STX12 \| \| SUPT16H \| \| SUV39H2 \| \| SVIL \| \| SWAP70 \| \| SYAP1 \| \| T1 \| \| TACC3 \| \| TAF9B \| \| TANC2 \| \| TAOK1 \| \| TAX1BP1 \| \| TBC1D1 \| \| TBC1D12 \| \| TBC1D22A \| \| TBC1D22B \| \| TBC1D30 \| \| TBCEL \| \| TEAD3 \| \| TEAD4 \| \| TEC \| \| TERF2IP \| \| TEX2 \| \| TGFB3 \| \| TGM2 \| \| THBS4 \| \| THOC6 \| \| TIMP1 \| \| TIPARP \| \| TK1 \| \| TLK1 \| \| TM6SF2 \| \| TMCC3 \| \| TMCO3 \| \| TMED3 \| \| TMEM5 \| \| TMEM51 \| \| TMEM66 \| \| TMEM71 \| \| TMEM72 \| \| TMX3 \| \| TNC \| \| TNFAIP1 \| \| TNFAIP6 \| \| TNFRSF11B \| \| TOMM34 \| \| TOP1 \| \| TOR1AIP2 \| \| TP53INP2 \| \| TPD52L1 \| \| TPM3 \| \| TPM4 \| \| TPX2 \| \| TRAF3 \| \| TRAF3IP1 \| \| TRAK1 \| \| TRAK2 \| \| TRIB1 \| \| TRIM24 \| \| TRIM37 \| \| TRIM41 \| \| TRIM55 \| \| TRIM59 \| \| TRIM69 \| \| TRIP11 \| \| TRIP13 \| \| TRMT12 \| \| TRUB1 \| \| TSC22D2 \| \| TSLP \| \| TSPAN1 \| \| TSPAN17 \| \| TSPAN9 \| \| TSPYL1 \| \| TSR1 \| \| TSSK4 \| \| TSTA3 \| \| TTBK2 \| \| TTC39C \| \| TTYH3 \| \| TUFT1 \| \| TUG1 \| \| TULP4 \| \| TWF1 \| \| TWSG1 \| \| TXNDC5 \| \| TYRP1 \| \| UACA \| \| UBAC2 \| \| UBE2G1 \| \| UBE2G2 \| \| UBE2H \| \| UBE2Q2 \| \| UBE2T \| \| UBFD1 \| \| UCHL1 \| \| UGGT1 \| \| UGP2 \| \| UHRF1 \| \| UHRF1BP1L \| \| ULBP2 \| \| UNC13C \| \| UNC45B \| \| USP11 \| \| USP12 \| \| USP15 \| \| USP22 \| \| USP39 \| \| USP46 \| \| USP53 \| \| USP6NL \| \| USP8 \| \| USP9X \| \| USP9Y \| \| UTP14C \| \| UTP3 \| \| VAPB \| \| VASH1 \| \| VCL \| \| VCPIP1 \| \| VDR \| \| VEGFC \| \| VEZT \| \| VLDLR \| \| VOPP1 \| \| VPS13A \| \| VPS35 \| \| VPS41 \| \| VPS8 \| \| VTA1 \| \| VTI1B \| \| WASL \| \| WBP11 \| \| WDFY3 \| \| WDR26 \| \| WDR67 \| \| WDR77 \| \| WEE1 \| \| WIPI1 \| \| WISP1 \| \| WSB1 \| \| WWTR1 \| \| XPR1 \| \| YEATS2 \| \| YIPF4 \| \| YME1L1 \| \| YPEL2 \| \| YRDC \| \| YWHAB \| \| YWHAQ \| \| ZBTB2 \| \| ZBTB47 \| \| ZBTB5 \| \| ZC3H13 \| \| ZC3H7A \| \| ZDBF2 \| \| ZDHHC23 \| \| ZDHHC3 \| \| ZEB1 \| \| ZFAND3 \| \| ZFP106 \| \| ZFP91 \| \| ZFR \| \| ZHX1 \| \| ZMAT3 \| \| ZMYND11 \| \| ZMYND17 \| \| ZNF20 \| \| ZNF28 \| \| ZNF318 \| \| ZNF323 \| \| ZNF326 \| \| ZNF415 \| \| ZNF461 \| \| ZNF462 \| \| ZNF563 \| \| ZNF654 \| \| ZNF808 \| \| ZNFX1 \| \| ZNRF2 \| \| ZSCAN29 \| \| 42989 (SEPTIN11)* \| \| 42980 (SEPTIN2)* \| \| 42797 (MARCHF3)* \| \| 42982 (SEPTIN4)* \| \| 42986 (SEPTIN8)* \| \|  \| \|  \| \|  \| \|  \| \|  \| \|  \| \|  \| \|  \| \|  \| \|  \| \|  \| \|  \| \|  \| \|  \| \|  \| \|  \| \|  \| \|  \| \|  \| \|  \| \|  \| \|  \| \|  \| \|  \| \|  \| \|  \| \|  \| \|  \| \|  \| \|  \| \|  \| \|  \| \|  \| \|  \| |
| **R/NR analyses, P <0.05** | | | | |
| **Upregulated** | | **Downregulated** | | |
| **Microarray** | **RNA-Seq** | **Microarray** | **RNA-Seq** | |
| A2BP1 | ABCA8 | A1BG | AASS | MAGED1 |
| ABCB10 | ABCC12 | ABCA8 | AATF | MAGED2 |
| ABHD3 | ACADVL | ACAA2 | ABAT | MAGI3 |
| ACADM | ADAM11 | ACAD10 | ABCD1 | MANSC1 |
| ACN9 | ADAMTS7 | ACADVL | ABHD12 | MAP1A |
| ACO2 | ADCYAP1R1 | ACAT1 | ABHD2 | MAP1B |
| ACSL1 | ADRB1 | ACSL1 | ABI2 | MAP1LC3A |
| ADAM11 | ALDH2 | ACSM5 | ACAD11 | MAP1LC3B |
| ADCY7 | ALS2CL | ACSS2 | ACBD3 | MAP2 |
| ADCYAP1R1 | AMD1 | ADAM11 | ACE2 | MAP2K1 |
| ADO | ANK1 | ADAM33 | ACOT9 | MAP3K10 |
| AFAP1L2 | ANKRD43 | ADAMTS15 | ACOX3 | MAP3K3 |
| AGBL3 | ANO4 | ADAMTS7 | ACTA1 | MAP4 |
| AIMP1 | AQP4 | ADCK4 | ACTN1 | MAP4K4 |
| ALAD2 | AQP7 | ADCY5 | ACTN2 | MAP7 |
| ALDH2 | ARHGAP10 | ADCYAP1R1 | ACTR10 | MAPK1 |
| ALG8 | ARRDC1 | ADD3 | ACTR2 | MAPK1IP1L |
| AMD1 | ART3 | ADH1B | ADAM9 | MAPKAPK5 |
| AMMECR1L | ART5 | ADRB1 | ADAMTS14 | MAPRE2 |
| AMN1 | ASB10 | AGBL2 | ADAMTS17 | MARK4 |
| ANKRD13C | ASB14 | AGTR1 | ADAMTS6 | MARS2 |
| ANKRD28 | ASPSCR1 | ALDH2 | ADC | MARVELD1 |
| ANKRD29 | ATP2A2 | ALDOC | ADH5 | MAVS |
| ANKRD6 | ATP5G2 | ANK1 | AEBP1 | MBOAT2 |
| ANO5 | ATP5I | ANKRD44 | AFF4 | MBTPS1 |
| AOX1 | AURKAIP1 | ANKZF1 | AKIRIN1 | MCAM |
| AP3S1 | BAIAP2L2 | AQP7 | ALDH3A2 | MCL1 |
| APLF | BANP | ARHGAP10 | ALKBH5 | MCM6 |
| APOOL | BCL11A | ARSI | ALS2 | MDFIC |
| APPL1 | BCL6 | ART3 | ALX3 | MED12L |
| AQP7 | BCL7A | ART5 | AMOTL1 | MED7 |
| ARGLU1 | BCL7C | ASB1 | ANKH | MEF2A |
| ARHGAP12 | BHMT | ASB10 | ANKIB1 | MEG3 |
| ARIH2 | BLVRB | ASB14 | ANKMY1 | MELK |
| ARL1 | BPHL | ASB16 | ANKRD13A | MEST |
| ARL5A | BSCL2 | ASB8 | ANKRD34C | MFAP2 |
| ARL5B | C10orf11 | ASPSCR1 | ANP32A | MFGE8 |
| ARMC1 | C11orf67 | ATP2A2 | ANXA11 | MFI2 |
| ARNT2 | C12orf33 | ATP5D | AP2B1 | MGP |
| ARPP19 | C14orf159 | ATP5G2 | AP3M2 | MI |
| ARRDC3 | C15orf59 | ATP5I | AP4E1 | MLA |
| ART3 | C15orf62 | ATP8B4 | APLP1 | MLF1IP |
| ASB10 | C1orf105 | AURKAIP1 | APLP2 | MLLT11 |
| ASPH | C1orf168 | AUTS2 | APOA1 | MMP15 |
| ATAD1 | C1orf170 | BAIAP2L2 | APOL2 | MOSC1 |
| ATP11A | C1orf95 | BCAR3 | APOL4 | MOXD1 |
| ATP11C | C20orf111 | BCKDHA | APOLD1 | MPPE1 |
| ATP5A1 | C20orf166 | BCL2L12 | APP | MPV17 |
| ATP5C1 | C2orf71 | BCL6 | APTX | MPV17L2 |
| ATP5G3 | C3orf43 | BCL7A | ARCN1 | MRFAP1 |
| ATP5I | C5orf54 | BCL7C | ARF1 | MTERFD3 |
| ATP5J | C6orf130 | BEND5 | ARF4 | MTHFD1L |
| ATP5J2 | C6orf225 | BLOC1S1 | ARHGAP1 | MTMR12 |
| ATP8B4 | C7orf10 | BLVRB | ARHGAP11A | MTMR3 |
| ATPBD4 | C7orf64 | BMP7 | ARHGEF12 | MTMR9 |
| ATPIF1 | C7orf70 | BPHL | ARHGEF7 | MTOR |
| B3GALNT1 | C8orf34 | BRSK2 | ARHGEF9 | MXRA5 |
| B4GALT6 | C8orf40 | BTN1A1 | ARIH1 | MXRA7 |
| BAT2L | CAC2D4 | BTN3A1 | ARL6IP5 | MYBL1 |
| BBS10 | CACYBP | C10orf11 | ARMCX2 | MYEF2 |
| BCAR3 | CAPS2 | C10orf116 | ARMCX3 | MYL1 |
| BCL11A | CCDC101 | C10orf128 | ARPC3 | MYL2 |
| BCL2L11 | CCDC28B | C10orf58 | ARSD | MYL9 |
| BCL6 | CCDC48 | C11orf67 | ARSE | MYOZ3 |
| BET1 | CCDC76 | C12orf33 | ASCC3 | NBEA |
| BLNK | CCR9 | C12orf57 | ASNS | NBEAL1 |
| BMI1 | CD200R1 | C13orf30 | ASPM | NCAM1 |
| BMPER | CD5L | C14orf159 | ATF6 | NCAPH |
| BMPR1A | CD99 | C15orf33 | ATF7IP2 | NCDN |
| BRMS1L | CDKN2AIPNL | C15orf38 | ATP13A3 | NDFIP2 |
| BTBD1 | CECR5 | C19orf70 | ATP1B1 | NEB |
| BTBD12 | CEL | C1orf105 | ATP2B1 | NEIL2 |
| BTF3L1 | CEND1 | C1orf151 | ATP2C1 | NEK7 |
| BTF3L4 | CFD | C1orf204 | ATP6V1B2 | NEU3 |
| BUB3 | CHCHD10 | C1orf95 | ATP6V1D | NGFR |
| BZW1 | CHDH | C1QL1 | ATP6V1E1 | NID1 |
| C10orf78 | CHRDL2 | C20orf7 | ATP6V1E2 | NIPAL3 |
| C11orf46 | CHRM2 | C21orf49 | ATP8B3 | NIT1 |
| C11orf71 | CHR2 | C21orf90 | ATP9A | NLGN1 |
| C11orf72 | CKM | C2orf71 | ATPAF1 | NLK |
| C11orf73 | CLYBL | C3 | ATRNL1 | NMT2 |
| C11orf87 | CNNM1 | C3orf37 | AZIN1 | NOM1 |
| C12orf39 | COL28A1 | C3orf43 | B3GNT5 | NOTCH2NL |
| C13orf18 | COL4A6 | C3orf45 | B4GALT4 | NOX4 |
| C13orf30 | COL9A3 | C3orf55 | B4GALT5 | NPC2 |
| C13orf37 | COLEC12 | C5orf54 | BCL2L2 | NPPB |
| C14orf4 | COPS5 | C6orf130 | BECN1 | NPR3 |
| C15orf61 | CORIN | C6orf57 | BEX1 | NRIP3 |
| C16orf52 | COX6A2 | C7orf10 | BEX4 | NRK |
| C16orf54 | COX7A1 | C7orf63 | BGN | NRP1 |
| C17orf48 | CPA3 | C7orf70 | BICD1 | NT5C2 |
| C17orf58 | CXCR6 | C8orf34 | BICD2 | NUAK1 |
| C17orf77 | CXXC1 | C8orf40 | BIRC6 | NUCB2 |
| C18orf19 | CYC1 | CA1 | BLMH | NUDT16 |
| C18orf54 | DEGS2 | CA14 | BMPR2 | NUSAP1 |
| C1D | DHFRL1 | CA4 | BMS1 | ODC1 |
| C1orf104 | DHRS12 | CAB39L | BRAF | OGDHL |
| C1orf105 | DHRS7C | CAC2D4 | BTBD1 | OLFML2A |
| C1orf186 | DLK1 | CAPS2 | BTBD3 | OPTN |
| C20orf177 | DH11 | CATSPERB | BTBD7 | ORMDL3 |
| C20orf24 | DOCK10 | CCDC101 | BTG1 | OS9 |
| C21orf34 | EEPD1 | CCDC17 | BUB1B | OSBPL2 |
| C21orf87 | EFCAB4B | CCDC28B | BUD31 | OSBPL9 |
| C2orf40 | EFHC2 | CCDC39 | BVES | OSTM1 |
| C3orf43 | EGFLAM | CCDC88C | C10orf46 | OXCT1 |
| C3orf48 | EME2 | CCR9 | C10orf93 | P1L1 |
| C4orf21 | EPB42 | CCRN4L | C11orf24 | P1L4 |
| C4orf3 | EPS8L3 | CD52 | C11orf80 | P4HA1 |
| C4orf32 | ETFB | CD5L | C12orf23 | PABPC1 |
| C4orf43 | EXOSC10 | CD7 | C12orf32 | PACRG |
| C7orf70 | FADD | CD8A | C12orf43 | PACS1 |
| C8orf37 | FAM124A | CD96 | C12orf48 | PACSIN1 |
| C8orf4 | FAM132B | CDH13 | C12orf49 | PAIP2 |
| C8orf45 | FAM159A | CDH26 | C12orf66 | PAK4 |
| C8orf59 | FAM179A | CDYL | C14orf135 | PALM2-AKAP2 |
| C9orf29 | FAM19A2 | CECR5 | C15orf23 | PAM |
| C9orf95 | FAM26F | CEL | C15orf42 | PANX1 |
| CA14 | FAM78B | CENPV | C16orf91 | PAPD5 |
| CAB39L | FAM81B | CEP192 | C17orf63 | PAPPA2 |
| CACNB2 | FAM84A | CEP68 | C17orf85 | PARD6A |
| CACYBP | FAU | CFD | C19orf63 | PARVA |
| CADM2 | FBXO45 | CFLAR | C1orf190 | PBLD |
| CAPN2 | FGFBP2 | CHADL | C1orf192 | PCDH20 |
| CAPZA2 | FITM1 | CHCHD10 | C1orf198 | PCDH9 |
| CAV1 | FNBP4 | CHDH | C1orf21 | PCSK5 |
| CBLL1 | FNDC5 | CHI3L1 | C1orf96 | PCSK6 |
| CCDC111 | FRS2 | CHPT1 | C20orf194 | PCYT1A |
| CCDC126 | ABCA8 | CHRM2 | C21orf7 | PDCD10 |
| CCDC138 | ABCC12 | CKM | C2orf65 | PDE1A |
| CCNT2 | ACADVL | CNBP | C2orf67 | PDE6B |
| CCPG1 | ADAM11 | CNTFR | C2orf70 | PDE8B |
| CD164 | ADAMTS7 | COL28A1 | C2orf81 | PDLIM1 |
| CD302 | ADCYAP1R1 | COPS5 | C4orf14 | PDLIM4 |
| CD5L | ADRB1 | COPS6 | C4orf29 | PDPN |
| CD8A | ALDH2 | COQ10A | C4orf48 | PDZD7 |
| CDKN1B | ALS2CL | COQ4 | C4orf49 | PEA15 |
| CECR6 | AMD1 | COQ9 | C5orf30 | PENK |
| CEL | ANK1 | CORIN | C5orf46 | PGAM1 |
| CELA1 | ANKRD43 | COX19 | C6orf138 | PGAP1 |
| CENPQ | ANO4 | COX4I1 | C6orf35 | PGBD5 |
| CEP97 | AQP4 | COX5A | C6orf72 | PHACTR2 |
| CHD2 | AQP7 | COX5B | C7orf41 | PHC1 |
| CHDH | ARHGAP10 | COX6A2 | C7orf53 | PHTF2 |
| CHORDC1 | ARRDC1 | COX6B1 | C7orf60 | PHYHIP |
| CHPT1 | ART3 | COX7A1 | C9orf125 | PI16 |
| CIDEB | ART5 | COX8A | C9orf16 | PIAS1 |
| CKM | ASB10 | CPA3 | C9orf30 | PIAS2 |
| CLCN4 | ASB14 | CPO | CALD1 | PICALM |
| CLDN12 | ASPSCR1 | CPT1B | CALML6 | PIGT |
| CLDND1 | ATP2A2 | CREB3L4 | CALU | PIK3R2 |
| CLGN | ATP5G2 | CRYGS | CAMK1G | PIK3R3 |
| CLK4 | ATP5I | CTAGE5 | CAMK2A | PIKFYVE |
| CLPX | AURKAIP1 | CTNNBIP1 | CAMK2D | PJA1 |
| CMC1 | BAIAP2L2 | CTNNBL1 | CAMK2G | PLA2G4C |
| CMPK1 | BANP | CTSG | CAPRIN1 | PLAGL1 |
| CMPK2 | BCL11A | CTSW | CAPRIN2 | PLCD4 |
| CN5H6.4 | BCL6 | CXCR6 | CASQ1 | PLCE1 |
| CNBP | BCL7A | CXXC1 | CASQ2 | PLEKHA2 |
| CNOT6 | BCL7C | CYC1 | CBFA2T2 | PLEKHA3 |
| COG5 | BHMT | CYP4Z1 | CBL | PLEKHA4 |
| COL15A1 | BLVRB | DEDD | CC2 | PLEKHB2 |
| COL28A1 | BPHL | DENND2D | CC2 | PLIN3 |
| COL4A3 | BSCL2 | DHFRL1 | CCDC102B | PNMA1 |
| COLEC12 | C10orf11 | DHRS12 | CCDC111 | PNRC1 |
| COMMD3 | C11orf67 | DHRS7C | CCDC113 | POLH |
| COPB1 | C12orf33 | DIO3OS | CCDC137 | POLR1A |
| COQ3 | C14orf159 | DIXDC1 | CCDC14 | POLR1C |
| CORIN | C15orf59 | DJA3 | CCDC157 | POLR1E |
| COX11 | C15orf62 | DOCK10 | CCDC50 | POMGNT1 |
| COX16 | C1orf105 | DPF2 | CCIN | PORCN |
| COX17 | C1orf168 | DSCAML1 | CCND2 | POSTN |
| COX5A | C1orf170 | DTX2 | CCNG2 | PPAP2A |
| COX7A2L | C1orf95 | DYRK2 | CCNJL | PPCS |
| COX7B | C20orf111 | ECD | CD151 | PPFIA1 |
| COX7C | C20orf166 | ECH1 | CD274 | PPHLN1 |
| CPA3 | C2orf71 | ECHDC3 | CD59 | PPIC |
| CPNE4 | C3orf43 | EDA | CDC25A | PPM1E |
| CREBBP | C5orf54 | EEPD1 | CDC27 | PPP1R14C |
| CRIPT | C6orf130 | EFHC2 | CDH17 | PPP1R3C |
| CRLS1 | C6orf225 | EGFLAM | CDH2 | PPP2R5A |
| CRY1 | C7orf10 | EID2B | CDK2AP1 | PPP2R5D |
| CS | C7orf64 | ENTPD3 | CDK6 | PPP3R2 |
| CTAGE5 | C7orf70 | ENTPD6 | CDK8 | PPP4R1 |
| CTR9 | C8orf34 | EPB41L2 | CDKN2B | PPTC7 |
| CUL3 | C8orf40 | EPC1 | CDKN3 | PRAM1 |
| CX3CR1 | CAC2D4 | EPDR1 | CDYL2 | PRCP |
| CXorf24 | CACYBP | ERC1 | CENPA | PRDM2 |
| CYP4Z2P | CAPS2 | ESPN | CENPK | PREPL |
| CYTIP | CCDC101 | ESRP2 | CENPN | PRG4 |
| DCK | CCDC28B | ETFB | CHD3 | PRICKLE1 |
| DCP2 | CCDC48 | EXOSC3 | CHD5 | PRICKLE2 |
| DDX20 | CCDC76 | EXOSC5 | CHD8 | PRKCSH |
| DDX5 | CCR9 | EXPH5 | CHMP4B | PRKY |
| DEK | CD200R1 | FADD | CHMP4C | PROS1 |
| DGAT2 | CD5L | FAM123B | CHMP5 | PRPH |
| DH11 | CD99 | FAM124A | CHPF2 | PRPSAP2 |
| DJB9 | CDKN2AIPNL | FAM175A | CHRM5 | PRSS23 |
| DLAT | CECR5 | FAM179A | CHST15 | PSAP |
| DLK1 | CEL | FAM46A | CILP | PTGFRN |
| DOCK8 | CEND1 | FAM46C | CKAP4 | PTGR1 |
| DPY19L2 | CFD | FAM65C | CLCN3 | PTPN1 |
| DPYSL2 | CHCHD10 | FAM78A | CLIC4 | PTPN11 |
| DSCR8 | CHDH | FAM81A | CLOCK | PTPRH |
| DSP | CHRDL2 | FAM84A | CLPTM1 | QPCT |
| DYRK2 | CHRM2 | FAU | CLTCL1 | QSER1 |
| EBAG9 | CHR2 | FBXO46 | CMAS | QSOX1 |
| EDEM3 | CKM | FDFT1 | CNKSR3 | RAB11A |
| EFHA1 | CLYBL | FEZ1 | CNN1 | RAB13 |
| EFHA2 | CNNM1 | FGF12 | COBLL1 | RAB15 |
| EFNB3 | COL28A1 | FGFBP2 | COL16A1 | RAB18 |
| EFR3A | COL4A6 | FIGN | COL18A1 | RAB20 |
| EGFR | COL9A3 | FITM1 | COL1A1 | RAB31 |
| EHMT1 | COLEC12 | FLCN | COL1A2 | RAB3C |
| EID2B | COPS5 | FLRT1 | COL23A1 | RAB6B |
| EIF1AX | CORIN | FLT3LG | COL3A1 | RAB9B |
| EIF2B1 | COX6A2 | FMO2 | COL4A1 | RAC3 |
| EIF3E | COX7A1 | FNBP4 | COL5A1 | RAD1 |
| EIF3M | CPA3 | FNDC5 | COL5A2 | RAD21 |
| EIF4E | CXCR6 | FOXN3 | COL8A1 | RAI2 |
| EIF4E3 | CXXC1 | FUNDC2 | COMMD1 | RALBP1 |
| ENOSF1 | CYC1 | FXYD2 | COMMD8 | RANBP9 |
| ENOX1 | DEGS2 | G0S2 | COPA | RAP2A |
| ENPP4 | DHFRL1 | GABBR2 | COPB2 | RAP2B |
| EPC1 | DHRS12 | GALNTL1 | COX15 | RARS2 |
| EPC2 | DHRS7C | GBX1 | CPVL | RASL11B |
| EPS15 | DLK1 | GCOM1 | CPZ | RASSF7 |
| ESRRG | DH11 | GIMAP1 | CREG1 | RBBP7 |
| ESYT2 | DOCK10 | GIMAP5 | CREG2 | RBMS3 |
| EVI2A | EEPD1 | GIMAP7 | CRELD1 | RCAN1 |
| EWSR1 | EFCAB4B | GLTSCR2 | CRKL | RCSD1 |
| EXOC5 | EFHC2 | GLYCTK | CRLF1 | REEP3 |
| EXPH5 | EGFLAM | GPD1 | CRTAP | RELB |
| FAM104B | EME2 | GPIHBP1 | CRYBG3 | RELL1 |
| FAM124A | EPB42 | GPR37L1 | CSDA | RGL1 |
| FAM127B | EPS8L3 | GPSM1 | CSDE1 | RGP1 |
| FAM162A | ETFB | GREB1 | CSGALCT2 | RGS4 |
| FAM175A | EXOSC10 | GRM2 | CSNK2A2 | RIOK3 |
| FAM179A | FADD | GSTM2 | CSPG5 | RNF11 |
| FAM27E3 | FAM124A | GSTM5 | CTGF | RNF14 |
| FAM46C | FAM132B | GSTP1 | CTSA | RNF170 |
| FAM63B | FAM159A | GTF3C5 | CTSB | RNF214 |
| FAM70A | FAM179A | GZMK | CTTN | RNMT |
| FAM8A1 | FAM19A2 | GZMM | CUL4B | ROR1 |
| FAM96A | FAM26F | HACL1 | CUX1 | RPL26L1 |
| FAM98B | FAM78B | HADHA | CXADR | RPRD1A |
| FBXL4 | FAM81B | HADHB | CXCR7 | RPS6KA2 |
| FDX1 | FAM84A | HEATR2 | CXorf40A | RPS6KL1 |
| FGD3 | FAU | HERPUD1 | CYB5R1 | RRAS |
| FGF12 | FBXO45 | HEY2 | CYB5R3 | RS |
| FGF7 | FGFBP2 | HIBADH | CYLD | RSF1 |
| FGFBP2 | FITM1 | HIPK3 | CYP11A1 | RSPH3 |
| FGFBP3 | FNBP4 | HIPK4 | CYP19A1 | RTN3 |
| FH | FNDC5 | HIRIP3 | CYTH2 | S100A16 |
| FHIT | FRS2 | HIST1H1C | DACT3 | S1PR1 |
| FKBP7 | G0S2 | HIST1H2BF | DAZAP2 | SACS |
| FKBP9 | GALNTL1 | HIST3H2A | DCBLD2 | SCAMP5 |
| FKTN | GALNTL6 | HLF | DCLK3 | SCARB2 |
| FLJ31713 | GBA2 | HMGCS2 | DCTN1 | SCLY |
| FLJ36644 | GCOM1 | HOMER2 | DCUN1D3 | SCMH1 |
| FLJ42289 | GNPTAB | HSD17B3 | DDA1 | SCO1 |
| FMR1 | GPR133 | HSDL2 | DDAH1 | SCRN1 |
| FNDC5 | GPR160 | HSPB2 | DDB2 | SDSL |
| FOXN2 | GPT | HSPB3 | DDO | SEC24B |
| FOXN3 | GREB1 | HTRA2 | DDX24 | SEC31A |
| FPGT | GRIN2A | IDH2 | DDX3X | SECISBP2L |
| FRAS1 | GRM2 | IDH3B | DDX46 | SEMA5A |
| FTHP1 | GTF3C5 | IFNK | DDX5 | SEPHS1 |
| FTO | GZMK | IFRD2 | DDX60L | SERBP1 |
| FTSJD1 | H3F3B | IGDCC4 | DENND5A | SERINC2 |
| FUBP1 | HADHB | IGHG1 | DENND5B | SERINC3 |
| FUNDC2 | HEXIM2 | IGKC | DES | SERPINE1 |
| FZD3 | HEY2 | IKZF3 | DH1 | SERPINE2 |
| G13 | HIBADH | IL18RAP | DHCR24 | SESN2 |
| GALC | HIPK4 | ILKAP | DIS3 | SESN3 |
| GALNTL1 | HIST3H2A | ILVBL | DJA4 | SETD3 |
| GAR1 | HMGCS2 | IMPA2 | DJC13 | SETD7 |
| GAS2 | HOMER2 | INTS9 | DLG1 | SF3B2 |
| GBAS | HSD17B3 | ISLR2 | DLK2 | SGIP1 |
| GHR | HSPB3 | ITGA8 | DMXL2 | SGK1 |
| GIMAP2 | IDH3B | ITGB8 | DNMT1 | SGTA |
| GIMAP8 | IFNK | ITIH4 | DOC2B | SH2D4A |
| GINS3 | IFRD2 | ITPRIPL1 | DOK4 | SH3D19 |
| GIPC2 | IGKV4-1 | JARID2 | DPP4 | SH3GL2 |
| GLRX5 | IKZF3 | JHDM1D | DSE2 | SH3GLB1 |
| GMEB2 | IL24 | KANK1 | DSEL | SH3TC1 |
| GNG5 | IL6R | KC2 | DSYN1 | SHB |
| GOLPH3L | ILKAP | KC6 | DT | SHC2 |
| GPBP1 | IMPA2 | KCND3 | DTX4 | SHROOM3 |
| GPC5 | ITCH | KCNH8 | DUSP15 | SIAH2 |
| GPN3 | ITGB8 | KCNIP2 | DUSP27 | SIK2 |
| GPR146 | JARID2 | KCNIP4 | DUSP5 | SIRPA |
| GPR22 | KAT2B | KCNJ11 | DYNC1LI1 | SKA1 |
| GPRC5B | KCNC1 | KCNMA1 | DYNC1LI2 | SLAIN2 |
| GQ | KCNIP4 | KIAA0408 | DYNLT1 | SLC16A1 |
| GRAMD1C | KCNJ11 | KIF22 | DYNLT3 | SLC1A4 |
| GRIN2A | KCNJ2 | KLF9 | DYSF | SLC1A7 |
| GRSF1 | KCNMB2 | KLHDC3 | E2F1 | SLC25A5 |
| GS | KHK | KLHL21 | E2F3 | SLC35A2 |
| GSDMC | KIAA0408 | KLHL22 | ECM2 | SLC44A1 |
| GZMK | KIAA1328 | KLHL24 | ECT2 | SLC47A1 |
| H1F0 | KLHDC3 | KLHL7 | EDA2R | SLC9A1 |
| HADH | KLHL24 | KLKB1 | EEF1A1 | SLCO1B3 |
| HADHB | KLKB1 | KLRF1 | EFR3A | SMAD1 |
| HAS2 | LAMC2 | KLRK1 | EH | SMAD2 |
| HAUS3 | LARP4B | LAMC2 | EHBP1L1 | SMAD6 |
| hCG_1647286 | LPAR3 | LCNL1 | EID2 | SMAD9 |
| HCG4 | LPAR5 | LDB2 | EIF2C4 | SMOC2 |
| HDGF | LPXN | LGI4 | EIF2S3 | SMYD1 |
| HEATR5A | LRIT1 | LGR6 | EIF4E3 | SMYD2 |
| HELQ | LRRC39 | LNX1 | EIF4G3 | SNCA |
| HEY2 | MAL | LPAR3 | ELAVL3 | SNIP1 |
| HIAT1 | MAML2 | LPAR5 | ELF1 | SNX1 |
| HIBCH | MAP3K14 | LPCAT4 | ELOVL5 | SNX27 |
| HIF1A | MAP3K5 | LRRC16B | EM | SOCS2 |
| HINT1 | MCF2 | LRRC39 | EML2 | SORBS2 |
| HINT3 | MCOLN1 | LRRN3 | EMP1 | SORT1 |
| HISPPD1 | MED13L | LSS | ENC1 | SP23 |
| HIST1H1D | MEPCE | LYRM1 | ENO3 | SP29 |
| HIST1H2BD | MFSD11 | MACROD2 | ENOX2 | SPAG4 |
| HIST1H2BF | MID1IP1 | MAL | ENPP1 | SPARC |
| HIST3H2A | MIR17HG | MAML2 | ENTPD4 | SPG21 |
| HLF | MLPH | MAP3K5 | ERCC4 | SPIN4 |
| HLTF | MRO | MAPKAPK3 | ERLEC1 | SPINT2 |
| HMGB1 | MRPL21 | MARVELD3 | ERMP1 | SPIRE1 |
| HMGCS2 | MRPL24 | MAX | ETF1 | SPNS3 |
| HNRNPA0 | MRPL38 | MCOLN1 | ETV1 | SPR |
| HNRNPA1 | MRPL43 | MED13L | EXOC6B | SPTAN1 |
| HNRNPA2B1 | MRPS23 | MESP1 | EXT1 | SPTBN1 |
| HNRNPA3 | MRPS24 | METTL7B | EXTL3 | SQLE |
| HNRNPK | MRPS9 | MFNG | EZR | SRA1 |
| HNRNPR | MT1X | MFSD11 | F2R | SRL |
| HNRPDL | MTHFS | MLLT10 | F3 | SRPX |
| HPCAL4 | MTSS1 | MLLT6 | FADS1 | SRPX2 |
| HPGDS | MYH6 | MLPH | FAM104A | SSPN |
| HSDL2 | MYL3 | MMP11 | FAM108C1 | SSX2IP |
| HSF2 | MYLK4 | MPP1 | FAM110B | ST3GAL3 |
| HSPB3 | N6AMT1 | MPP3 | FAM114A1 | ST3GAL4 |
| IDO2 | RS2 | MRO | FAM114A2 | ST3GAL6 |
| IER3IP1 | NCAM2 | MRPL2 | FAM115A | ST6GALC4 |
| IGFBP7 | NCR00201 | MRPL21 | FAM120A | STAU1 |
| IGSF11 | NDUFA13 | MRPL23 | FAM122B | STK17A |
| IL6R | NDUFB10 | MRPL24 | FAM155A | STK17B |
| IPO7 | NDUFB7 | MRPL38 | FAM169A | STK38L |
| IRAK2 | NDUFB9 | MRPL43 | FAM171A1 | STK39 |
| IRF2 | NDUFS7 | MRPL48 | FAM173B | STK40 |
| IRF2BP2 | NETO1 | MRPL51 | FAM176A | STMN1 |
| ITGB6 | NFE2L3 | MRPS15 | FAM177A1 | STOM |
| ITK | NHEJ1 | MRPS24 | FAM18B2 | STRN |
| JKAMP | NKAPL | MRPS25 | FAM48A | STS |
| KAT2B | NKRF | MS4A1 | FAM50A | STX12 |
| KBTBD7 | NPC1 | MT1X | FAM83D | STX1B |
| KC6 | NUP35 | MT3 | FANCI | STYXL1 |
| KCNJ2 | OPLAH | MTIF3 | FAP | SUMO3 |
| KCTD12 | OSCP1 | MTSS1 | FARP1 | SUSD2 |
| KCTD9 | PAN2 | MYCL1 | FATE1 | SV2C |
| KDM5C | PARD6G | MYL3 | FBXO27 | SVEP1 |
| KDSR | PCDHB6 | MYLK4 | FBXO30 | SVIL |
| KGFLP2 | PCDHGA10 | MYOF | FBXW11 | SWAP70 |
| KIAA0528 | PDE3A | N6AMT1 | FEZ2 | SYAP1 |
| KIAA0895 | PDE6C | RS2 | FGD4 | SYNJ2BP |
| KIAA0907 | PDE7B | NCAM2 | FGD6 | SYNPO |
| KIAA1012 | PDIK1L | NCKAP5 | FGF13 | SYNPO2 |
| KIAA1143 | PEBP4 | NCR00161 | FGFR1 | SYNRG |
| KIAA1267 | PEX11A | NDUFA13 | FGFRL1 | SYT9 |
| KIAA1328 | PGAM2 | NDUFB10 | FHAD1 | SYTL2 |
| KIAA1466 | PHACTR3 | NDUFB11 | FHL1 | SYTL4 |
| KIAA1712 | PHKG1 | NDUFB3 | FILIP1L | SYTL5 |
| KL | PIM3 | NDUFB7 | FMN1 | TACR2 |
| KLF10 | PKP2 | NDUFB9 | FN1 | TANC2 |
| KLHL15 | PLA2G4F | NDUFC2 | FOXC2 | TAOK1 |
| KLRK1 | PLAG1 | NDUFS2 | FOXM1 | TBC1D1 |
| KRAS | PLCL2 | NDUFS3 | FRRS1 | TBC1D22A |
| KRIT1 | PLD6 | NDUFS6 | FSD1L | TBC1D22B |
| LACTB2 | PLEKHG4B | NDUFS7 | FTH1 | TBCD |
| LAPTM4B | PLXNB1 | NDUFS8 | FZD1 | TBCEL |
| LARP4B | PNMA3 | NFXL1 | FZD3 | TBL1XR1 |
| LCORL | POLM | NPC1 | FZD8 | TBP |
| LDB3 | POLR2I | NPHP3 | G14 | TCEAL3 |
| LEKR1 | PPAPDC3 | NR3C2 | GABARAPL2 | TCF25 |
| LEMD3 | PPFIBP2 | NRXN1 | GADD45A | TDP1 |
| LIFR | PPIL6 | NUDT13 | GALNT10 | TEAD3 |
| LIN7C | PPP1R1A | NUDT4 | GALNT12 | TECR |
| LINS1 | PPP2R3C | NUP35 | GDAP1 | TERF2IP |
| LOC100128822 | PRELID2 | NXPH3 | GDF11 | TEX2 |
| LOC100129633 | PRKCQ | OSBPL1A | GDI2 | TFCP2L1 |
| LOC100130219 | PRPF38A | OSBPL5 | GFM2 | TFDP2 |
| LOC100131512 | PTPRCAP | P2RY1 | GGA2 | TFG |
| LOC100132418 | PUS7L | PAN2 | GI1 | TGFB3 |
| LOC100270804 | PWWP2A | PANK4 | GJA3 | TGM2 |
| LOC144438 | PXMP2 | PCDHGA10 | GLG1 | TGOLN2 |
| LOC146429 | PZP | PCDHGA11 | GLRB | THBS4 |
| LOC148189 | NPC1 | PCDHGB6 | GLRX | THOC6 |
| LOC153346 | NUP35 | PCDHGB7 | GLS | TIFAB |
| LOC157381 | OPLAH | PCDHGB8P | GLT1D1 | TIMP1 |
| LOC171220 | OSCP1 | PCMTD2 | GLT25D1 | TJP1 |
| LOC221710 | PAN2 | PDE2A | GLT8D2 | TLE4 |
| LOC255512 | PARD6G | PDE3A | GLTP | TM2D2 |
| LOC283588 | PCDHB6 | PDE7B | GMPR2 | TM6SF2 |
| LOC284408 | PCDHGA10 | PDIK1L | GNG12 | TMCC3 |
| LOC285972 | PDE3A | PEBP4 | GNS | TMED10 |
| LOC286052 | PDE6C | PEX11A | GOLGA2 | TMED3 |
| LOC286178 | PDE7B | PFKFB3 | GOLIM4 | TMEFF1 |
| LOC339751 | PDIK1L | PGAM2 | GOPC | TMEM107 |
| LOC340544 | PEBP4 | PHACTR3 | GPC1 | TMEM30A |
| LOC388387 | PEX11A | PHF15 | GPR137B | TMEM51 |
| LOC389834 | PGAM2 | PHKG1 | GPR137C | TMEM56 |
| LOC401397 | PHACTR3 | PHPT1 | GPR27 | TMEM71 |
| LOC439911 | PHKG1 | PID1 | GPR63 | TMEM72 |
| LOC440552 | PIM3 | PIGL | GRM1 | TMEM9B |
| LOC643008 | PKP2 | PIM3 | GRN | TNNT1 |
| LOC645676 | PLA2G4F | PKD1L1 | GSDMB | TNPO3 |
| LOC646903 | PLAG1 | PKP2 | GSG2 | TOP2A |
| LOC647979 | PLCL2 | PLA2G4F | GSPT1 | TOR1AIP2 |
| LOC653739 | PLD6 | PLAG1 | GUF1 | TP53INP2 |
| LOC728411 | PLEKHG4B | PLCL2 | GYG1 | TPD52L1 |
| LOC728804 | PLXNB1 | PLD1 | GYS1 | TPM2 |
| LOC729350 | PNMA3 | PLD6 | GZF1 | TPM3 |
| LPAR3 | POLM | PLEKHF1 | H19 | TPX2 |
| LPAR4 | POLR2I | PLEKHG4B | H2AFV | TRAF3 |
| LPCAT2 | PPAPDC3 | PLXNB1 | H2AFX | TRAF3IP1 |
| LRP11 | PPFIBP2 | POLE | HABP4 | TRAFD1 |
| LRP4 | PPIL6 | POLM | HAPLN1 | TRAK2 |
| LRPPRC | PPP1R1A | POLR2I | HCCS | TRIM24 |
| LRRC39 | PPP2R3C | POLR2L | HDAC2 | TRIM37 |
| LRRC57 | PRELID2 | PPAPDC3 | HECA | TRIM41 |
| LRRC66 | PRKCQ | PPARGC1B | HELB | TRIM72 |
| LRRK2 | PRPF38A | PPM1L | HEPH | TRIP13 |
| LSM5 | PTPRCAP | PPP1R1A | HERC2 | TRO |
| LSM8 | PUS7L | PQBP1 | HERC4 | TRUB1 |
| LUZPP1 | PWWP2A | PRCD | HERC5 | TSHZ1 |
| LYPLA1 | PXMP2 | PRDX1 | HEXA | TSPAN1 |
| LYPLAL1 | PZP | PRDX5 | HEXB | TSPAN3 |
| LYRM1 | RAB11FIP4 | PRELID2 | HIF1AN | TSPAN5 |
| LYRM5 | RAB37 | PRF1 | HIP1R | TSPAN9 |
| MAF | RAF1 | PRKCE | HIPK1 | TSPYL1 |
| MAGI2 | RCOR2 | PRODH | HIPK2 | TSTA3 |
| MAMDC2 | RET | PRSS36 | HIST1H2AL | TTBK2 |
| MAP3K8 | REV1 | PSMB10 | HIST1H3B | TTC8 |
| MAPK6 | RGMA | PSMB3 | HK1 | TUBB2B |
| MBD4 | RNF113A | PSMB6 | HLX | TUFT1 |
| MBIP | RNF165 | PTCH1 | HMGB3 | TUG1 |
| MBLAC2 | RNF207 | PTGDS | HMGN4 | TULP4 |
| MCF2 | RORC | PTPRCAP | HMMR | TWSG1 |
| MCM3APAS | RPAP2 | PWWP2A | HOXB2 | TXNDC5 |
| MCM9 | RPL10 | PXMP2 | HRH4 | UBA6 |
| MDM1 | RPL18 | PYHIN1 | HSP90AB1 | UBAC2 |
| MED21 | RPL24 | PZP | HSPA13 | UBB |
| MED28 | RPL3L | RAB40B | HSPA2 | UBE2H |
| MED4 | RPS21 | RAF1 | HSPA4L | UBE2Q2 |
| MEIS3P1 | RPS27A | RAGE | HTRA1 | UBE2S |
| METAP1 | RPS28 | RBM17 | HYLS1 | UBFD1 |
| METTL4 | RPS3 | RBM28 | ICMT | UBP1 |
| MGC13053 | RPUSD2 | RCOR2 | IGF2R | UBQLN1 |
| MGC70870 | RPUSD3 | RDH14 | IGFBP2 | UBR1 |
| MGST2 | RSAD1 | RET | IL17D | UCHL1 |
| MLPH | RSPH9 | REV1 | IL1R1 | UHRF1 |
| MOBKL1A | SAT2 | RG9MTD3 | IMPA1 | UHRF1BP1L |
| MPT | SCG3 | RGMA | IMPAD1 | UNC13C |
| MRO | SCN1A | RHAG | INPP4B | USP11 |
| MRPL13 | SCN7A | RIMS1 | INPP5A | USP13 |
| MRPL19 | SDHAF1 | RMST | INTS3 | USP22 |
| MRPL22 | SDK1 | RNF148 | INTS8 | USP46 |
| MRPL32 | SEC14L5 | RNF165 | IPO11 | USP53 |
| MRPL35 | SELENBP1 | RNF187 | IQGAP3 | USP6NL |
| MRPL43 | SEPP1 | RNF207 | IQSEC2 | USP9X |
| MRPL44 | SGSM1 | RNLS | IQUB | UTRN |
| MRPL48 | SH3RF2 | ROR2 | IREB2 | VANGL1 |
| MRPL51 | SHISA3 | RORC | IRGQ | VAPB |
| MRPL9 | SLC10A1 | RPL10 | IRX1 | VCAM1 |
| MRPS24 | SLC12A8 | RPL14 | ITFG3 | VDR |
| MRPS25 | SLC13A4 | RPL18 | ITGAV | VEGFC |
| MRPS35 | SLC15A3 | RPL19 | ITGB1 | VGLL3 |
| MTERFD1 | SLC19A2 | RPL22 | ITPR1 | VKORC1L1 |
| MTF2 | SLC25A26 | RPL24 | ITSN1 | VLDLR |
| MTPAP | SLC26A9 | RPL27A | JAM3 | VPS13A |
| MTRR | SLC27A6 | RPL34 | JAZF1 | VWCE |
| MTSS1 | SLC43A1 | RPL35 | KANK2 | WASF3 |
| MXI1 | SPC5 | RPL36 | KBTBD10 | WASL |
| MYL3 | SNRNP25 | RPL37A | KCB1 | WBP11 |
| MYLK4 | SNRNP70 | RPL38 | KCMF1 | WBP2 |
| MYST4 | SOSTDC1 | RPL5 | KCNC4 | WDR26 |
| NCKAP1 | SOX10 | RPLP0 | KCNJ4 | WDR44 |
| NCOA3 | SPATA6 | RPS10 | KCNJ5 | WDR67 |
| NCOA4 | SPATC1 | RPS11 | KCNK6 | WDR77 |
| NCR00201 | STARD3 | RPS14 | KCNMB1 | WEE1 |
| ND2 | SYCP3 | RPS21 | KCNQ1 | WHSC1 |
| NDUFA12 | SYT3 | RPS23 | KCTD17 | WIPI1 |
| NDUFA4 | TAGAP | RPS24 | KCTD2 | WSB1 |
| NDUFAB1 | TAS2R5 | RPS27A | KCTD9 | WWTR1 |
| NDUFB1 | TBRG4 | RPS28 | KIAA0556 | XPNPEP1 |
| NDUFB3 | TCEA3 | RPS3 | KIAA0753 | XPO4 |
| NDUFB4 | TCEB2 | RPS4X | KIAA0922 | XPR1 |
| NDUFB5 | TCF15 | RPS5 | KIAA1024 | YAP1 |
| NDUFB6 | TESC | RPS8 | KIAA1161 | YEATS2 |
| NDUFB8 | THAP1 | RPUSD3 | KIAA1191 | YPEL2 |
| NDUFB9 | TJP2 | RSAD1 | KIAA1244 | YWHAB |
| NDUFS4 | TKTL1 | RUFY1 | KIAA1539 | YWHAQ |
| NECAP1 | TLR7 | RUNX3 | KIAA1826 | ZBTB47 |
| NEGR1 | TMCO6 | S100A1 | KIAA2013 | ZC3H11A |
| NGFRAP1 | TMEM121 | S1PR3 | KIAA2026 | ZC3H14 |
| NHLRC2 | TMEM132B | SCN1A | KIDINS220 | ZC3H7A |
| NKAP | TMEM132C | SCUBE3 | KIF13A | ZC4H2 |
| NKIRAS1 | TMEM177 | SDHA | KIF1A | ZCCHC24 |
| NNT | TMEM208 | SDK1 | KIF20A | ZDHHC23 |
| NR2C2 | TMEM220 | SEC14L5 | KIF23 | ZDHHC3 |
| NR3C2 | TPRKB | SEC16B | KIF3B | ZEB1 |
| NRIP1 | TPSAB1 | SELENBP1 | KIF4A | ZFP106 |
| NRXN1 | TRIM36 | SEMA6D | KIRREL |  |
| NSL1 | TRIM7 | SEPP1 | KLHL12 |  |
| NUDT19 | TSGA14 | SGSM1 | KLHL2 |  |
| NUDT4 | TXNDC3 | SH2D1B | KLHL36 |  |
| NUFIP2 | UCKL1 | SH3KBP1 | KLHL5 |  |
| NUP43 | UMPS | SH3RF2 | KLHL6 |  |
| NUS1 | UQCRC1 | SHISA3 | KP2 |  |
| OCRL | VPREB3 | SIL1 | KP4 |  |
| OLFM4 | VRK2 | SIRT4 | KP6 |  |
| OTUD6B | VWA3A | SLC10A1 | KREMEN1 |  |
| P1L2 | WDR62 | SLC13A3 | LAMA2 |  |
| P2RY14 | WDSUB1 | SLC25A26 | LAMB1 |  |
| PAQR5 | WNT5A | SLC26A1 | LARP4 |  |
| PARG | WNT5B | SLC26A9 | LBH |  |
| PATZ1 | WSCD2 | SLC27A1 | LCLAT1 |  |
| PCDHB3 | ZBED5 | SLC29A1 | LCN12 |  |
| PCF11 | ZCCHC4 | SLC29A2 | LCOR |  |
| PCMT1 | ZCCHC8 | SLC2A9 | LEF1 |  |
| PCTK2 | ZDHHC11 | SLC5A1 | LGALS9 |  |
| PDE12 | ZFP2 | SLCO3A1 | LGI2 |  |
| PDE7B | ZNF101 | SLIT2 | LHFP |  |
| PDSS2 | ZNF296 | SLIT3 | LHFPL1 |  |
| PEBP4 | ZNF3 | SMARCD3 | LHPP |  |
| PEG3 | ZNF32 | SMTNL2 | LMAN2L |  |
| PFAS | ZNF335 | SI3 | LMBRD2 |  |
| PFKFB3 | ZNF33A | SPC5 | LNP1 |  |
| PGK1 | ZNF33B | SNHG6 | LNPEP |  |
| PGRMC1 | ZNF419 | SNRNP25 | LOXL1 |  |
| PHACTR3 | ZNF510 | SNRNP35 | LOXL2 |  |
| PI15 | ZNF551 | SNRNP70 | LOXL4 |  |
| PIGM | ZNF567 | SOX10 | LRCH1 |  |
| PIGW | ZNF585A | SOX15 | LRP6 |  |
| PIGY | ZNF674 | SPAG7 | LRRC20 |  |
| PIM3 | ZNF763 | SPATA5L1 | LRRC37A3 |  |
| PION | ZNF784 | SPATC1 | LRRC3B |  |
| PIP5K1B | ZNHIT2 | SPHK2 | LRRC41 |  |
| PITPNM2 | ZNHIT3 | SPHKAP | LRRFIP1 |  |
| PKIA | ZXDC | STAG1 | LSM1 |  |
| PLA2G4F | 42805 (MARCHF11)* | STARD10 | LTBP2 |  |
| PLAG1 |  | STARD3 | LUM |  |
| PLCL1 |  | STEAP4 | LUZP1 |  |
| PLCL2 |  | STOML2 | LYNX1 |  |
| PLDN |  | STXBP6 | LYPD1 |  |
| PLEKHF2 |  | SUMF1 |  |  |
| PLN |  | SUSD4 |  |  |
| PMP22 |  | SYCP3 |  |  |
| PMPCB |  | TAS2R5 |  |  |
| PNMA6A |  | TBX6 |  |  |
| PODXL |  | TC2N |  |  |
| POLR1D |  | TCEA3 |  |  |
| POLR2I |  | TCF15 |  |  |
| POLR3B |  | TESC |  |  |
| PPP1CB |  | THAP1 |  |  |
| PPP1R15B |  | THAP4 |  |  |
| PPP1R1A |  | THNSL1 |  |  |
| PPP2R3A |  | TIFA |  |  |
| PPP4R2 |  | TIGD1 |  |  |
| PPP6C |  | TIMP3 |  |  |
| PRELID2 |  | TJP2 |  |  |
| PRKRA |  | TKT |  |  |
| PRNP |  | TKTL1 |  |  |
| PRRC1 |  | TMCO7 |  |  |
| PRRG1 |  | TMEM121 |  |  |
| PS1TP4 |  | TMEM132B |  |  |
| PSMA3 |  | TMEM132C |  |  |
| PSMA8 |  | TMEM143 |  |  |
| PSMF1 |  | TMEM208 |  |  |
| PTBP2 |  | TMEM220 |  |  |
| PTCH1 |  | TMEM27 |  |  |
| PTDSS1 |  | TMEM85 |  |  |
| PTGDR |  | TMOD2 |  |  |
| PTGER4 |  | TMTC1 |  |  |
| PTGES3 |  | TOX |  |  |
| PTGR2 |  | TPCN1 |  |  |
| PTP4A2 |  | TPSAB1 |  |  |
| PTPRO |  | TRABD |  |  |
| QKI |  | TRAC |  |  |
| RAB11FIP2 |  | TRAP1 |  |  |
| RAB12 |  | TRIM16 |  |  |
| RAB21 |  | TRIM16L |  |  |
| RAB33B |  | TRIM4 |  |  |
| RABEPK |  | TRIM7 |  |  |
| RABGGTB |  | TRPM7 |  |  |
| RAD23B |  | TSPAN7 |  |  |
| RAD9B |  | TXNDC15 |  |  |
| RAF1 |  | UBAP2 |  |  |
| RAP2C |  | UBXN1 |  |  |
| RASD2 |  | UCKL1 |  |  |
| RB1CC1 |  | UHRF2 |  |  |
| RBL2 |  | UPF3A |  |  |
| RBM12 |  | UQCRC1 |  |  |
| RBM12B |  | VEZF1 |  |  |
| RBM20 |  | VIPR1 |  |  |
| RBMX |  | VIT |  |  |
| RCHY1 |  | VPREB3 |  |  |
| RET |  | VWA3A |  |  |
| RFXAP |  | WDR62 |  |  |
| RG9MTD2 |  | WDSUB1 |  |  |
| RHBDD1 |  | WNT5A |  |  |
| RHOT1 |  | WNT5B |  |  |
| RICS |  | WSCD2 |  |  |
| RIOK2 |  | ZACN |  |  |
| RNF114 |  | ZBTB40 |  |  |
| RNF149 |  | ZCCHC11 |  |  |
| RNF165 |  | ZFP2 |  |  |
| RNF2 |  | ZNF135 |  |  |
| RNLS |  | ZNF225 |  |  |
| RORC |  | ZNF33A |  |  |
| RP11-138L21.1 |  | ZNF33B |  |  |
| RP2 |  | ZNF418 |  |  |
| RPAIN |  | ZNF497 |  |  |
| RPL14 |  | ZNF502 |  |  |
| RPL15 |  | ZNF521 |  |  |
| RPL21 |  | ZNF540 |  |  |
| RPL24 |  | ZNF567 |  |  |
| RPL27 |  | ZNF674 |  |  |
| RPL31 |  | ZNF74 |  |  |
| RPL37A |  | ZNF784 |  |  |
| RPL41 |  | ZNF831 |  |  |
| RPL7L1 |  | ZNF837 |  |  |
| RPL9 |  | ZNF843 |  |  |
| RPLP2P1 |  | ZNHIT3 |  |  |
| RPS10P5 |  | ZXDC |  |  |
| RPS11 |  | 42805 (MARCHF11)* |  |  |
| RPS13 |  | CPT1B |  |  |
| RPS17P5 |  |  |  |  |
| RPS25 |  |  |  |  |
| RPS3A |  |  |  |  |
| RPS4X |  |  |  |  |
| RRM2B |  |  |  |  |
| RRP15 |  |  |  |  |
| RSBN1L |  |  |  |  |
| RSU1 |  |  |  |  |
| RUNDC3B |  |  |  |  |
| RWDD4A |  |  |  |  |
| RYR2 |  |  |  |  |
| SAMD3 |  |  |  |  |
| SAMD8 |  |  |  |  |
| SCGB1D2 |  |  |  |  |
| SCGB2A2 |  |  |  |  |
| SCML2 |  |  |  |  |
| SCN1A |  |  |  |  |
| SCN7A |  |  |  |  |
| SDCBP |  |  |  |  |
| SDHC |  |  |  |  |
| SDK1 |  |  |  |  |
| SDR39U1 |  |  |  |  |
| SEC61B |  |  |  |  |
| SEC62 |  |  |  |  |
| SEPP1 |  |  |  |  |
| SERF1A |  |  |  |  |
| SFPQ |  |  |  |  |
| SFRS11 |  |  |  |  |
| SFRS12IP1 |  |  |  |  |
| SFRS3 |  |  |  |  |
| SFT2D1 |  |  |  |  |
| SGCG |  |  |  |  |
| SGMS1 |  |  |  |  |
| SGPP1 |  |  |  |  |
| SH3KBP1 |  |  |  |  |
| SHISA3 |  |  |  |  |
| SHPRH |  |  |  |  |
| SIKE1 |  |  |  |  |
| SIRT1 |  |  |  |  |
| SIRT4 |  |  |  |  |
| SKP1 |  |  |  |  |
| SLAMF6 |  |  |  |  |
| SLC15A4 |  |  |  |  |
| SLC16A7 |  |  |  |  |
| SLC19A2 |  |  |  |  |
| SLC1A3 |  |  |  |  |
| SLC22A3 |  |  |  |  |
| SLC25A21 |  |  |  |  |
| SLC26A9 |  |  |  |  |
| SLC27A6 |  |  |  |  |
| SLC30A5 |  |  |  |  |
| SLC35F1 |  |  |  |  |
| SLC38A2 |  |  |  |  |
| SLC5A1 |  |  |  |  |
| SLC7A6OS |  |  |  |  |
| SLC9A6 |  |  |  |  |
| SLTM |  |  |  |  |
| SMAD4 |  |  |  |  |
| SMNDC1 |  |  |  |  |
| SMURF2 |  |  |  |  |
| SNHG6 |  |  |  |  |
| SNRNP27 |  |  |  |  |
| SNX3 |  |  |  |  |
| SNX31 |  |  |  |  |
| SOAT1 |  |  |  |  |
| SOCS5 |  |  |  |  |
| SOX2OT |  |  |  |  |
| SP140 |  |  |  |  |
| SP3 |  |  |  |  |
| SP4 |  |  |  |  |
| SPATA2 |  |  |  |  |
| SPATA5 |  |  |  |  |
| SPDYE1 |  |  |  |  |
| SPHKAP |  |  |  |  |
| SPINK7 |  |  |  |  |
| SPTY2D1 |  |  |  |  |
| SRI |  |  |  |  |
| SRP9 |  |  |  |  |
| STAG1 |  |  |  |  |
| STEAP2 |  |  |  |  |
| STT3B |  |  |  |  |
| STXBP1 |  |  |  |  |
| SUCLA2 |  |  |  |  |
| SUMO1 |  |  |  |  |
| SUMO2 |  |  |  |  |
| SUZ12P |  |  |  |  |
| SYCP3 |  |  |  |  |
| SYT3 |  |  |  |  |
| TAF5 |  |  |  |  |
| TAPT1 |  |  |  |  |
| TARDBP |  |  |  |  |
| TBC1D4 |  |  |  |  |
| TC2N |  |  |  |  |
| TCEAL1 |  |  |  |  |
| TCEB3 |  |  |  |  |
| TGFBR1 |  |  |  |  |
| THAP1 |  |  |  |  |
| THBD |  |  |  |  |
| THNSL1 |  |  |  |  |
| THUMPD1 |  |  |  |  |
| TIFA |  |  |  |  |
| TINP1 |  |  |  |  |
| TIRAP |  |  |  |  |
| TJP2 |  |  |  |  |
| TKTL1 |  |  |  |  |
| TM2D1 |  |  |  |  |
| TM2D3 |  |  |  |  |
| TMBIM4 |  |  |  |  |
| TMED6 |  |  |  |  |
| TMEM106B |  |  |  |  |
| TMEM123 |  |  |  |  |
| TMEM128 |  |  |  |  |
| TMEM132C |  |  |  |  |
| TMEM182 |  |  |  |  |
| TMEM27 |  |  |  |  |
| TMEM41B |  |  |  |  |
| TMEM70 |  |  |  |  |
| TMEM85 |  |  |  |  |
| TOX |  |  |  |  |
| TPRKB |  |  |  |  |
| TPSAB1 |  |  |  |  |
| TPSB2 |  |  |  |  |
| TPT1 |  |  |  |  |
| TRA2B |  |  |  |  |
| TRAC |  |  |  |  |
| TRDN |  |  |  |  |
| TRIM23 |  |  |  |  |
| TRMT61B |  |  |  |  |
| TSHZ1 |  |  |  |  |
| TSPYL1 |  |  |  |  |
| TSPYL4 |  |  |  |  |
| TSPYL5 |  |  |  |  |
| TSX |  |  |  |  |
| TTC31 |  |  |  |  |
| TTC33 |  |  |  |  |
| TTC7B |  |  |  |  |
| TUBA3D |  |  |  |  |
| TUBD1 |  |  |  |  |
| TUSC1 |  |  |  |  |
| TXLNB |  |  |  |  |
| TXNL1 |  |  |  |  |
| UBA5 |  |  |  |  |
| UBE2A |  |  |  |  |
| UBE2B |  |  |  |  |
| UBE2D1 |  |  |  |  |
| UBE2E3 |  |  |  |  |
| UBE2K |  |  |  |  |
| UBL3 |  |  |  |  |
| UBR3 |  |  |  |  |
| UCKL1 |  |  |  |  |
| UEVLD |  |  |  |  |
| UGDH |  |  |  |  |
| UHRF2 |  |  |  |  |
| UQCRB |  |  |  |  |
| UQCRC2 |  |  |  |  |
| UQCRFS1 |  |  |  |  |
| USP51 |  |  |  |  |
| VAMP4 |  |  |  |  |
| VBP1 |  |  |  |  |
| VDAC2 |  |  |  |  |
| VIT |  |  |  |  |
| VKORC1L1 |  |  |  |  |
| VRK2 |  |  |  |  |
| VWA3A |  |  |  |  |
| WDR51B |  |  |  |  |
| WDR61 |  |  |  |  |
| WDR82 |  |  |  |  |
| WDSUB1 |  |  |  |  |
| WHAMML2 |  |  |  |  |
| WNT5A |  |  |  |  |
| WRB |  |  |  |  |
| WWOX |  |  |  |  |
| WWP1 |  |  |  |  |
| XBP1 |  |  |  |  |
| XCL1 |  |  |  |  |
| XKR4 |  |  |  |  |
| XYLT1 |  |  |  |  |
| YEATS4 |  |  |  |  |
| YME1L1 |  |  |  |  |
| YTHDC2 |  |  |  |  |
| YWHAG |  |  |  |  |
| ZADH2 |  |  |  |  |
| ZBED5 |  |  |  |  |
| ZBTB1 |  |  |  |  |
| ZBTB10 |  |  |  |  |
| ZBTB11 |  |  |  |  |
| ZBTB26 |  |  |  |  |
| ZBTB39 |  |  |  |  |
| ZBTB44 |  |  |  |  |
| ZC3H6 |  |  |  |  |
| ZCCHC2 |  |  |  |  |
| ZDHHC17 |  |  |  |  |
| ZEB2 |  |  |  |  |
| ZFP112 |  |  |  |  |
| ZFP37 |  |  |  |  |
| ZMPSTE24 |  |  |  |  |
| ZNF138 |  |  |  |  |
| ZNF14 |  |  |  |  |
| ZNF140 |  |  |  |  |
| ZNF146 |  |  |  |  |
| ZNF17 |  |  |  |  |
| ZNF2 |  |  |  |  |
| ZNF200 |  |  |  |  |
| ZNF214 |  |  |  |  |
| ZNF236 |  |  |  |  |
| ZNF25 |  |  |  |  |
| ZNF252 |  |  |  |  |
| ZNF253 |  |  |  |  |
| ZNF260 |  |  |  |  |
| ZNF267 |  |  |  |  |
| ZNF271 |  |  |  |  |
| ZNF273 |  |  |  |  |
| ZNF302 |  |  |  |  |
| ZNF319 |  |  |  |  |
| ZNF322A |  |  |  |  |
| ZNF33B |  |  |  |  |
| ZNF354B |  |  |  |  |
| ZNF382 |  |  |  |  |
| ZNF383 |  |  |  |  |
| ZNF441 |  |  |  |  |
| ZNF468 |  |  |  |  |
| ZNF493 |  |  |  |  |
| ZNF529 |  |  |  |  |
| ZNF548 |  |  |  |  |
| ZNF578 |  |  |  |  |
| ZNF585A |  |  |  |  |
| ZNF594 |  |  |  |  |
| ZNF623 |  |  |  |  |
| ZNF670 |  |  |  |  |
| ZNF706 |  |  |  |  |
| ZNF765 |  |  |  |  |
| ZNF781 |  |  |  |  |
| ZNF823 |  |  |  |  |
| ZNF828 |  |  |  |  |
| ZNF835 |  |  |  |  |
| ZNF862 |  |  |  |  |
| ZNF92 |  |  |  |  |

*****Listed unclassified due to transcript originally coded as a number
