# Supplementary material for: Correction: Sequential analysis of myocardial gene expression with phenotypic change: Use of cross-platform concordance to strengthen biologic relevance
Source: PLoS One. 2019 Oct 22;14(10):e0224389. doi: 10.1371/journal.pone.0224389 (PMC6804972; doi:10.1371/journal.pone.0224389)
Supplement: S4 Table — (DOCX) [file pone.0224389.s004.docx]

**S4 Table. Up or downregulated genes within the R and R/NR analyses, microarray measurements in the *A-S* cohort.**

| **Upregulated Genes** | | | | **Downregulated Genes** | | | |
| --- | --- | --- | --- | --- | --- | --- | --- |
| A2BP1 | E2F5 | MADD | S100PBP | AAA1 | E2F1 | M6PR | S100A11 |
| ABCA11P | EAPP | MAFB | SAFB2 | AADACL2 | E2F2 | MACF1 | S100A2 |
| ABCA17P | EBAG9 | MAGEE1 | SALL2 | AAGAB | E2F3 | MAD2L1 | S100A5 |
| ABCA6 | EBF1 | MAGEF1 | SAMD13 | AARS2 | EAF2 | MAD2L2 | S1PR2 |
| ABCA8 | ECH1 | MAGEH1 | SAMD3 | AATF | EBP | MAFF | SACS |
| ABCA9 | ECHDC2 | MAGI2 | SASH3 | ABAT | ECE2 | MAFK | SAE1 |
| ABCB10 | ECHDC3 | MAGOH | SAT2 | ABCA7 | ECT2 | MAGEA4 | SAMD14 |
| ABCB6 | EEF2K | MAGOH2 | SBF2 | ABCC4 | EDEM2 | MAGEA8 | SAP18 |
| ABCC6 | EEPD1 | MAGOHB | SC4MOL | ABCD1 | EDN1 | MAGED2 | SARDH |
| ABCC9 | EFCAB2 | MAMDC2 | SCAI | ABCG1 | EEF1A1 | MAGEL2 | SARM1 |
| ABCD2 | EFCAB7 | MAML1 | SCAPER | ABCG2 | EF5 | MAGIX | SBNO1 |
| ABHD10 | EFHA1 | MAN1C1 | SCARA5 | ABCG4 | EGLN1 | MALL | SCAMP3 |
| ABHD14A | EFHA2 | MAN2A2 | SCARF1 | ABHD12 | EGLN3 | MANBAL | SCAMP4 |
| ABHD3 | EFHC2 | MANBA | SCAR15 | ABHD2 | EGR1 | MAP1A | SCAMP5 |
| ACAA2 | EFHD1 | MAP2K6 | SCD5 | ABHD5 | EGR2 | MAP1B | SCCPDH |
| ACACB | EFS | MAP3K14 | SCGB1D2 | ABI2 | EGR3 | MAP1LC3A | SCEL |
| ACAD8 | EGF | MAP3K5 | SCHIP1 | ABRA | EHBP1L1 | MAP1LC3C | SCFD2 |
| ACAD9 | EGFLAM | MAP3K8 | SCML1 | ACAD11 | EID3 | MAP2 | SCG2 |
| ACADM | EGFR | MAPKAPK3 | SCN1A | ACBD3 | EIF2C3 | MAP2K3 | SCG5 |
| ACADSB | EI24 | MAPKSP1 | SCN9A | ACCN1 | EIF4A1 | MAP2K5 | SCLY |
| ACADVL | EID1 | MAPT | SCP2 | ACCN5 | EIF4E2 | MAP3K13 | SCMH1 |
| ACAP2 | EID2B | 42804 (MARCHF10)* | SCPEP1 | ACE2 | EIF5A2 | MAP3K3 | SCN2B |
| ACAT1 | EIF1AX | 42805 (MARCHF11)* | SCUBE3 | ACER1 | EIF5B | MAP3K6 | SCN3B |
| ACCN3 | EIF2A | MBD4 | SCYL3 | ACLY | EIF6 | MAP3K7 | SCNN1G |
| ACN9 | EIF3J | MBD5 | SDCBP | ACOT7 | ELL3 | MAP3K9 | SCRG1 |
| ACOX2 | EIF3L | MBD6 | SDHA | ACOT8 | ELMOD1 | MAP4 | SCRN1 |
| ACSL1 | EIF4A3 | MBIP | SDHALP1 | ACOT9 | ELN | MAP7 | SCUBE2 |
| ACSS2 | EIF4EBP2 | MBLAC2 | SDHC | ACOXL | ELOVL5 | MAP9 | SDAD1 |
| ACSS3 | EIF5 | MBOAT1 | SDHD | ACSBG1 | ELOVL6 | MAPK1 | SDC1 |
| ACVR2A | ELF2 | MCC | SDK1 | ACTA1 | ELP3 | MAPK11 | SDC2 |
| ADA | EMCN | MCCC1 | SDR39U1 | ACTN1 | EME1 | MAPK12 | SDC3 |
| ADAM11 | EML1 | MCEE | SEC14L5 | ACTN2 | EMILIN3 | MAPK15 | SDC4 |
| ADAM23 | EMP2 | MCF2 | SEC16B | ACTR1A | EML2 | MAPKAPK5 | SDF4 |
| ADAM33 | ENOSF1 | MCM3APAS | SEC23B | ACTR8 | EML4 | MAPRE1 | SDK2 |
| ADAM8 | ENPP2 | MCM9 | SEC31B | ADAM18 | EMP3 | MAPRE2 | SDR42E1 |
| ADAMTS5 | ENPP4 | MCPH1 | SEC61B | ADAM19 | EH | 42796 (MARCHF2)* | SDSL |
| ADAMTS6 | ENTPD2 | MCTS1 | SECISBP2 | ADAMTS14 | EM | 42797 (MARCHF3)* | SEC22C |
| ADAMTS7 | EOMES | MDH1B | SECTM1 | ADAMTS2 | ENC1 | 42798 (MARCHF4)* | SEC24A |
| ADCK4 | EP300 | MDM1 | SELENBP1 | ADAMTS9 | ENO2 | MARCKSL1 | SEC31A |
| ADCY7 | EPC1 | ME1 | SELL | ADAMTSL5 | ENO3 | MARVELD2 | SEC61A1 |
| ADCY9 | EPC2 | ME2 | SEMA3A | ADAP1 | ENOX2 | MASP1 | SEL1L2 |
| ADCYAP1R1 | EPDR1 | MECP2 | SEMA3C | ADAT1 | ENTHD1 | MAST3 | SELI |
| ADD3 | EPHX1 | MED31 | SEMA4B | ADCY6 | ENTPD7 | MAT2A | SELM |
| ADH1B | EPN3 | MED4 | SEMA4G | ADCYAP1 | EPAG | MBOAT2 | SELS |
| ADH1C | EPS15 | MEGF9 | SEMA6A | ADH4 | EPB41L1 | MBOAT7 | SEMA3F |
| ADHFE1 | EPS8 | MEIS2 | SEMA6C | ADORA3 | EPHA2 | MCAM | SEMA4A |
| ADK | ERAP2 | METTL7A | SEMA6D | ADRA1D | EPR1 | MCART6 | SEMA6B |
| ADORA1 | ERBB2 | METTL7B | SENP6 | ADRA2C | EPS8L1 | MCL1 | SENP5 |
| ADRA1A | ERBB3 | MFF | SEPP1 | ADRBK2 | ERC1 | MCM10 | SEPN1 |
| ADRB1 | ERLIN2 | MFSD11 | 42991 (SEPTIN7P2)* | AEBP1 | ERC2 | MCM2 | 42989 (SEPTIN11)* |
| ADRB2 | ESD | MFSD8 | SEPW1 | AEN | ERCC6L | MCM6 | 42980 (SEPTIN2)* |
| AEBP2 | ESRRG | MGC12488 | SERF1A | AFF4 | ERMP1 | MDFIC | 42982 (SEPTIN4)* |
| AFF3 | ETFDH | MGC16075 | SERINC1 | AFFX-PheX-5_at | ERO1L | MDK | 42984 (SEPTIN6)* |
| AFTPH | EXOSC3 | MGC23284 | SERPING1 | AFFX-PheX-M_at | ESCO2 | MED19 | 42986 (SEPTIN8)* |
| AGA | EXOSC8 | MGC29506 | SERTAD2 | AFFX-r2-Bs-phe-3_at | ESF1 | MED27 | SEPX1 |
| AGAP1 | EXPH5 | MGC9913 | SESN1 | AGBL5 | ESPL1 | MEG3 | SERGEF |
| AGBL3 | EXTL2 | MGST1 | SETD1B | AGER | ESRP1 | MEGF8 | SERINC2 |
| AGTPBP1 | EZH1 | MICAL3 | SFRS1 | AGFG2 | ESRRB | MELK | SERPINB8 |
| AGTR1 | FADS1 | MID1IP1 | SFRS11 | AGR2 | ESYT3 | MEMO1 | SERPIND1 |
| AGXT2L1 | FAIM | MITD1 | SFRS17A | AGRN | ETF1 | MEOX1 | SERPINE1 |
| AHCYL2 | FAM104B | MKRN1 | SFRS7 | AGTRAP | ETV1 | METRN | SERPINE2 |
| AIDA | FAM108B1 | MLF1 | SFT2D3 | AHSA1 | ETV4 | METTL1 | SERPINH1 |
| AIMP1 | FAM116A | MLLT10 | SGK493 | AICDA | ETV5 | METTL2B | SERPINI1 |
| AKAP7 | FAM118A | MLPH | SGMS1 | AIPL1 | EXO1 | MEX3D | SERTAD3 |
| AKAP8 | FAM119A | MMAB | SGSM1 | AK1 | EXOC2 | MFAP1 | SESN3 |
| AK | FAM122A | MMADHC | SH3BP2 | AK7 | EXOC3L2 | MFAP2 | SETD3 |
| AKR7A2 | FAM124A | MOBKL2B | SH3KBP1 | AKAP13 | EXOC6B | MFAP3L | SETD7 |
| AKR7A3 | FAM126B | MOCS2 | SH3RF3 | AKAP5 | EXOSC1 | MFAP5 | SEZ6L |
| ALDH1A1 | FAM127B | MORC3 | SH3TC2 | AKAP6 | EXOSC4 | MFGE8 | SEZ6L2 |
| ALDH2 | FAM128A | MORF4L1 | SH3YL1 | AKIRIN2 | EXT1 | MFI2 | SF3B2 |
| ALDH8A1 | FAM128B | MOSC2 | SHISA3 | AKTIP | EXTL3 | MFSD7 | SFRP4 |
| ALDOA | FAM129C | MPL | SHPK | ALCAM | EZH2 | MGAT2 | SFRS2IP |
| ALDOC | FAM13AOS | MPP3 | SHPRH | ALDH18A1 | EZR | MGAT5 | SFRS6 |
| ALG10B | FAM149A | MREG | SIGLEC7 | ALDH1B1 | F13B | MGC12982 | SFTA1P |
| ALG2 | FAM160B1 | MRI1 | SIP1 | ALDH1L1 | F2 | MGC15705 | SFXN3 |
| ALG6 | FAM162A | MRO | SIPA1L2 | ALDH3A2 | F2R | MGC16384 | SFXN4 |
| ALMS1 | FAM162B | MRP63 | SIRT1 | ALDOB | F2RL1 | MGC2752 | SGCD |
| ALS2CL | FAM168A | MRPL16 | SIRT4 | ALG14 | F2RL2 | MGC39545 | SGIP1 |
| ALS2CR10 | FAM175A | MRPL22 | SIRT5 | ALKBH5 | FABP3 | MGC4294 | SGK1 |
| ALS2CR11 | FAM178B | MRPL24 | SKP1 | ALOXE3 | FABP6 | MGMT | SGK269 |
| AMD1 | FAM179A | MRPL3 | SLC11A2 | ALPK3 | FAH | MGP | SGTA |
| AMDHD1 | FAM179B | MRPL30 | SLC12A6 | ALPL | FAM101B | MGRN1 | SH2D4A |
| AMMECR1L | FAM189A2 | MRPL32 | SLC15A2 | ALS2 | FAM105B | MICAL2 | SH3BGR |
| AMN1 | FAM18B | MRPL33 | SLC16A7 | ALS2CR12 | FAM109B | MICALL2 | SH3BGRL3 |
| AMY2B | FAM36A | MRPL39 | SLC22A3 | ALS2CR4 | FAM110B | MIER2 | SH3BP5L |
| ANG | FAM3B | MRPL43 | SLC25A24 | AMAC1 | FAM114A1 | MIF | SH3D19 |
| ANGPT1 | FAM40B | MRPL48 | SLC25A26 | AMFR | FAM115C | MIIP | SH3GL2 |
| ANGPTL7 | FAM46A | MRPL51 | SLC25A3 | AMOTL1 | FAM118B | MI | SH3GL3 |
| ANK1 | FAM46C | MRPL9 | SLC25A36 | AMPH | FAM12B | MIP | SH3GLB1 |
| ANKHD1 | FAM50B | MRPS15 | SLC26A4 | ANGPT2 | FAM149B1 | MKI67 | SH3PXD2A |
| ANKRA2 | FAM59A | MRPS18C | SLC26A9 | ANGPTL2 | FAM166B | MKI67IP | SHB |
| ANKRD13C | FAM63A | MRPS24 | SLC27A6 | ANKRD1 | FAM167A | MKNK1 | SHISA5 |
| ANKRD2 | FAM65C | MRPS25 | SLC29A1 | ANKRD34A | FAM167B | MKRN2 | SHQ1 |
| ANKRD23 | FAM70A | MRPS31 | SLC29A2 | ANKRD34C | FAM169A | MLEC | SHROOM2 |
| ANKRD27 | FAM76B | MSL1 | SLC2A5 | ANKS1B | FAM177A1 | MLF1IP | SHROOM3 |
| ANKRD28 | FAM78A | MT1H | SLC31A2 | ANKS6 | FAM187B | MLH1 | SHROOM4 |
| ANKRD29 | FAM81A | MT1P2 | SLC35A1 | ANLN | FAM22F | MLLT11 | SI |
| ANKRD46 | FAM81B | MT1X | SLC35A3 | ANO7 | FAM32A | MMD2 | SIAH2 |
| ANKRD6 | FAM8A1 | MT3 | SLC35D3 | ANP32A | FAM45B | MME | SIK2 |
| ANO5 | FAM96A | MTERF | SLC35F1 | ANTXR1 | FAM47B | MMP1 | SILV |
| AOC2 | FAM98B | MTERFD1 | SLC37A4 | ANXA11 | FAM54A | MMP19 | SIPA1L3 |
| AOC3 | FANCE | MTERFD2 | SLC38A4 | ANXA2 | FAM57A | MND1 | SIRPA |
| APLN | FAS | MTF2 | SLC40A1 | ANXA2P3 | FAM64A | MNS1 | SIRT6 |
| APOB48R | FASTKD3 | MTHFD2 | SLC41A1 | AP1G1 | FAM7A3 | MNX1 | SKA1 |
| APOBEC2 | FBXL3 | MTHFD2L | SLC43A1 | AP1M1 | FAM83D | MOBKL2C | SKA3 |
| APOD | FBXL4 | MTMR14 | SLC44A3 | AP1S3 | FAM83G | MORN4 | SKIL |
| APOL3 | FBXL5 | MTMR6 | SLC46A3 | AP2M1 | FAM83H | MOXD1 | SLAIN2 |
| APPL1 | FBXO3 | MTO1 | SLC5A1 | AP3M2 | FANCA | MPHOSPH8 | SLC12A1 |
| AQP4 | FBXO40 | MTR | SLC5A7 | AP4S1 | FANCI | MPND | SLC12A2 |
| AQP7 | FBXO42 | MTRF1 | SLC6A1 | APAF1 | FAP | MPRIP | SLC12A9 |
| ARAP2 | FBXO46 | MTSS1 | SLC6A16 | APLP1 | FARP1 | MPV17 | SLC15A1 |
| ARGLU1 | FCRL3 | MTTP | SLC7A6OS | APLP2 | FBN1 | MPV17L2 | SLC16A1 |
| ARHGAP12 | FDFT1 | MTUS1 | SLC9A9 | APOA1 | FBXL19 | MPZL1 | SLC16A9 |
| ARHGAP21 | FDPS | MUC20 | SLCO2B1 | APOBEC3B | FBXO16 | MRAP2 | SLC17A6 |
| ARHGAP6 | FDX1 | MUT | SLCO3A1 | APOBEC3F | FBXO22 | MRAS | SLC1A2 |
| ARHGAP9 | FEZ1 | MXI1 | SLIT2 | APOE | FBXO27 | MRPL20 | SLC1A4 |
| ARID3B | FGD3 | MYBPC1 | SLMO1 | APOL2 | FBXO43 | MRPL54 | SLC1A6 |
| ARIH2 | FGF12 | MYL3 | SLTM | APOL4 | FCGR1A | MRTO4 | SLC20A2 |
| ARL1 | FGF7 | MYLK4 | SLU7 | AQP10 | FCRLA | MSMB | SLC24A1 |
| ARL6IP6 | FGFBP2 | MYO7A | SMARCAD1 | ARCN1 | FDX1L | MSR1 | SLC25A14 |
| ARMCX1 | FGFBP3 | MYOF | SMEK2 | ARF3 | FER1L5 | MSRA | SLC25A23 |
| ARRDC4 | FGFR4 | MYST2 | SMO | ARG2 | FERD3L | MSRB3 | SLC25A25 |
| ART3 | FHIT | MYST4 | SMR3B | ARHGAP1 | FERMT2 | MSTO1 | SLC25A35 |
| ART5 | FHL2 | MZF1 | SMTN | ARHGAP11A | FETUB | MTF1 | SLC25A5 |
| ASAH1 | FIGF | N4BP1 | SMTNL2 | ARHGAP23 | FEV | MTHFD1L | SLC27A5 |
| ASB10 | FIGN | N4BP2 | SI3 | ARHGDIB | FEZ2 | MTHFSD | SLC2A3 |
| ASB12 | FITM1 | N4BP2L1 | SP91 | ARHGDIG | FGB | MTL5 | SLC2A6 |
| ASB14 | FKBP7 | N4BP2L2 | SPC5 | ARHGEF6 | FGF1 | MTMR3 | SLC30A4 |
| ASB15 | FKBP9 | N6AMT2 | SNED1 | ARHGEF9 | FGF5 | MTMR8 | SLC31A1 |
| ASB4 | FKTN | ALAD2 | SNHG10 | ARID5A | FGFR1 | MTP18 | SLC34A1 |
| ASB5 | FLJ10038 | CA2 | SNHG12 | ARID5B | FGFR1OP | MUC1 | SLC35C1 |
| ASB8 | FLJ25076 | E1 | SNHG5 | ARL4A | FGFRL1 | MUL1 | SLC35E1 |
| ASNSD1 | FLJ33360 | F1 | SNHG6 | ARL4C | FHAD1 | MUM1 | SLC35F2 |
| ASPA | FLJ36644 | GPA | SNHG9 | ARMC9 | FHL1 | MXRA5 | SLC35F5 |
| ASPH | FLJ38717 | GS | SNORD104 | ARNTL2 | FIBIN | MXRA7 | SLC38A7 |
| ASPSCR1 | FLJ39639 | MPT | SNRNP200 | ARP11 | FIBP | MYADM | SLC39A4 |
| ATF6B | FLJ39653 | NOG | SNRNP27 | ARPC4 | FILIP1 | MYBBP1A | SLC39A9 |
| ATF7IP | FLJ41455 | P1L2 | SNRNP48 | ARPC5 | FIP1L1 | MYBL1 | SLC3A1 |
| ATP11C | FLJ42875 | PEPLD | SNRPC | ARPP-21 | FJX1 | MYEF2 | SLC43A3 |
| ATP1B2 | FLJ45244 | RG1L | SNRPF | ARSB | FKBP10 | MYH10 | SLC4A8 |
| ATP5C1 | FLT1 | RS2 | SNX15 | ARSD | FKBP14 | MYL6 | SLC6A15 |
| ATP5G1 | FLT3LG | T11 | SNX2 | ARSJ | FKSG73 | MYL9 | SLC6A6 |
| ATP5G3 | FMO2 | T6 | SNX3 | ASAP1 | FLG | MYO16 | SLC7A1 |
| ATP5I | FNBP4 | NBN | SON | ASCC3 | FLJ13439 | MYO18B | SLC8A1 |
| ATP5J | FNDC5 | NCEH1 | SORCS1 | ASF1B | FLJ16124 | MYO5B | SLC9A1 |
| ATP5J2 | FNIP1 | NCKAP5 | SORCS2 | ASIP | FLJ30838 | MYOZ3 | SLC9A5 |
| ATP8B4 | FOXD3 | NCOA1 | SORL1 | AS1 | FLJ30901 | MYPN | SLCO1C1 |
| ATPBD4 | FOXO1 | NCOA4 | SOS1 | ASNS | FLJ31715 | CC1 | SLCO2A1 |
| ATPGD1 | FOXO4 | NCR3 | SOX10 | ASPM | FLJ34208 | CC2 | SLFN5 |
| ATPIF1 | FPGT | NCR00092 | SOX15 | ASPN | FLJ35024 | DSYN1 | SLITRK2 |
| AUH | FRAS1 | NCR00115 | SP140 | ATAD2 | FLJ35424 | NOS1 | SMAD2 |
| AUTS2 | FRAT1 | NCR00117 | SP3 | ATF3 | FLJ35934 | NOS2 | SMAD5OS |
| B3GALT2 | FRAT2 | NCR00161 | SPAG1 | ATF5 | FLJ39051 | P1L4 | SMAD6 |
| B3GALTL | FRG1 | NCR00181 | SPARCL1 | ATG12 | FLJ39080 | P1L5 | SMAD7 |
| B4GALNT1 | FRMD1 | NCR00201 | SPATA13 | ATG4D | FLJ41757 | PG | SMAD9 |
| BAI3 | FRMD4A | NCR00202 | SPATA5 | ATIC | FLJ43663 | T2 | SMAP2 |
| BATF2 | FRMD4B | NCR00203 | SPATA9 | ATL3 | FLJ90757 | T8B | SMARCA5 |
| BAZ2B | FRMD5 | ND2 | SPDYE1 | ATOX1 | FLNC | V2 | SMARCE1 |
| BBS1 | FRS3 | NDN | SPG20 | ATP10D | FMNL3 | NBPF5 | SMC4 |
| BBS10 | FSD2 | NDRG1 | SPG7 | ATP12A | FMO1 | NBR2 | SMOC2 |
| BCAR3 | FSHR | NDRG4 | SPHKAP | ATP13A3 | FMO4 | NCAPG | SMURF1 |
| BCDIN3D | FST | NDUFA1 | SPNS2 | ATP1A2 | FMO6P | NCAPH | SMYD1 |
| BCKDHA | FTHP1 | NDUFA12 | SPON2 | ATP1A4 | FMOD | NCDN | SMYD2 |
| BCL11A | FTO | NDUFA4 | SPRY2 | ATP1B3 | FN1 | NCR00105 | SP29 |
| BCL11B | FTSJD1 | NDUFAB1 | SR140 | ATP1B4 | FNDC3B | NCR00119 | SP47 |
| BCL2A1 | FUBP1 | NDUFB1 | SRBD1 | ATP2B3 | FNDC4 | NCR00189 | SNCA |
| BCL2L11 | FUNDC1 | NDUFB10 | SRI | ATP2B4 | FNDC8 | NDC80 | SNCAIP |
| BCL2L12 | FUNDC2 | NDUFB11 | SRP14 | ATP6V0A2 | FOSB | NDE1 | SNIP |
| BCL6 | FXN | NDUFB2 | SRP54 | ATP6V1D | FOSL2 | NECAB1 | SNIP1 |
| BCL7A | FYCO1 | NDUFB3 | SRP9 | ATP6V1E1 | FOXE1 | NECAB2 | SNN |
| BEND5 | FZD5 | NDUFB4 | SRRD | ATP6V1E2 | FOXK2 | NECAP2 | SNORA71B |
| BEND7 | G0S2 | NDUFB5 | SSBP1 | ATP6V1G1 | FOXM1 | NEFH | SNORD123 |
| BIN2 | GABARAPL2 | NDUFB6 | SSTR1 | ATP8A2 | FOXP2 | NEIL2 | SNRPD2 |
| BIRC2 | GABBR2 | NDUFB8 | ST13 | ATP8B3 | FOXS1 | NEIL3 | SNRPN |
| BIRC3 | GABRE | NDUFB9 | ST7 | ATRNL1 | FREQ | NEK1 | SNTB2 |
| BLM | GALNT12 | NDUFC2 | ST7OT1 | ATRX | FRZB | NEK2 | SNW1 |
| BLNK | GALNT8 | NDUFS3 | STAG1 | ATXN10 | FSCN1 | NENF | SNX11 |
| BLVRB | GALNTL1 | NDUFS4 | STARD9 | ATXN7L1 | FSCN2 | NES | SNX19 |
| BMF | GALNTL2 | NDUFV2 | STEAP2 | AURKA | FSD1L | NETO2 | SNX24 |
| BMI1 | GAS1 | NEDD1 | STEAP3 | AURKB | FSIP1 | NEU3 | SOCS2 |
| BMP2 | GAS2 | NEGR1 | STEAP4 | AZIN1 | FSTL3 | NEURL3 | SOCS4 |
| BMP5 | GBAS | NEK10 | STOX1 | B3GALNT2 | FUCA2 | NEUROD1 | SOHLH2 |
| BMP7 | GBP5 | NFE2L1 | STRADB | B3GALT4 | FXYD1 | NF1 | SORBS2 |
| BMPER | GBX1 | NFKBIZ | STT3B | B3GAT2 | FXYD5 | NF2 | SORD |
| BMPR1A | GCLM | NFU1 | STX17 | B3GNT5 | FZD8 | NFKBIB | SORT1 |
| BNIP3 | GCOM1 | NFXL1 | STXBP1 | B3GNT9 | G6PC | NFKBID | SOX9 |
| BOD1 | GCSH | NGDN | STXBP3 | B4GALT1 | GAB2 | NFKBIE | SPAG4 |
| BOLA1 | GDPD1 | NGF | STXBP6 | B9D1 | GABARAPL1 | NFX1 | SPAG5 |
| BOLA3 | GEMIN4 | NGFRAP1 | SUCLA2 | BACE2 | GABRA3 | NGFR | SPARC |
| BRD1 | GFRA1 | NHEJ1 | SUMF2 | BAMBI | GABRD | NGRN | SPATA2L |
| BRD7P3 | GFRA2 | NHLRC2 | SUMO2 | BAX | GALNT10 | NHEDC2 | SPC24 |
| BRMS1L | GGCT | NIF3L1 | SUSD4 | BBC3 | GALNT5 | NHSL1 | SPC25 |
| BRUNOL6 | GGTA1 | NINL | SUV420H1 | BBS2 | GARNL3 | NINJ1 | SPEG |
| BRWD2 | GHR | NIPSP3B | SYCP3 | BCAS4 | GARS | NINJ2 | SPIC |
| BTAF1 | GIMAP1 | NKAPL | SYF2 | BCL2 | GAS7 | NIPA1 | SPIN4 |
| BTBD3 | GIMAP2 | NKG7 | SYNPR | BCL2L14 | GATAD2A | NIPAL3 | SPINK1 |
| BTBD6 | GIMAP4 | NKIRAS1 | SYP | BCL6B | GBA3 | NIPSP1 | SPINK5 |
| BTD | GIMAP5 | NLRC5 | TADA2B | BCORL2 | GCET2 | NKIRAS2 | SPINT2 |
| BTF3 | GIMAP6 | NOC3L | TAF11 | BECN1 | GDAP1 | NKX2-3 | SPIRE1 |
| BTF3L4 | GIMAP7 | NOG | TAF5 | BEST1 | GDF11 | NLK | SPIRE2 |
| BTN3A1 | GIMAP8 | NOL11 | TAF6L | BEX1 | GDF15 | NLRC3 | SPOCD1 |
| BTN3A2 | GIN1 | NOL12 | TARBP1 | BEX4 | GDF3 | NLRP14 | SPON1 |
| BTN3A3 | GINS3 | NPEPPS | TATDN1 | BFSP1 | GDI2 | NME1 | SPP1 |
| BZRAP1 | GIPC2 | NR1D2 | TBC1D15 | BGN | GDNF | NMT1 | SPRR1A |
| BZW2 | GJA1 | NR2C2 | TBC1D4 | BICD1 | GDPD5 | NMT2 | SPRY4 |
| C10orf10 | GJA3 | NR3C2 | TBX18 | BIRC5 | GEM | NOP16 | SPRYD3 |
| C10orf104 | GJA5 | NRF1 | TBX21 | BLMH | GEMIN6 | NOP56 | SPTAN1 |
| C10orf110 | GJB2 | NRIP1 | TBX5 | BLOC1S3 | GEMIN8 | NOS3 | SPTB |
| C10orf116 | GK5 | NRN1 | TC2N | BMP6 | GGH | NOSTRIN | SPTBN1 |
| C10orf128 | GLDN | NRXN1 | TCEA3 | BMP8A | GINS2 | NOTCH2NL | SPTLC2 |
| C10orf137 | GLRX2 | NSDHL | TCEAL1 | BMPR2 | GIPC3 | NOVA1 | SRA1 |
| C10orf58 | GLRX5 | NTHL1 | TCEAL2 | BOK | GIT1 | NOX4 | SRD5A1 |
| C10orf72 | G13 | NUDT13 | TCEB1 | BOP1 | GKN1 | NP | SRF |
| C10orf76 | GO1 | NUDT19 | TCEB3 | BPESC1 | GLB1L | NPC2 | SRM |
| C11orf10 | GNG5 | NUDT3 | TCHP | BRAF | GLDC | NPHP1 | SRP19 |
| C11orf30 | GNLY | NUDT4 | TCP10L | BRD4 | GLG1 | NPHS2 | SRPK2 |
| C11orf45 | GNMT | NUDT6 | TDRD12 | BRD9 | GLIS2 | NPLOC4 | SRPK3 |
| C11orf46 | GNPAT | NUDT7 | TDRD3 | BRE | GLRB | NPPA | SRPR |
| C11orf53 | GOLGA8A | NUMA1 | TDRD6 | BRIP1 | GLS | NPPB | SRPRB |
| C11orf58 | GOLGA8B | NUMB | TDRD9 | BSPRY | GLT25D1 | NPR3 | SRPX2 |
| C11orf61 | GOLPH3L | NUP107 | TESC | BTBD10 | GLT8D2 | NR2E1 | SRR |
| C11orf67 | GOLSYN | NUP35 | TESK2 | BTN2A3 | GLT8D4 | NR4A1 | SRrp35 |
| C11orf71 | GOT2 | NXPH3 | TET1 | BUB1 | GLTP | NR4A3 | SS18 |
| C11orf73 | GPAM | OBSL1 | TFAM | BUB1B | GLUD2 | NR6A1 | SS18L2 |
| C11orf75 | GPATCH3 | OCEL1 | TFB1M | BUD31 | GM2A | NRARP | SSBP4 |
| C11orf87 | GPBP1 | OCIAD1 | TFEB | BVES | G14 | NRIP3 | SSH2 |
| C12orf26 | GPD2 | OCRL | TFIP11 | C10orf114 | GI2 | NRK | SSPN |
| C12orf35 | GPIHBP1 | ODZ2 | TGDS | C10orf12 | GNB1 | NRP1 | SSR3 |
| C12orf39 | GPLD1 | OLFM4 | TGFBR3 | C10orf25 | GNB3 | NRP2 | SSR4 |
| C12orf66 | GPM6B | OLFML1 | TH1L | C10orf26 | GNB5 | NRSN1 | SSSCA1 |
| C13orf18 | GPN3 | OMA1 | THAP11 | C10orf27 | GNG12 | NSF | SSX2IP |
| C13orf30 | GPR116 | OPCML | THNSL1 | C10orf35 | GNG3 | NSUN4 | ST3GAL2 |
| C13orf36 | GPR124 | OPN3 | THOC7 | C10orf40 | GNG8 | NSUN6 | ST3GAL4 |
| C14orf109 | GPR133 | ORC3L | THRA | C10orf68 | GNL3L | NT5C2 | ST3GAL6 |
| C14orf139 | GPR137B | ORC4L | THSD7A | C10orf75 | GNS | NUAK1 | ST6GALC4 |
| C14orf159 | GPR143 | OSGEPL1 | THUMPD1 | C10orf93 | GOLGA3 | NUDCD3 | ST7L |
| C14orf2 | GPR146 | OSR1 | THUMPD2 | C11orf17 | GOLIM4 | NUDT14 | ST8SIA2 |
| C14orf28 | GPR180 | OSR2 | TICAM2 | C11orf24 | GOLM1 | NUDT16 | ST8SIA5 |
| C14orf43 | GPR22 | OXA1L | TIFA | C11orf41 | GOSR2 | NUDT22 | STAC3 |
| C14orf48 | GPR25 | OXSM | TIGD1 | C11orf49 | GP1BA | NUDT5 | STAG3 |
| C14orf64 | GPR34 | P2RY1 | TIMP3 | C11orf51 | GP5 | NUF2 | STAG3L4 |
| C14orf73 | GPR35 | P2RY13 | TINF2 | C11orf63 | GPA33 | NUP188 | STARD7 |
| C15orf17 | GPR83 | P2RY14 | TINP1 | C11orf76 | GPC1 | NUP62CL | STAT3 |
| C15orf38 | GPRC5B | P2RY2 | TJP2 | C11orf80 | GPN2 | NUSAP1 | STGC3 |
| C15orf56 | GRAMD1C | PABPC4 | TKT | C11orf82 | GPR101 | NUTF2 | STIL |
| C15orf59 | GRB14 | PAIP1 | TKTL1 | C12orf24 | GPR137C | NXF5 | STIP1 |
| C15orf61 | GREB1 | PAIP2B | TLE1 | C12orf32 | GPR15 | NXNL1 | STK16 |
| C16orf52 | GRIN2A | PAN2 | TLR3 | C12orf4 | GPR173 | NXPH4 | STK17A |
| C16orf54 | GRIN3A | PAN3 | TLR5 | C12orf44 | GPR3 | OBFC1 | STK17B |
| C17orf100 | GSDMC | PAPLN | TM2D1 | C12orf45 | GPR32 | OCLN | STK38L |
| C17orf44 | GSTA4 | PARD6B | TM2D3 | C12orf48 | GPR37 | ODC1 | STK39 |
| C17orf86 | GSTM1 | PARD6G | TMC8 | C12orf49 | GPR39 | ODZ4 | STK40 |
| C17orf95 | GSTM2 | PARP4 | TMCC1 | C12orf5 | GPR4 | OGDHL | STMN1 |
| C18orf18 | GSTM4 | PARVB | TMCO6 | C13orf31 | GPR50 | OGT | STS |
| C18orf55 | GSTM5 | PASK | TMEFF2 | C13orf38 | GPR63 | OIT3 | STT3A |
| C19orf12 | GTF2F2 | PATZ1 | TMEM120B | C14orf118 | GPR68 | OLFM3 | STX1B |
| C1orf103 | GTF2IRD2 | PAXIP1 | TMEM123 | C14orf119 | GPR84 | OLFML2A | STX2 |
| C1orf104 | GTF3C5 | PBX1 | TMEM126A | C14orf128 | GPR85 | OLR1 | STX3 |
| C1orf105 | GTF3C6 | PCDHB13 | TMEM128 | C14orf129 | GPX3 | OMG | STXBP5L |
| C1orf109 | GTPBP8 | PCDHB14 | TMEM131 | C14orf132 | GPX7 | ONECUT2 | STYXL1 |
| C1orf110 | GUCY1A3 | PCDHB15 | TMEM132B | C14orf133 | GPX8 | OPA1 | SULT1A1 |
| C1orf116 | GUCY2D | PCDHB16 | TMEM133 | C14orf135 | GRB2 | OPN1SW | SUPT3H |
| C1orf151 | GVIN1 | PCDHB4 | TMEM14B | C14orf169 | GREM1 | OPTN | SVEP1 |
| C1orf168 | GYS2 | PCDHB7 | TMEM161B | C14orf174 | GRHL1 | OR13C4 | SVIL |
| C1orf183 | GZMA | PCDHGA1 | TMEM168 | C14orf179 | GRIA4 | OR4C1P | SVOP |
| C1orf186 | GZMB | PCF11 | TMEM170B | C14orf182 | GRIK2 | OR51B5 | SWAP70 |
| C1orf204 | GZMH | PCID2 | TMEM177 | C14orf37 | GRIN2D | OR51I1 | SYDE1 |
| C1orf226 | H1F0 | PCIF1 | TMEM182 | C14orf50 | GRK5 | OR5L2 | SYN2 |
| C1orf52 | H2AFJ | PCK2 | TMEM200B | C15orf37 | GRLF1 | OR5P2 | SYNCRIP |
| C1orf66 | H2AFZ | PCMT1 | TMEM220 | C15orf49 | GRM8 | OR5V1 | SYNGAP1 |
| C1orf9 | H3F3B | PCMTD1 | TMEM27 | C16orf55 | GRN | OR6B1 | SYNJ1 |
| C1orf93 | H6PD | PCMTD2 | TMEM37 | C16orf57 | GSDMB | OR7C1 | SYNJ2 |
| C1orf95 | HACE1 | PCOLCE2 | TMEM38A | C16orf75 | GSTCD | ORAI2 | SYNPO |
| C1QBP | HACL1 | PCP4L1 | TMEM41B | C16orf89 | GSTO1 | ORC1L | SYNPO2L |
| C1RL | HADH | PCYOX1 | TMEM55A | C17orf103 | GSTO2 | ORMDL2 | SYNRG |
| C1S | HADHA | PDCD4 | TMEM55B | C17orf37 | GSTZ1 | ORMDL3 | SYT11 |
| C20orf166 | HADHB | PDCD5 | TMEM57 | C17orf50 | GTF2E1 | OSBPL10 | SYT12 |
| C20orf177 | HAND1 | PDE1C | TMEM65 | C17orf63 | GTF2E2 | OSBPL8 | SYT4 |
| C20orf19 | HAUS3 | PDE2A | TMEM67 | C17orf85 | GTPBP1 | OSGIN1 | SYTL2 |
| C20orf197 | HAUS4 | PDE7B | TMEM68 | C17orf96 | GTSE1 | OSGIN2 | SYTL4 |
| C20orf24 | HCFC1R1 | PDGFRL | TMEM69 | C18orf2 | GUCA1C | OSTalpha | SYTL5 |
| C20orf7 | HCG11 | PDHB | TMEM70 | C18orf8 | GUK1 | OTUB2 | TACC2 |
| C21orf119 | HCG27 | PDIA6 | TMEM80 | C19orf28 | GUSB | OTUD1 | TACC3 |
| C21orf29 | HCG4 | PDIK1L | TMEM85 | C19orf33 | GYG1 | OVOL2 | TAF5L |
| C21orf34 | hCG_1646157 | PDK2 | TMEM87A | C19orf42 | GYS1 | OXCT1 | TAF7L |
| C21orf66 | hCG_1820661 | PDP1 | TMOD1 | C19orf51 | GZF1 | OXGR1 | TAF8 |
| C22:CTA-250D10.9 | hCG_2008140 | PDZRN3 | TMTC1 | C19orf55 | H19 | OXD1 | TAL1 |
| C22orf29 | hCG_2024094 | PEBP4 | TNFSF10 | C19orf63 | HAAO | P2RX1 | TANC2 |
| C22orf32 | hCG_2039027 | PECR | TNFSF12 | C1orf114 | HABP2 | P2RX2 | TAS2R4 |
| C22orf39 | hCG_2039148 | PET112L | TNK1 | C1orf122 | HAPLN1 | P2RX4 | TAX1BP3 |
| C2orf28 | HCN4 | PEX1 | TNMD | C1orf135 | HAPLN3 | P2RX5 | TBC1D20 |
| C2orf40 | HCP5 | PEX11A | TNNI3K | C1orf173 | HAPLN4 | P4HA1 | TBC1D22A |
| C2orf47 | HDAC1 | PEX13 | TOB2 | C1orf182 | HAVCR1 | P4HA2 | TBC1D22B |
| C2orf58 | HDAC3 | PEX19 | TOP2B | C1orf190 | HBEGF | P4HA3 | TBCD |
| C2orf64 | HDAC4 | PEX3 | TOX | C1orf194 | HCCS | P4HTM | TBL1XR1 |
| C2orf68 | HDGF | PEX7 | TP53TG1 | C1orf198 | hCG_1806964 | PABPC1L | TBXA2R |
| C2orf74 | HEATR2 | PFKFB3 | TPCN2 | C1orf21 | hCG_1817306 | PACRG | TCP11L1 |
| C3 | HEBP1 | PFKM | TPPP | C1orf230 | hCG_1818231 | PACS1 | TCTA |
| C3orf17 | HELQ | PFN2 | TPRG1 | C1orf46 | hCG_2007354 | PACSIN1 | TCTEX1D1 |
| C3orf26 | HERPUD1 | PGAM2 | TPRKB | C1orf54 | hCG_2011852 | PADI1 | TCTN3 |
| C3orf31 | HERPUD2 | PGM1 | TPST2 | C1orf64 | hCG_2045206 | PAFAH1B1 | TDG |
| C3orf43 | HEXIM2 | PGM5P1 | TPT1 | C1orf69 | HDAC8 | PAFAH1B2 | TDP1 |
| C3orf45 | HEY2 | PGR | TRDN | C1orf77 | HDAC9 | PAFAH1B3 | TEAD3 |
| C3orf55 | HFM1 | PHACTR3 | TRIAP1 | C1orf86 | HDGFL1 | PAK1 | TEAD4 |
| C4orf26 | HIBCH | PHB2 | TRIM16 | C1orf91 | HDHD2 | PALMD | TEC |
| C4orf3 | HIF3A | PHF10 | TRIM22 | C1orf97 | HEATR7A | PAMR1 | TECPR2 |
| C4orf32 | HINT1 | PHF11 | TRIM23 | C1QTNF6 | HELLS | PANK3 | TES |
| C4orf34 | HIPK3 | PHF12 | TRIM45 | C20orf103 | HERC5 | PANX1 | TEX12 |
| C4orf39 | HISPPD1 | PHF16 | TRIM52 | C20orf117 | HEXB | PAQR4 | TEX13A |
| C4orf43 | HIST1H1C | PHF17 | TRIM63 | C20orf194 | HEYL | PAQR6 | TFAP2D |
| C5orf13 | HIST1H2AI | PHF2 | TRIM7 | C20orf26 | HIF1AN | PAQR8 | TFDP2 |
| C5orf26 | HIST1H2BH | PHF3 | TRIM73 | C20orf3 | HIP1R | PARVA | TFPT |
| C5orf28 | HIST1H3H | PHGDH | TRIP12 | C20orf54 | HIPK2 | PBK | TGFB1 |
| C5orf33 | HIST3H2A | PHKB | TRIT1 | C20orf94 | HISPPD2A | PBLD | TGFB2 |
| C5orf4 | HIVEP3 | PHKG1 | TRMT61B | C21orf105 | HIST1H1B | PBOV1 | TGFB3 |
| C5orf41 | HLA-C | PHYH | TRPM7 | C21orf67 | HIST1H2AM | PBXIP1 | TGFBI |
| C5orf54 | HLA-DPA1 | PHYHIP | TSC1 | C21orf7 | HIST1H2BJ | PC | TGM2 |
| C5orf55 | HLA-DQB2 | PID1 | TSEN15 | C21orf91 | HIST1H3C | PCBP3 | TGOLN2 |
| C5orf56 | HLA-F | PIGG | TSEN2 | C21orf93 | HIST1H3J | PCBP4 | THBS1 |
| C6orf112 | HLA-G | PIGH | TSHZ1 | C22orf23 | HIST3H3 | PCDH11X | THBS2 |
| C6orf136 | HLF | PIGM | TSKU | C22orf25 | HIVEP2 | PCDH12 | THBS4 |
| C6orf141 | HMGB1 | PIGY | TSX | C22orf41 | HJURP | PCDH20 | THOC6 |
| C6orf192 | HMGCLL1 | PIK3AP1 | TSPAN15 | C2CD2 | HK2 | PCGF2 | THY1 |
| C6orf203 | HMGCS2 | PIK3IP1 | TSPAN7 | C2orf29 | HLCS | PCLO | TIAM2 |
| C6orf204 | HMGN3 | PIK3R1 | TST | C2orf52 | HMGA2 | PCNT | TIMM22 |
| C6orf211 | HNRNPA1 | PIM3 | TTC1 | C2orf55 | HMGB3 | PCYT1A | TIMP1 |
| C6orf70 | HNRNPA2B1 | PIN4 | TTC16 | C2orf63 | HMGB3L1 | PDCD11 | TK1 |
| C7orf10 | HNRNPD | PION | TTC17 | C2orf7 | HMHB1 | PDCD2 | TK2 |
| C7orf13 | HNRNPK | PIP5K1B | TTC18 | C2orf70 | HMMR | PDE10A | TKTL2 |
| C7orf25 | HNRNPM | PIR | TTC31 | C2orf73 | HMP19 | PDE1A | TLCD1 |
| C7orf30 | HNRNPR | PITPNM2 | TTC32 | C3orf14 | HN1 | PDE1B | TLL1 |
| C7orf70 | HNRNPU | PITPNM3 | TTC33 | C3orf21 | HN1L | PDE3B | TM4SF18 |
| C8orf12 | HNRNPUL1 | PKD1L1 | TTC35 | C3orf30 | HNF4G | PDE4B | TM4SF5 |
| C8orf34 | HNRPDL | PKIA | TTC7B | C3orf57 | HOMER3 | PDE4D | TM6SF2 |
| C8orf37 | HNRPLL | PKNOX2 | TTYH2 | C3orf75 | HOOK2 | PDE5A | TMCC2 |
| C8orf40 | HOMER2 | PKP2 | TUBA3D | C4orf48 | HOPX | PDE6A | TMCO3 |
| C8orf45 | HOXB2 | PLA2G12A | TUBA8 | C5AR1 | HOXA7 | PDE6B | TMED3 |
| C8orf50 | HOXB3 | PLA2G4F | TUBD1 | C5orf23 | HPD | PDE6C | TMED9 |
| C8orf59 | HPCAL4 | PLA2G6 | TUBE1 | C5orf32 | HPRT1 | PDE8B | TMEM107 |
| C8orf77 | HPGDS | PLAC8 | TUSC1 | C5orf46 | HPS1 | PDGFB | TMEM117 |
| C8orf79 | HPN | PLAC8L1 | TUSC4 | C5orf47 | HPS5 | PDIA3 | TMEM119 |
| C9orf123 | HPR | PLAG1 | TXLNB | C5orf51 | HRK | PDIA4 | TMEM120A |
| C9orf46 | HSCB | PLCB4 | TXN | C6orf1 | HS6ST3 | PDK3 | TMEM125 |
| C9orf47 | HSD11B1 | PLCD3 | TXNDC15 | C6orf108 | HSBP1 | PDLIM1 | TMEM127 |
| C9orf5 | HSD17B11 | PLCL1 | TXNDC16 | C6orf115 | HSD17B14 | PDLIM3 | TMEM135 |
| C9orf64 | HSD17B4 | PLCL2 | TXNL1 | C6orf145 | HSD3B1 | PDLIM4 | TMEM136 |
| C9orf95 | HSDL2 | PLCXD3 | UAP1 | C6orf153 | HSP90B1 | PDLIM5 | TMEM165 |
| CA11 | HSF4 | PLD6 | UBA5 | C6orf173 | HSPA2 | PDLIM7 | TMEM170A |
| CA14 | HSPB11 | PLEK | UBAP1 | C6orf176 | HSPA4 | PDPN | TMEM176A |
| CA4 | HSPB2 | PLEKHA5 | UBAP2 | C6orf94 | HSPA4L | PDS5A | TMEM176B |
| CA5BP | HSPB3 | PLEKHF1 | UBE2A | C7orf41 | HSPA8 | PDXP | TMEM190 |
| CA7 | HSPBAP1 | PLEKHH1 | UBE2B | C7orf42 | HSPB1 | PDYN | TMEM217 |
| CAB39L | HTRA3 | PLEKHM3 | UBE2D1 | C7orf50 | HSPB6 | PDZD11 | TMEM43 |
| CAC1D | ICAM4 | PLGLB2 | UBE2D4 | C7orf53 | HSPC159 | PDZD8 | TMEM45A |
| CAC1G | ICK | PLN | UBE2E1 | C7orf58 | HSPH1 | PECAM1 | TMEM49 |
| CAC2D3 | IDE | PLTP | UBE2E3 | C7orf60 | HTR1E | PEF1 | TMEM51 |
| CAC2D4 | IDH1 | PM20D1 | UBE2I | C8orf41 | HTRA1 | PENK | TMEM63B |
| CACNB2 | IDH3B | PMP22 | UBE2K | C8orf49 | HUWE1 | PER4 | TMEM64 |
| CACYBP | IER3IP1 | PMPCB | UBE2R2 | C8orf51 | HYAL4 | PEX10 | TMEM71 |
| CADPS | IFIT1 | PNMA6A | UBE2U | C8orf58 | HYLS1 | PEX11G | TMEM86A |
| CALY | IFIT3 | PNPLA4 | UBE2V2 | C9orf100 | ICMT | PEX16 | TMEM8A |
| CAPN14 | IFIT5 | POL3S | UBL3 | C9orf102 | IDH3A | PEX26 | TMEM90B |
| CAPZA2 | IF6 | POLB | UBR3 | C9orf11 | IER2 | PFKFB2 | TMEM9B |
| CARD18 | IFNG | POLG2 | UBR5 | C9orf125 | IER3 | PFKL | TMPRSS11D |
| CAV3 | IFRD2 | POLI | UBXN8 | C9orf144 | IFI27L2 | PFN1 | TMSB10 |
| CBFA2T3 | IFT172 | POLM | UCHL5 | C9orf16 | IF8 | PFTK1 | TNC |
| CBLB | IFT74 | POLR1B | UCKL1 | C9orf25 | IFR2 | PGAM1 | TNFAIP1 |
| CBLL1 | IFT81 | POLR2I | UCP3 | C9orf3 | IFNGR2 | PGBD4 | TNFRSF11B |
| CBR4 | IGDCC4 | POLR3B | UGT2B4 | C9orf30 | IFRD1 | PGBD5 | TNFRSF12A |
| CBX1 | IGFBP5 | POLR3G | UHRF2 | C9orf33 | IGF1 | PGLS | TNFRSF25 |
| CBX7 | IGJ | POMP | ULK3 | C9orf40 | IGF1R | PGLYRP3 | TNNI3 |
| CCBE1 | IGSF1 | PON3 | UNC50 | C9orf62 | IGFBP2 | PGM5 | TNNT1 |
| CCDC101 | IGSF10 | POT1 | UNC5CL | C9orf68 | IGH@ | PHF19 | TNNT3 |
| CCDC104 | IGSF11 | POTEK | UNK | C9orf9 | IGSF22 | PHF20L1 | TNPO1 |
| CCDC111 | IKZF1 | POU2AF1 | UNKL | C9orf91 | IGSF5 | PHF21B | TNPO2 |
| CCDC121 | IKZF5 | POU6F1 | UNQ565 | C9orf93 | IKBKE | PHKA1 | TNRC4 |
| CCDC138 | IL15 | PPAP2B | UPB1 | CABP1 | IL11 | PHLDA3 | TOMM22 |
| CCDC142 | IL15RA | PPAPDC3 | UPF2 | CABYR | IL13 | PHLDB1 | TOMM40 |
| CCDC146 | IL17RB | PPARD | UPF3A | CAC1B | IL13RA1 | PHTF1 | TOMM70A |
| CCDC17 | IL17RD | PPARGC1B | UPF3B | CACNB1 | IL17C | PHTF2 | TOP1 |
| CCDC21 | IL18RAP | PPIL1 | UQCRB | CACNB4 | IL17D | PI4K2A | TOP2A |
| CCDC28B | IL1RAPL1 | PPIL3 | UQCRFS1 | CALD1 | IL1RL1 | PIAS2 | TOR1A |
| CCDC45 | IL20RA | PPP1CB | USP21 | CALM3 | IL23R | PIGK | TOX4 |
| CCDC51 | IL2RB | PPP1CC | USP24 | CALU | IL27 | PIK3C3 | TP53BP1 |
| CCDC59 | IL2RG | PPP1R13L | USP45 | CAMK2N2 | IL28A | PIK3CG | TP53INP2 |
| CCDC68 | IL6R | PPP1R1A | USP48 | CAMSAP1L1 | IL32 | PIK3R2 | TP63 |
| CCDC72 | IMP3 | PPP1R3B | VAMP1 | CANX | IL3RA | PIK3R3 | TPD52L3 |
| CCDC76 | IMPA2 | PPP1R3D | VASH2 | CAP1 | IL6 | PIK3R6 | TPH2 |
| CCDC84 | INCENP | PPP1R3E | VBP1 | CAP2 | ILK | PIM1 | TPM3 |
| CCDC93 | IP6K3 | PPP2R3A | VDAC2 | CAPN6 | IMPACT | PIP5K1C | TPM4 |
| CCL5 | IPO7 | PPP3CB | VEZF1 | CAPN8 | IMPAD1 | PITP | TPRXL |
| CCNG1 | IPW | PPP6C | VIP | CAPNS1 | INE1 | PKD2L2 | TPX2 |
| CCNJ | IRAK2 | PQLC3 | VIT | CAPRIN2 | INHA | PKMYT1 | TRA@ |
| CCNT2 | IRF2 | PRDM10 | VPRBP | CARD6 | INHBE | PKNOX1 | TRAF3 |
| CD244 | IRF2BP1 | PRDX2 | VPS13B | CARHSP1 | INMT | PLA2G7 | TRAF4 |
| CD247 | IRF2BP2 | PRDX3 | VPS24 | CARS | INPP4B | PLAGL2 | TRAF6 |
| CD300A | IRS1 | PRDX5 | VPS26A | CARTPT | INPP5A | PLAUR | TRAPPC1 |
| CD300LG | ISCA1 | PRELID2 | VPS54 | CASC2 | INPP5B | PLCD4 | TRAPPC9 |
| CD302 | ISOC1 | PRF1 | VTCN1 | CASC5 | INPP5F | PLCE1 | TRBV7-8 |
| CD36 | ITGAL | PRIM1 | VWA3A | CASP3 | INSM2 | PLCG2 | TRIB1 |
| CD5L | ITGB6 | PRKRA | WAS | CASP9 | INSR | PLEKHA3 | TRIM26 |
| CD69 | ITGB7 | PRKXP1 | WDR33 | CASQ1 | INTS3 | PLEKHB2 | TRIM35 |
| CD8A | ITK | PRMT3 | WDR51B | CASQ2 | INTS4L1 | PLEKHG1 | TRIM36 |
| CD96 | ITM2B | PRNP | WDR62 | CBFB | INTS7 | PLEKHG2 | TRIM37 |
| CDC16 | ITPKC | PRODH | WDR64 | CBL | IPO5 | PLIN3 | TRIM41 |
| CDC2L6 | JAM2 | PROK2 | WDR75 | CBLN4 | IPO9 | PLK3 | TRIM44 |
| CDC73 | JARID2 | PRPF18 | WDR91 | CBS | IPPK | PLK4 | TRIM59 |
| CDH13 | JKAMP | PRPF3 | WDSUB1 | CBY1 | IQCA1 | PLOD3 | TRIM6 |
| CDH26 | JUP | PRPF40A | WHAMML2 | CBY3 | IQCD | PLVAP | TRIM72 |
| CDIPT | KAT2B | PRPH | WHSC2 | CCDC102B | IQCF1 | PLXDC2 | TRIML1 |
| CDK5R1 | KBTBD6 | PRPH2 | WIBG | CCDC108 | IQGAP3 | PLX1 | TRIMP1 |
| CDKN1B | KBTBD7 | PRR12 | WISP3 | CCDC110 | IQSEC2 | PLX2 | TRIP13 |
| CDKN2AIP | KCMF1 | PRRG1 | WNK2 | CCDC113 | IQUB | PLXNB3 | TRIP4 |
| CDKN2C | KC2 | PRTFDC1 | WNT5A | CCDC124 | IRAK4 | PMCH | TRMT6 |
| CDNF | KC6 | PS1TP4 | WSCD2 | CCDC125 | IRGQ | PMCHL1 | TRO |
| CDON | KCND3 | PSMA3 | WWOX | CCDC144A | ISCA2 | PMEPA1 | TROAP |
| CECR5 | KCNH2 | PSMA4 | WWP1 | CCDC150 | ISG15 | PMS2L2 | TRPM1 |
| CECR6 | KCNH8 | PSMA8 | XCL1 | CCDC18 | ISG20L2 | PMS2L5 | TRPM4 |
| CEL | KCNIP2 | PSMB1 | XIST | CCDC25 | ITGA5 | PNCK | TRPV5 |
| CELSR2 | KCNJ2 | PSMB6 | XPOT | CCDC55 | ITGA7 | PNKD | TRUB1 |
| CENPV | KCNK1 | PSMB9 | XRCC6BP1 | CCDC6 | ITGAE | PNLIPRP2 | TSC22D2 |
| CEP192 | KCNK17 | PSMC3IP | XYLT1 | CCDC63 | ITGB3 | PNMA1 | TSGA10 |
| CFD | KCNMB2 | PSMD6 | YBX1 | CCDC75 | ITGB3BP | PNPLA1 | TSGA14 |
| CFLAR | KCNN2 | PSMD7 | YEATS4 | CCDC80 | ITM2C | PNPLA3 | TSXIP1 |
| CG012 | KCTD3 | PSME1 | YPEL3 | CCDC83 | IVNS1ABP | PNPO | TSPAN12 |
| CG030 | KDM6A | PSMG2 | YTHDC2 | CCDC85B | JAK2 | POLD4 | TSPAN17 |
| CHAD | KDSR | PTBP2 | YWHAG | CCDC87 | JAM3 | POLE2 | TSPAN18 |
| CHADL | KGFLP2 | PTCD2 | ZADH2 | CCDC96 | JAZF1 | POLH | TSPAN5 |
| CHCHD10 | KHK | PTCH1 | ZAP70 | CCIN | JMJD4 | POLQ | TSPAN9 |
| CHCHD3 | KIAA0114 | PTDSS1 | ZBED1 | CCL11 | JPH4 | POLR1E | TSR1 |
| CHCHD4 | KIAA0146 | PTER | ZBED5 | CCL25 | JUNB | PORCN | TSTA3 |
| CHD2 | KIAA0196 | PTGDR | ZBTB11 | CC1 | KALRN | POSTN | TTC26 |
| CHDH | KIAA0323 | PTGDS | ZBTB26 | CC2 | KBTBD8 | PPAPDC1A | TTC39A |
| CHI3L2 | KIAA0368 | PTGES | ZBTB40 | CCNB2 | KCNC4 | PPCS | TTC9 |
| CHMP5 | KIAA0406 | PTGES3 | ZBTB44 | CCND1 | KCNH7 | PPDPF | TTK |
| CHP | KIAA0528 | PTGR2 | ZC3H14 | CCNE1 | KCNIP1 | PPFIA3 | TTL |
| CHPT1 | KIAA0748 | PTP4A3 | ZC3H6 | CCNF | KCNIP4 | PPFIA4 | TTLL1 |
| CHRDL2 | KIAA0774 | PTPN13 | ZCCHC11 | CCNI | KCNJ14 | PPFIBP1 | TTLL11 |
| CHTF8 | KIAA0831 | PTPRC | ZCCHC8 | CCNK | KCNJ4 | PPIA | TTPAL |
| CITED4 | KIAA0892 | PTPRU | ZCWPW2 | CCT2 | KCNJ5 | PPIB | TTTY15 |
| CKM | KIAA0895 | PUM2 | ZDHHC11 | CCT3 | KCNJ8 | PPIL2 | TTYH3 |
| CKMT2 | KIAA0907 | PUS7L | ZDHHC17 | CCT5 | KCNK6 | PPM1E | TUBA1A |
| CLCN2 | KIAA0947 | PVRIG | ZDHHC6 | CCT6A | KCNMB1 | PPME1 | TUBA1B |
| CLCN4 | KIAA1012 | PWWP2A | ZFAND6 | CCT8 | KCNQ1 | PPP1R10 | TUBA1C |
| CLDN12 | KIAA1107 | PXDNL | ZFP112 | CCT8L2 | KCNQ4 | PPP1R12C | TUBA4A |
| CLDND1 | KIAA1143 | PXMP2 | ZFP2 | CD151 | KCTD10 | PPP1R14B | TUFT1 |
| CLEC1A | KIAA1217 | PYGM | ZFP30 | CD1A | KCTD11 | PPP1R7 | TWISTNB |
| CLEC2D | KIAA1267 | PYHIN1 | ZFP37 | CD274 | KCTD17 | PPP2CA | TWSG1 |
| CLGN | KIAA1466 | QKI | ZFP62 | CD44 | KCTD20 | PPP2R1B | TXNDC8 |
| CLK1 | KIAA1529 | QPRT | ZIK1 | CD46 | KCTD7 | PPP2R5A | TXNRD1 |
| CLK4 | KIAA1908 | QRICH2 | ZMYM5 | CD55 | KDELC1 | PPP2R5C | TYMS |
| CLPX | KIF22 | QRSL1 | ZNF133 | CD59 | KDELR3 | PPRC1 | TYW3 |
| CLYBL | KIF5C | QSOX2 | ZNF135 | CD63 | KDM2A | PPYR1 | TUBA1A |
| CMBL | KIR2DL2 | RAB11FIP2 | ZNF138 | CD80 | KIAA0020 | PQLC2 | TUBA1B |
| CMC1 | KIR2DS5 | RAB11FIP4 | ZNF14 | CDC14B | KIAA0101 | PRAF2 | TUBA1C |
| CMPK2 | KIR3DL3 | RAB12 | ZNF140 | CDC2 | KIAA0174 | PRC1 | TUBA4A |
| CMTM5 | KLF10 | RAB25 | ZNF141 | CDC20 | KIAA0182 | PRCP | TUFT1 |
| CMTM8 | KLF13 | RAB33B | ZNF142 | CDC25A | KIAA0226 | PRELID1 | TWISTNB |
| CNBP | KLF9 | RAB37 | ZNF148 | CDC26 | KIAA0319 | PREP | TWSG1 |
| CNIH | KLHDC1 | RAB43 | ZNF160 | CDC27 | KIAA0427 | PRIM2 | TXNDC8 |
| CNOT4 | KLHDC2 | RAB5B | ZNF17 | CDC34 | KIAA0513 | PRKCA | TXNRD1 |
| COG5 | KLHDC3 | RABGGTB | ZNF177 | CDC42EP5 | KIAA0556 | PRKCZ | TYMS |
| COL15A1 | KLHDC9 | RAC2 | ZNF182 | CDC45L | KIAA0746 | PRKG1 | TYW3 |
| COL24A1 | KLHL15 | RAD9B | ZNF184 | CDC6 | KIAA0802 | PRMT1 | UBAC2 |
| COL27A1 | KLHL21 | RAF1 | ZNF187 | CDC7 | KIAA1024 | PRMT10 | UBAP2L |
| COL28A1 | KLHL24 | RALA | ZNF189 | CDCA2 | KIAA1199 | PRO0471 | UBASH3B |
| COL4A6 | KLHL26 | RALGDS | ZNF2 | CDCA3 | KIAA1211 | PROM1 | UBE2C |
| COL5A3 | KLHL32 | RALYL | ZNF204 | CDCA4 | KIAA1244 | PROS1 | UBE2DNL |
| COL9A3 | KLHL7 | RANBP2 | ZNF207 | CDCA5 | KIAA1279 | PRR10 | UBE2H |
| COLEC12 | KLHL8 | RAP2C | ZNF211 | CDCA7 | KIAA1539 | PRRG2 | UBE2L3 |
| COMMD3 | KLKB1 | RARRES1 | ZNF222 | CDCA8 | KIAA1644 | PRRT3 | UBE2Q1 |
| COPS2 | KLRB1 | RARRES3 | ZNF224 | CDH17 | KIAA1826 | PRSS22 | UBE2Q2 |
| COPS5 | KLRD1 | RASA1 | ZNF23 | CDH2 | KIAA1920 | PRSS23 | UBE2T |
| COPS6 | KLRF1 | RASD1 | ZNF232 | CDH8 | KIAA2013 | PRSS27 | UBE2W |
| COQ10A | KLRK1 | RASD2 | ZNF233 | CDK5 | KIF11 | PRSS35 | UBE2Z |
| COQ3 | KP5 | RASL10B | ZNF234 | CDK8 | KIF13A | PSD | UBE3C |
| CORIN | KPTN | RBBP7 | ZNF236 | CDKL1 | KIF14 | PSMA5 | UBQLN4 |
| CORO1A | KRCC1 | RBKS | ZNF238 | CDKN1A | KIF18A | PSMD8 | UBR1 |
| COX11 | KRIT1 | RBL2 | ZNF250 | CDKN2A | KIF1A | PSMG3 | UBR4 |
| COX17 | KRT10 | RBM16 | ZNF251 | CDKN2B | KIF1C | PSORS1C1 | UBXN2B |
| COX4I1 | KRT74 | RBM20 | ZNF253 | CDKN2D | KIF20A | PTGFRN | UBXN6 |
| COX5A | KRTAP10-11 | RBM24 | ZNF254 | CDKN3 | KIF21A | PTGR1 | UCHL3 |
| COX5B | KY | RBM28 | ZNF267 | CDR2L | KIF23 | PTK7 | UCK2 |
| COX6B1 | L3MBTL3 | RBM41 | ZNF271 | CDRT1 | KIF2C | PTP4A1 | UCMA |
| COX6C | LACTB2 | RBM4B | ZNF283 | CDV3 | KIF3B | PTPDC1 | UCN |
| COX7B | LAMB3 | RBMX | ZNF285A | CENPA | KIF4A | PTPN14 | UGCG |
| COX7C | LAMC2 | RBP3 | ZNF30 | CENPE | KIF9 | PTPN21 | UGGT1 |
| COX8C | LAPTM4A | RCBTB1 | ZNF302 | CENPF | KIFAP3 | PTPN4 | UHRF1 |
| CPA3 | LARP4B | RCHY1 | ZNF304 | CENPH | KIFC1 | PTPRA | ULBP2 |
| CPEB2 | LBR | RCOR3 | ZNF319 | CENPI | KLC2 | PTPRB | UNC13C |
| CPEB3 | LCK | RDH14 | ZNF32 | CENPJ | KLF14 | PTPRH | UNC45A |
| CPN1 | LCLAT1 | RECQL | ZNF320 | CENPM | KLF16 | PTPRR | UPP1 |
| CPNE4 | LCORL | REEP1 | ZNF322A | CENPN | KLF7 | PTRF | UQCRQ |
| CPO | LCP1 | REPIN1 | ZNF329 | CEP152 | KLHDC4 | PTTG1 | UROS |
| CPSF4 | LDB3 | RERG | ZNF333 | CEP55 | KLHDC5 | PUS1 | USP11 |
| CPSF6 | LGR4 | RET | ZNF337 | CEP70 | KLHL12 | PVRL2 | USP5 |
| CREB3L4 | LGR6 | REV1 | ZNF33A | CEP76 | KLHL13 | PVT1 | VAMP5 |
| CREBBP | LGTN | REV3L | ZNF33B | CERCAM | KLHL18 | PXDN | VAPB |
| CREBL2 | LHFP | RFC3 | ZNF346 | CERK | KLHL25 | PYGO1 | VASH1 |
| CREBZF | LIFR | RFESD | ZNF347 | CES1 | KLHL29 | QPCT | VASP |
| CRIPAK | LIN7C | RFXAP | ZNF350 | CES2 | KLHL34 | QSK | VAT1 |
| CRIPT | LINS1 | RG9MTD1 | ZNF354A | CES8 | KLHL36 | RAB11B | VCAN |
| CRLS1 | LIPT1 | RG9MTD2 | ZNF37B | CETP | KLKBL4 | RAB11FIP3 | VCL |
| CROCCL1 | LMBRD1 | RGL4 | ZNF382 | CFHR2 | KP1 | RAB11FIP5 | VDR |
| CRTAM | LOC100128071 | RGMA | ZNF383 | CHAC1 | KP2 | RAB13 | VGLL4 |
| CRYL1 | LOC100128288 | RGMB | ZNF385C | CHAF1A | KP6 | RAB15 | VHL |
| CS | LOC100128398 | RGN | ZNF404 | CHCHD5 | KPNB1 | RAB17 | VLDLR |
| CSAD | LOC100128640 | RGNEF | ZNF418 | CHD1L | KRT15 | RAB23 | VN1R1 |
| CSE1L | LOC100128822 | RGS6 | ZNF420 | CHD5 | KRT18P44 | RAB26 | VN1R3 |
| CSF1 | LOC100129195 | RGS9BP | ZNF432 | CHD7 | KRT24 | RAB28 | VOPP1 |
| CSGALCT1 | LOC100129282 | RHCE | ZNF441 | CHD8 | KRT4 | RAB31 | VPS18 |
| CSRP2BP | LOC100129550 | RHD | ZNF443 | CHEK1 | KRT80 | RAB32 | VPS37C |
| CST7 | LOC100129637 | RHOC | ZNF45 | CHEK2 | KRTAP1-5 | RAB3IP | VPS53 |
| CSTF2T | LOC100129895 | RHOT1 | ZNF471 | CHIC2 | KRTAP2-1 | RAB4A | VTI1B |
| CTAGE4 | LOC100130219 | RIC8B | ZNF485 | CHMP4B | KRTAP4-1 | RAB5C | VTN |
| CTAGE5 | LOC100130285 | RICS | ZNF491 | CHMP4C | KRTAP4-2 | RAB8A | WBSCR17 |
| CTCF | LOC100130837 | RINT1 | ZNF493 | CHPF2 | L1TD1 | RAB9B | WDHD1 |
| CTDSP2 | LOC100131354 | RIOK2 | ZNF502 | CHR1 | LACE1 | RABEP1 | WDR1 |
| CTNNBIP1 | LOC100131512 | RMI1 | ZNF511 | CHR3 | LAD1 | RABEPK | WDR51A |
| CTPS2 | LOC100131581 | RSE4 | ZNF514 | CHRNB1 | LAMA2 | RABGAP1 | WDR54 |
| CTR9 | LOC100133686 | RSEH2C | ZNF519 | CHST11 | LAMA3 | RABGEF1 | WDR60 |
| CTSL1 | LOC100134361 | RNF114 | ZNF521 | CHST15 | LAMA4 | RAD18 | WDR63 |
| CTSW | LOC100134937 | RNF13 | ZNF529 | CHST3 | LAMB1 | RAD51 | WDR66 |
| CUGBP2 | LOC100144604 | RNF149 | ZNF542 | CHST6 | LAMB2 | RAD51AP1 | WDR76 |
| CUL3 | LOC100190939 | RNF165 | ZNF543 | CHST7 | LAMP1 | RAD54L | WDR77 |
| CUL4A | LOC100216545 | RNF166 | ZNF548 | CIAPIN1 | LARP1 | RAI14 | WEE1 |
| CUL5 | LOC100270804 | RNF212 | ZNF555 | CIB2 | LARP4 | RALY | WFDC13 |
| CX3CR1 | LOC100272217 | RNF220 | ZNF559 | CIRH1A | LASS5 | RAMP1 | WHSC1 |
| CXCL12 | LOC120376 | RNF44 | ZNF566 | CISD1 | LATS2 | RANBP17 | WIPF3 |
| CXCL14 | LOC126661 | RNF5 | ZNF568 | CISD3 | LAX1 | RANBP9 | WIPI1 |
| CXorf24 | LOC134466 | RNF6 | ZNF569 | CKAP2 | LAYN | RAP1GDS1 | WISP1 |
| CXorf58 | LOC144571 | RNLS | ZNF571 | CKAP2L | LCAT | RAP2A | WNT16 |
| CXorf65 | LOC145820 | ROPN1 | ZNF573 | CKAP4 | LCN12 | RARA | WNT9A |
| CXXC1 | LOC146336 | RORC | ZNF577 | CKAP5 | LDHC | RARS2 | WWC1 |
| CXXC4 | LOC148189 | RP11-144G6.7 | ZNF585A | CKS2 | LDHD | RASIP1 | WWC3 |
| CXXC5 | LOC149134 | RP11-345P4.4 | ZNF594 | CLCA3P | LEF1 | RASSF2 | WWTR1 |
| CYB5A | LOC150622 | RP3-377H14.5 | ZNF599 | CLCN5 | LEPREL1 | RASSF8 | XIRP1 |
| CYB5D1 | LOC152217 | RPA2 | ZNF606 | CLEC12A | LEPREL2 | RBBP8 | XPO4 |
| CYBRD1 | LOC153346 | RPAIN | ZNF614 | CLEC16A | LEPROTL1 | RBBP9 | XPO5 |
| CYC1 | LOC157381 | RPAP2 | ZNF615 | CLEC4C | LGALS9 | RBM33 | XPR1 |
| CYP39A1 | LOC157562 | RPGRIP1L | ZNF616 | CLIC1 | LGI2 | RBM8A | XRCC4 |
| CYP4B1 | LOC171220 | RPL10 | ZNF623 | CLIC4 | LHB | RBM9 | YAF2 |
| CYP4X1 | LOC202051 | RPL10L | ZNF624 | CLIC5 | LHFPL2 | RBMS2 | YDJC |
| CYP4Z1 | LOC202781 | RPL13A | ZNF626 | CLN6 | LHFPL3 | RBMX2 | YIF1A |
| CYTH4 | LOC220930 | RPL15 | ZNF639 | CLN8 | LHFPL5 | RBMY3AP | YLPM1 |
| CYTIP | LOC221272 | RPL21 | ZNF671 | CLOCK | LHX6 | RBP1 | YPEL1 |
| CYC1 | LOC222070 | RPL23A | ZNF680 | CLSPN | LIMK1 | RBP7 | YPEL2 |
| CYP39A1 | LOC255512 | RPL27 | ZNF682 | CLSTN1 | LIN7B | RC3H2 | YWHAB |
| D4S234E | LOC283270 | RPL30 | ZNF688 | CLTCL1 | LIPC | RCAN1 | YWHAH |
| DAB1 | LOC283357 | RPL31 | ZNF691 | CLUAP1 | LIPE | RCC2 | YWHAQ |
| DAPK2 | LOC283392 | RPL32 | ZNF706 | CMIP | LLPH | RCVRN | UBAC2 |
| DARS | LOC283481 | RPL32P3 | ZNF708 | CMKLR1 | LMLN | RD3 | UBAP2L |
| DAZAP1 | LOC283547 | RPL37A | ZNF721 | CMYA5 | LMNB1 | RDBP | UBASH3B |
| DBI | LOC283588 | RPL38 | ZNF738 | CNDP2 | LMNB2 | RDH13 | UBE2C |
| DBR1 | LOC284276 | RPL39 | ZNF74 | CNGA1 | LMOD2 | RDM1 | UBE2DNL |
| DCAF16 | LOC284373 | RPL41 | ZNF75A | CNKSR2 | LMTK2 | REEP5 | UBE2H |
| DCI | LOC284513 | RPL7A | ZNF776 | CNKSR3 | LNP1 | REG3G | UBE2L3 |
| DCN | LOC284630 | RPLP0 | ZNF781 | CNN1 | LNPEP | RELL1 | UBE2Q1 |
| DCP2 | LOC285014 | RPP30 | ZNF782 | CNNM2 | LNX2 | RELN | UBE2Q2 |
| DCTPP1 | LOC285835 | RPP38 | ZNF784 | CNTD2 | LOC100126784 | RELT | UBE2T |
| DCUN1D5 | LOC285943 | RPRD2 | ZNF792 | COBL | LOC100127983 | RERE | UBE2W |
| DDHD2 | LOC285957 | RPS10 | ZNF816A | COL10A1 | LOC100128164 | REST | UBE2Z |
| DDX10 | LOC285972 | RPS10P5 | ZNF823 | COL14A1 | LOC100128496 | RFX4 | UBE3C |
| DDX46 | LOC285986 | RPS13 | ZNF828 | COL16A1 | LOC100128501 | RFX5 | UBQLN4 |
| DDX47 | LOC286052 | RPS16 | ZNF835 | COL18A1 | LOC100128511 | RFX7 | UBR1 |
| DDX60 | LOC286178 | RPS17 | ZNF836 | COL1A1 | LOC100128612 | RGAG1 | UBR4 |
| DECR1 | LOC286189 | RPS17P5 | ZNF837 | COL1A2 | LOC100128653 | RGL1 | UBXN2B |
| DEK | LOC339290 | RPS23 | ZNF839 | COL29A1 | LOC100128881 | RGS1 | UBXN6 |
| DENND1C | LOC340184 | RPS25 | ZNF84 | COL3A1 | LOC100128893 | RGS4 | UCHL3 |
| DENND2D | LOC340544 | RPS3A | ZNF846 | COL4A1 | LOC100128993 | RHBDD2 | UCK2 |
| DENND4B | LOC374443 | RPS4X | ZNF862 | COL4A2 | LOC100129058 | RHBDL2 | UCMA |
| DENND4C | LOC387647 | RPS5 | ZNF91 | COL4A3BP | LOC100129129 | RHCG | UCN |
| DERL3 | LOC388789 | RPS6KA5 | ZNRF3 | COL5A1 | LOC100129194 | RHOBTB2 | UGCG |
| DET1 | LOC389834 | RPS7 | ZSWIM7 | COL5A2 | LOC100129361 | RIBC2 | UGGT1 |
| DGAT2 | LOC390998 | RPS8 | ZW10 | COL6A3 | LOC100129716 | RIOK3 | UHRF1 |
| DHFRL1 | LOC400027 | RRAGA | ZXDC | COL8A1 | LOC100129858 | RSE2 | ULBP2 |
| DHODH | LOC400043 | RRAGD | ZZZ3 | COLEC11 | LOC100130155 | RSE7 | UNC13C |
| DHRS1 | LOC401321 | RRP15 |  | COLQ | LOC100130175 | RND3 | UNC45A |
| DHRS12 | LOC401324 | RSAD1 |  | COMMD1 | LOC100130502 | RNF10 | UPP1 |
| DHRS7C | LOC401397 | RSBN1L |  | COMMD8 | LOC100130938 | RNF115 | UQCRQ |
| DHX29 | LOC439911 | RSL24D1 |  | COMT | LOC100130964 | RNF122 | UROS |
| DIDO1 | LOC439949 | RSU1 |  | COPA | LOC100130998 | RNF128 | USP11 |
| DIO3OS | LOC440173 | RUNDC3B |  | COPB2 | LOC100131031 | RNF133 | USP5 |
| DIRAS3 | LOC440498 | RUNX2 |  | COPG | LOC100131262 | RNF148 | VAMP5 |
| DIRC2 | LOC440552 | RUNX3 |  | COPG2 | LOC100131283 | RNF150 | VAPB |
| DIRC3 | LOC440934 | RWDD1 |  | COPZ2 | LOC100131366 | RNF168 | VASH1 |
| DKFZP434I0714 | LOC440957 | RXRA |  | COQ10B | LOC100131480 | RNF20 | VASP |
| DKFZp779M0652 | LOC440993 | RXRG |  | COQ2 | LOC100131683 | RNF208 | VAT1 |
| DLAT | LOC441242 | RYR2 |  | CORO1C | LOC100131691 | RNF214 | VCAN |
| DLD | LOC442028 |  |  | COX15 | LOC100131781 | RNF216 | VCL |
| DLK1 | LOC51152 |  |  | COX6A1 | LOC100132167 | RNF217 | VDR |
| DLL1 | LOC550643 |  |  | CP | LOC100133287 | RNF24 | VGLL4 |
| DMGDH | LOC572558 |  |  | CPEB4 | LOC100133308 | RNF34 | VHL |
| DMTF1 | LOC641467 |  |  | CPN2 | LOC100133985 | RNF38 | VLDLR |
| DH3 | LOC642236 |  |  | CPPED1 | LOC100134713 | RNGTT | VN1R1 |
| DJA3 | LOC642776 |  |  | CPSF2 | LOC100144603 | RNH1 | VN1R3 |
| DJB9 | LOC643008 |  |  | CPT1C | LOC100216479 | RNMT | VOPP1 |
| DJC10 | LOC643072 |  |  | CPVL | LOC100270680 | ROBO4 | VPS18 |
| DJC19 | LOC643837 |  |  | CPXCR1 | LOC144481 | ROR1 | VPS37C |
| DJC27 | LOC644215 |  |  | CR2 | LOC145837 | RP1-21O18.1 | VPS53 |
| DNM3 | LOC644242 |  |  | CRB1 | LOC146513 | RP11-165H20.1 | VTI1B |
| DOC2B | LOC644538 |  |  | CRELD1 | LOC149832 | RP11-297H3.4 | VTN |
| DOCK8 | LOC644656 |  |  | CRIP3 | LOC151658 | RP11-529I10.4 | WBSCR17 |
| DPP6 | LOC645212 |  |  | CRISPLD2 | LOC151877 | RP11-756A22.3 | WDHD1 |
| DPY19L1 | LOC645513 |  |  | CRK | LOC152742 | RP5-1022P6.6 | WDR1 |
| DPY19L2 | LOC645676 |  |  | CRLF1 | LOC153328 | RP6-213H19.1 | WDR51A |
| DPY19L2P2 | LOC646903 |  |  | CRLF2 | LOC158960 | RPL13AP17 | WDR54 |
| DPY30 | LOC647979 |  |  | CRMP1 | LOC196415 | RPL23AP53 | WDR60 |
| DPYD | LOC727770 |  |  | CRYBB3 | LOC202451 | RPL26L1 | WDR63 |
| DPYSL2 | LOC728052 |  |  | CRYGC | LOC221122 | RPL37 | WDR66 |
| DPYSL4 | LOC728142 |  |  | CRYM | LOC253044 | RPL39L | WDR76 |
| DRAM2 | LOC728543 |  |  | CSNK1E | LOC253805 | RPN2 | WDR77 |
| DSCAML1 | LOC728705 |  |  | CSNK1G1 | LOC256021 | RPS12 | WEE1 |
| DSCR8 | LOC728769 |  |  | CSNK2A2 | LOC283028 | RPS19BP1 | WFDC13 |
| DSP | LOC729013 |  |  | CSPG4 | LOC283045 | RPS6KA2 | WHSC1 |
| DSTN | LOC729088 |  |  | CSPG5 | LOC283454 | RPS6KA6 | WIPF3 |
| DT | LOC729570 |  |  | CSRNP1 | LOC283486 | RPS6KL1 | WIPI1 |
| DTWD1 | LOC730102 |  |  | CSRNP2 | LOC283516 | RQCD1 | WISP1 |
| DTWD2 | LOC730236 |  |  | CSRP3 | LOC283624 | RRAGC | WNT16 |
| DUS4L | LOC731789 |  |  | CSTF2 | LOC283692 | RRAS | WNT9A |
| DUSP11 | LOC731884 |  |  | CTA-216E10.6 | LOC283887 | RRAS2 | WWC1 |
| DUSP12 | LOH12CR2 |  |  | CTGF | LOC283999 | RRBP1 | WWC3 |
| DUSP14 | LPAR5 |  |  | CTHRC1 | LOC284219 | RRM2 | WWTR1 |
| DUSP22 | LPCAT2 |  |  | CTNND2 | LOC284297 | RRP12 | XIRP1 |
| DUT | LPCAT4 |  |  | CTPS | LOC284454 | RSC1A1 | XPO4 |
| DYNC1I2 | LPHN1 |  |  | CTSA | LOC284632 | RSPH3 | XPO5 |
| DYNLRB2 | LPHN2 |  |  | CTSB | LOC284751 | RSPH9 | XPR1 |
| DYRK2 | LPHN3 |  |  | CTSD | LOC284788 | RTN3 | XRCC4 |
| DYRK3 | LPXN |  |  | CTSZ | LOC284889 | RTN4IP1 | YAF2 |
| DZIP1 | LRIG3 |  |  | CTTN | LOC284898 | RUNDC1 | YDJC |
|  | LRP1 |  |  | CTTNBP2NL | LOC285370 | RUNDC3A | YIF1A |
|  | LRP11 |  |  | CTXN3 | LOC285423 | RXFP1 | YLPM1 |
|  | LRP4 |  |  | CUX1 | LOC285577 | RYR3 | YPEL1 |
|  | LRRC2 |  |  | CUX2 | LOC285889 |  | YPEL2 |
|  | LRRC37A2 |  |  | CXCL16 | LOC286121 |  | YWHAB |
|  | LRRC39 |  |  | CXCL2 | LOC286154 |  | YWHAH |
|  | LRRC40 |  |  | CXCR4 | LOC286434 |  | YWHAQ |
|  | LRRC58 |  |  | CXCR7 | LOC338579 |  | ZAR1 |
|  | LRRC66 |  |  | CXorf27 | LOC338651 |  | ZBTB47 |
|  | LRRK2 |  |  | CXorf36 | LOC338694 |  | ZC3H12A |
|  | LRRN3 |  |  | CXorf64 | LOC338758 |  | ZC3H15 |
|  | LSM14A |  |  | CYB5R1 | LOC339240 |  | ZC3HAV1 |
|  | LSM5 |  |  | CYB5R3 | LOC339260 |  | ZCCHC10 |
|  | LTBP4 |  |  | CYLC1 | LOC339352 |  | ZCCHC17 |
|  | LUC7L3 |  |  | CYLD | LOC339622 |  | ZDHHC1 |
|  | LY75 |  |  | CYMP | LOC339803 |  | ZDHHC12 |
|  | LYG1 |  |  | CYP11A1 | LOC339874 |  | ZDHHC14 |
|  | LYPLA1 |  |  | CYP19A1 | LOC339894 |  | ZDHHC16 |
|  | LYPLAL1 |  |  | CYP1B1 | LOC339929 |  | ZDHHC2 |
|  | LYRM1 |  |  | CYP27B1 | LOC341912 |  | ZDHHC23 |
|  | LYRM5 |  |  | CYP2A13 | LOC388210 |  | ZDHHC3 |
|  | LYRM7 |  |  | CYP2A6 | LOC399959 |  | ZDHHC5 |
|  | LYSMD3 |  |  | CYP2C18 | LOC400236 |  | ZFAND3 |
|  |  |  |  | CYP2J2 | LOC400238 |  | ZFP106 |
|  |  |  |  | CYP46A1 | LOC400654 |  | ZFP91 |
|  |  |  |  | CYP4F11 | LOC400684 |  | ZFR |
|  |  |  |  | CYP4F2 | LOC400748 |  | ZFYVE16 |
|  |  |  |  | CYR61 | LOC400768 |  | ZMYND10 |
|  |  |  |  | CYTH2 | LOC401068 |  | ZMYND11 |
|  |  |  |  | CYTSA | LOC401387 |  | ZMYND12 |
|  |  |  |  | D21S2089E | LOC439914 |  | ZMYND17 |
|  |  |  |  | DAB2IP | LOC439990 |  | ZNF19 |
|  |  |  |  | DACT1 | LOC440995 |  | ZNF259 |
|  |  |  |  | DACT3 | LOC441086 |  | ZNF259P |
|  |  |  |  | DAP | LOC441208 |  | ZNF280B |
|  |  |  |  | DAPK3 | LOC442113 |  | ZNF326 |
|  |  |  |  | DARS2 | LOC494558 |  | ZNF330 |
|  |  |  |  | DAZAP2 | LOC541471 |  | ZNF35 |
|  |  |  |  | DBN1 | LOC550113 |  | ZNF367 |
|  |  |  |  | DBNL | LOC642852 |  | ZNF408 |
|  |  |  |  | DCAF4L2 | LOC643201 |  | ZNF469 |
|  |  |  |  | DCAF5 | LOC643733 |  | ZNF483 |
|  |  |  |  | DCAKD | LOC643923 |  | ZNF551 |
|  |  |  |  | DCBLD2 | LOC644714 |  | ZNF556 |
|  |  |  |  | DCTN1 | LOC644844 |  | ZNF560 |
|  |  |  |  | DCTN5 | LOC645984 |  | ZNF563 |
|  |  |  |  | DCUN1D3 | LOC646324 |  | ZNF576 |
|  |  |  |  | DCXR | LOC646808 |  | ZNF610 |
|  |  |  |  | DDA1 | LOC727916 |  | ZNF613 |
|  |  |  |  | DDAH1 | LOC727924 |  | ZNF620 |
|  |  |  |  | DDAH2 | LOC728353 |  | ZNF695 |
|  |  |  |  | DDB2 | LOC728730 |  | ZNF750 |
|  |  |  |  | DDO | LOC729085 |  | ZNF804B |
|  |  |  |  | DDR1 | LOC729680 |  | ZNF81 |
|  |  |  |  | DDX24 | LOC729993 |  | ZNRF2 |
|  |  |  |  | DDX28 | LOC730098 |  | ZSCAN23 |
|  |  |  |  | DDX43 | LOC730961 |  | ZWILCH |
|  |  |  |  | DDX53 | LOC731223 |  | ZWINT |
|  |  |  |  | DDX54 | LOC731852 |  | ZYG11A |
|  |  |  |  | DECR2 | LOC732096 |  |  |
|  |  |  |  | DEM1 | LOC81691 |  |  |
|  |  |  |  | DENND1A | LOC90586 |  |  |
|  |  |  |  | DENND5A | LOC90784 |  |  |
|  |  |  |  | DEPDC1 | LONP2 |  |  |
|  |  |  |  | DEPDC1B | LOX |  |  |
|  |  |  |  | DERL1 | LOXHD1 |  |  |
|  |  |  |  | DES | LOXL1 |  |  |
|  |  |  |  | DFFA | LOXL2 |  |  |
|  |  |  |  | DF5 | LOXL3 |  |  |
|  |  |  |  | DGCR11 | LPAL2 |  |  |
|  |  |  |  | DGCR7 | LPCAT3 |  |  |
|  |  |  |  | DGCR9 | LPGAT1 |  |  |
|  |  |  |  | DGKD | LPL |  |  |
|  |  |  |  | DGKI | LRCH1 |  |  |
|  |  |  |  | DHDDS | LRFN3 |  |  |
|  |  |  |  | DHDPSL | LRP2BP |  |  |
|  |  |  |  | DHH | LRP8 |  |  |
|  |  |  |  | DHRS7B | LRPAP1 |  |  |
|  |  |  |  | DHX32 | LRRC1 |  |  |
|  |  |  |  | DHX35 | LRRC14 |  |  |
|  |  |  |  | DHX8 | LRRC17 |  |  |
|  |  |  |  | DIAPH1 | LRRC18 |  |  |
|  |  |  |  | DIAPH3 | LRRC23 |  |  |
|  |  |  |  | DIRAS2 | LRRC36 |  |  |
|  |  |  |  | DKFZp434H1419 | LRRC42 |  |  |
|  |  |  |  | DKFZp451B082 | LRRC56 |  |  |
|  |  |  |  | DKFZp686O24166 | LRRC59 |  |  |
|  |  |  |  | DLG1 | LRRC6 |  |  |
|  |  |  |  | DLG3 | LRRC70 |  |  |
|  |  |  |  | DLGAP4 | LRRC8A |  |  |
|  |  |  |  | DLGAP5 | LRRIQ3 |  |  |
|  |  |  |  | DLK2 | LSM14B |  |  |
|  |  |  |  | DLX4 | LTBP1 |  |  |
|  |  |  |  | DMD | LTBP2 |  |  |
|  |  |  |  | DMWD | LTF |  |  |
|  |  |  |  | DH1 | LUZP1 |  |  |
|  |  |  |  | DH2 | LYAR |  |  |
|  |  |  |  | DI1 | LYNX1 |  |  |
|  |  |  |  | DJA1 | LYPD1 |  |  |
|  |  |  |  | DJA4 | LYRM4 |  |  |
|  |  |  |  | DJB4 | LZTS1 |  |  |
|  |  |  |  | DJB5 |  |  |  |
|  |  |  |  | DJC13 |  |  |  |
|  |  |  |  | DJC15 |  |  |  |
|  |  |  |  | DJC30 |  |  |  |
|  |  |  |  | DL1 |  |  |  |
|  |  |  |  | DSE2 |  |  |  |
|  |  |  |  | DOCK1 |  |  |  |
|  |  |  |  | DOCK5 |  |  |  |
|  |  |  |  | DOK1 |  |  |  |
|  |  |  |  | DOK4 |  |  |  |
|  |  |  |  | DOK7 |  |  |  |
|  |  |  |  | DPF1 |  |  |  |
|  |  |  |  | DPH3 |  |  |  |
|  |  |  |  | DPT |  |  |  |
|  |  |  |  | DPYSL3 |  |  |  |
|  |  |  |  | DRD1 |  |  |  |
|  |  |  |  | DSCR6 |  |  |  |
|  |  |  |  | DTD1 |  |  |  |
|  |  |  |  | DTL |  |  |  |
|  |  |  |  | DTNB |  |  |  |
|  |  |  |  | DTX4 |  |  |  |
|  |  |  |  | DTYMK |  |  |  |
|  |  |  |  | DUSP1 |  |  |  |
|  |  |  |  | DUSP10 |  |  |  |
|  |  |  |  | DUSP15 |  |  |  |
|  |  |  |  | DUSP16 |  |  |  |
|  |  |  |  | DUSP26 |  |  |  |
|  |  |  |  | DUSP27 |  |  |  |
|  |  |  |  | DUSP3 |  |  |  |
|  |  |  |  | DUSP5 |  |  |  |
|  |  |  |  | DUSP6 |  |  |  |
|  |  |  |  | DUSP8 |  |  |  |
|  |  |  |  | DYNC1LI2 |  |  |  |
|  |  |  |  | DYNLL1 |  |  |  |
|  |  |  |  | DYNLT3 |  |  |  |
|  |  |  |  | DYSF |  |  |  |

***** Transcript originally coded as a number.
